# Genome-wide association study and functional characterisation identifies candidate genes for insulin-stimulated glucose uptake

**DOI:** 10.1038/s41588-023-01408-9

**Published:** 2023-06-08

**Authors:** Alice Williamson, Dougall M Norris, Xianyong Yin, K. Alaine Broadaway, Anne H Moxley, Swarooparani Vadlamudi, Emma P Wilson, Anne U Jackson, Vasudha Ahuja, Mette K Andersen, Zorayr Arzumanyan, Lori L Bonnycastle, Stefan R Bornstein, Maxi P. Bretschneider, Thomas A Buchanan, Yi-Cheng Chang, Lee-Ming Chuang, Ren-Hua Chung, Tine D Clausen, Peter Damm, Graciela E Delgado, Vanessa D de Mello, Josée Dupuis, Om P Dwivedi, Michael R Erdos, Lilian Fernandes Silva, Timothy M Frayling, Christian Gieger, Mark O Goodarzi, Xiuqing Guo, Stefan Gustafsson, Liisa Hakaste, Ulf Hammar, Gad Hatem, Sandra Herrmann, Kurt Højlund, Katrin Horn, Willa A Hsueh, Yi-Jen Hung, Chii-Min Hwu, Anna Jonsson, Line L Kårhus, Marcus E Kleber, Peter Kovacs, Timo A Lakka, Marie Lauzon, I-Te Lee, Cecilia M Lindgren, Jaana Lindström, Allan Linneberg, Ching-Ti Liu, Jian'an Luan, Dina Mansour Aly, Elisabeth Mathiesen, Angela P Moissl, Andrew P Morris, Narisu Narisu, Nikolaos Perakakis, Annette Peters, Rashmi B Prasad, Roman N Rodionov, Kathryn Roll, Carsten F Rundsten, Chloé Sarnowski, Kai Savonen, Markus Scholz, Sapna Sharma, Sara E Stinson, Sufyan Suleman, Jingyi Tan, Kent D Taylor, Matti Uusitupa, Dorte Vistisen, Daniel R Witte, Romy Walther, Peitao Wu, Anny H Xiang, Björn Zethelius, The Meta-Analysis of Glucose and Insulin-related Traits Consortium (MAGIC), Alice Williamson, Xianyong Yin, K. Alaine Broadaway, Emma P Wilson, Anne U Jackson, Vasudha Ahuja, Mette K Andersen, Zorayr Arzumanyan, Lori L Bonnycastle, Stefan R Bornstein, Maxi P. Bretschneider, Thomas A Buchanan, Yi-Cheng Chang, Lee-Ming Chuang, Ren-Hua Chung, Tine D Clausen, Peter Damm, Graciela E Delgado, Vanessa D de Mello, Josée Dupuis, Om P Dwivedi, Michael R Erdos, Lilian Fernandes Silva, Timothy M Frayling, Christian Gieger, Mark O Goodarzi, Xiuqing Guo, Stefan Gustafsson, Liisa Hakaste, Ulf Hammar, Gad Hatem, Sandra Herrmann, Kurt Højlund, Katrin Horn, Willa A Hsueh, Yi-Jen Hung, Chii-Min Hwu, Anna Jonsson, Line L Kårhus, Marcus E Kleber, Peter Kovacs, Timo A Lakka, Marie Lauzon, I-Te Lee, Cecilia M Lindgren, Jaana Lindström, Allan Linneberg, Ching-Ti Liu, Jian'an Luan, Dina Mansour Aly, Elisabeth Mathiesen, Angela P Moissl, Andrew P Morris, Narisu Narisu, Nikolaos Perakakis, Annette Peters, Rashmi B Prasad, Roman N Rodionov, Kathryn Roll, Carsten F Rundsten, Chloé Sarnowski, Kai Savonen, Markus Scholz, Sapna Sharma, Sara E Stinson, Sufyan Suleman, Jingyi Tan, Kent D Taylor, Matti Uusitupa, Dorte Vistisen, Daniel R Witte, Romy Walther, Peitao Wu, Anny H Xiang, Björn Zethelius, Emma Ahlqvist, Richard N Bergman, Yii-Der Ida Chen, Francis S Collins, Tove Fall, Jose C Florez, Andreas Fritsche, Harald Grallert, Leif Groop, Torben Hansen, Heikki A Koistinen, Pirjo Komulainen, Markku Laakso, Lars Lind, Markus Loeffler, Winfried März, James B Meigs, Leslie J Raffel, Rainer Rauramaa, Jerome I Rotter, Peter E. H. Schwarz, Michael Stumvoll, Johan Sundström, Anke Tönjes, Tiinamaija Tuomi, Jaakko Tuomilehto, Robert Wagner, Inês Barroso, Mark Walker, Niels Grarup, Michael Boehnke, Nicholas J Wareham, Karen L Mohlke, Eleanor Wheeler, Stephen O’Rahilly, Claudia Langenberg, Emma Ahlqvist, Richard N Bergman, Yii-Der Ida Chen, Francis S Collins, Tove Fall, Jose C Florez, Andreas Fritsche, Harald Grallert, Leif Groop, Torben Hansen, Heikki A Koistinen, Pirjo Komulainen, Markku Laakso, Lars Lind, Markus Loeffler, Winfried März, James B Meigs, Leslie J Raffel, Rainer Rauramaa, Jerome I Rotter, Peter E. H. Schwarz, Michael Stumvoll, Johan Sundström, Anke Tönjes, Tiinamaija Tuomi, Jaakko Tuomilehto, Robert Wagner, Inês Barroso, Mark Walker, Niels Grarup, Michael Boehnke, Nicholas J Wareham, Karen L Mohlke, Eleanor Wheeler, Stephen O’Rahilly, Daniel J Fazakerley, Claudia Langenberg

**Affiliations:** 1 MRC Epidemiology Unit, Institute of Metabolic Science, University of Cambridge School of Clinical Medicine, Cambridge, UK; 2 Metabolic Research Laboratories, Wellcome Trust-MRC Institute of Metabolic Science, Department of Clincial Biochemistry, University of Cambridge, Cambridge, UK; 3 Metabolic Research Laboratories, Wellcome Trust-MRC Institute of Metabolic Science, Department of Clinical Biochemistry, University of Cambridge, Cambridge, UK; 4 Department of Biostatistics, University of Michigan, Ann Arbor, MI, USA; 5 Center for Statistical Genetics, University of Michigan, Ann Arbor, MI, USA; 6 Department of Epidemiology, School of Public Health, Nanjing Medical University, Nanjing, Jiangsu 211166, China; 7 Department of Genetics, University of North Carolina, Chapel Hill, NC, USA; 8 Institute for Molecular Medicine Finland (FIMM), University of Helsinki, Helsinki, Finland; 9 Novo Nordisk Foundation Center for Basic Metabolic Research, Faculty of Health and Medical Sciences, University of Copenhagen, Copenhagen, Denmark; 10 Department of Pediatrics, Genomic Outcomes, The Institute for Translational Genomics and Population Sciences, The Lundquist Institute for Biomedical Innovation at Harbor-UCLA Medical Center, Torrance, CA, USA; 11 Center for Precision Health research, National Human Genome Research Institute, National Institutes of Health, Bethesda, MD, USA; 12 Department of Internal Medicine III, Metabolic and Vascular Medicine, Medical Faculty Carl Gustav Carus, Dresden, Germany; 13 Helmholtz Zentrum München, Paul Langerhans Institute Dresden (PLID), University Hospital and Faculty of Medicine, TU Dresden, Dresden, Germany; 14 German Center for Diabetes Research (DZD e.V.), Neuherberg, Germany; 15 Department of Medicine, Division of Endocrinology and Diabetes, Keck School of Medicine USC, Los Angeles, CA, USA; 16 Graduate Institute of Medical Genomics and Proteomics, National Taiwan University, Taipei City, Taiwan; 17 Internal Medicine, National Taiwan University Hospital, Taipei City, Taiwan; 18 Institute of Biomedical Sciences, Academia Sinica, Taipei City, Taiwan; 19 Department of Internal Medicine, Division of Endocrinology and Metabolism, National Taiwan University Hospital, Taipei City, Taiwan; 20 Institute of Population Health Sciences, National Health Research Institutes, Miaoli County, Taiwan; 21 Department of Gynecology and Obstetrics, Nordsjaellands Hospital, Hilleroed, Denmark; 22 Department of Clinical Medicine, Faculty of Health and Medical Sciences, University of Copenhagen, Copenhagen, Denmark; 23 Center for Pregnant Women with Diabetes, Rigshospitalet, Copenhagen, Denmark; 24 Department of Obstetrics, Rigshospitalet, Copenhagen, Denmark; 25 Vth Department of Medicine, Medical Faculty Mannheim, Heidelberg University, Mannheim, BW, Germany; 26 Institute of Public Health and Clinical Nutrition, University of Eastern Finland, Kuopio, Finland; 27 Department of Biostatistics, Boston University School of Public Health, Boston, MA, USA; 28 Department of Epidemiology, Biostatistics and Occupational Health, McGill University, Montréal, Quebec, Canada; 29 Institute of Clinical Medicine, University of Eastern Finland, Kuopio, Finland; 30 University of Exeter Medical School, University of Exeter, Exeter, UK; 31 Institute of Epidemiology II, Helmholtz Zentrum München-German Research Center for Environmental Health, Neuherberg, Germany; 32 Department of Medicine, Division of Endocrinology, Diabetes & Metabolism, Cedars-Sinai Medical Center, Los Angeles, CA, USA; 33 Department of Medical Sciences, Clinical Epidemiology, Uppsala University, Uppsala, Sweden; 34 Department of Medical Sciences, Molecular Epidemiology, Uppsala University, Uppsala, Sweden; 35 Clinical Sciences Malmö, Genomics, Diabetes and Endocrinology, Lund University, Malmö, Sweden; 36 Department of Internal Medicine III, Prevention and Care of Diabetes, Medical Faculty Carl Gustav Carus, Dresden, Germany; 37 Steno Diabetes Center Odense, Odense University Hospital, Odense, Denmark; 38 Medical Faculty, Institute for Medical Informatics, Statistics and Epidemiology, Leipzig, Germany; 39 LIFE Research Center for Civilization Diseases, Medical Faculty, Leipzig, Germany; 40 Internal Medicine, Endocrinology, Diabetes & Metabolism, The Ohio State University Wexner Medical Center, Columbus, OH, USA; 41 Institute of Preventive Medicine, National Defense Medical Center, New Taipei City, Taiwan; 42 Department of Medicine, Section of Endocrinology and Metabolism, Taipei Veterans General Hospital, Taipei City, Taiwan; 43 Center for Clinical Research and Prevention, Copenhagen University Hospital – Bispebjerg and Frederiksberg, Copenhagen, Denmark; 44 Vth Department of Medicine, Medical Faculty Mannheim, Heidelberg University, Mannheim, Germany; 45 SYNLAB MVZ Humangenetik Mannheim, Mannheim, Germany; 46 Medical Department III-Endocrinology, Nephrology, Rheumatology, University of Leipzig Medical Center, Leipzig, Germany; 47 Institute of Biomedicine, School of Medicine, University of Eastern Finland, Kuopio, Finland; 48 Department of Clinical Physiology and Nuclear Medicine, Kuopio University Hospital, Kuopio, Finland; 49 Foundation for Research in Health Exercise and Nutrition, Kuopio Research Institute of Exercise Medicine, Kuopio, Finland; 50 Department of Pediatrics, Genomic Outcomes, The Institute for Translational Genomics and Population Sciences, The Lundquist Institute at Harbor-UCLA Medical Center, Torrance, CA, USA; 51 Department of Internal Medicine, Division of Endocrinology and Metabolism, Taichung Veterans General Hospital, Taichung City, Taiwan; 52 School of Medicine, National Yang Ming Chiao Tung University, Taipei City, Taiwan; 53 School of Medicine, Chung Shan Medical University, Taichung City, Taiwan; 54 Big Data Institute, Li Ka Shing Centre for Health Information and Discovery, University of Oxford, Oxford, UK; 55 Nuffield Department of Population Health, University of Oxford, Oxford, UK; 56 Wellcome Trust Centre Human Genetics, University of Oxford, Oxford, UK; 57 Broad Institute, Cambridge, MA, USA; 58 Finnish Institute for Health and Welfare, Helsinki, Finland; 59 Department of Endocrinology, Rigshospitalet, Copenhagen, Denmark; 60 Institute of Nutritional Sciences, Friedrich-Schiller-University, Jena, Germany; 61 Competence Cluster for Nutrition and Cardiovascular Health (nutriCARD) Halle-Jena, Jena, Germany; 62 Centre for Genetics and Genomics Versus Arthritis, Centre for Musculoskeletal Research, The University of Manchester, Manchester, UK; 63 Department of Internal Medicine III, University Center for Vascular Medicine, Medical Faculty Carl Gustav Carus, Dresden, Germany; 64 College of Medicine and Public Health, Flinders University and Flinders Medical Centre,, Adelaide, Australia; 65 Pediatrics, Genomic Outcomes, The Institute for Translational Genomics and Population Sciences, The Lundquist Institute for Biomedical Innovation at Harbor-UCLA Medical Center, Torrance, CA, USA; 66 Epidemiology, Human Genetics & Environmental Sciences, The University of Texas Health Science Center, Houston, TX, USA; 67 Research Unit of Molecular Epidemiology, Helmholtz Zentrum München-German Research Center for Environmental Health, Neuherberg, Germany; 68 Chair of Food Chemistry and Molecular and Sensory Science, Technical University of Munich, Freising-Weihenstephan, Germany; 69 Department of Public Health and Clinical Nutrition, University of Eastern Finland, Kuopio, Finland; 70 Clinical Research, Steno Diabetes Center Copenhagen, Herlev, Denmark; 71 Department of Public Health, University of Copenhagen, Copenhagen, Denmark; 72 Steno Diabetes Center Aarhus, Aarhus, Denmark; 73 Department of Public Health, Aarhus University, Aarhus, Denmark; 74 Department of Internal Medicine III, Pathobiochemistry, Medical Faculty Carl Gustav Carus, Dresden, Germany; 75 Department of Biostatistics, Boston University School of Public Health, Boston, MA, 2118; 76 Research & Evaluation, Division of Biostatistics, Kaiser Permanente Southern California, Pasadena, CA, USA; 77 Public Health and Caring Sciences, Geriatrics, Uppsala University, Uppsala, Sweden; 78 Diabetes and Obesity Research Institute, Cedars-Sinai Medical Center, Los Angeles, CA, USA; 79 Diabetes Unit and Center for Genomic Medicine, Massachusetts General Hospital, Boston, MA, USA; 80 Programs in Metabolism and Medical & Population Genetics, Broad Institute, Cambridge, MA, USA; 81 Department of Medicine, Harvard Medical School, Harvard Medical School, Boston, MA, USA; 82 Department of Internal Medicine IV, University Hospital Tübingen, Tübingen, Germany; 83 Clinical Sciences Malmö, Genomics, Diabetes and Endocrinology, Lund University, Lund, Sweden; 84 Department of Public Health and Welfare, Finnish Institute for Health and Welfare, Helsinki, Finland; 85 Department of Medicine, University of Helsinki and Helsinki University Hospital, Helsinki, Finland; 86 Minerva Foundation Institute for Medical Research, Helsinki, Finland; 87 Synlab Academy, SYNLAB Holding Deutschland GmbH, Mannheim, BW, Germany; 88 Department of Medicine, Division of General Internal Medicine, Massachusetts General Hospital, Boston, MA, USA; 89 Department of Medicine, Harvard Medical School, Boston, MA, USA; 90 Programs in Metabolism and Medical & Population Genetics, The Broad Institute, Cambridge, MA, USA; 91 Department of Pediatrics, Genetic and Genomic Medicine, University of California, Irvine, CA, USA; 92 The Institute for Translational Genomics and Population Sciences, Department of Pediatrics, The Lundquist Institute for Biomedical Innovation at Harbor-UCLA Medical Center, Torrance, CA, USA; 93 Folkhälsan Research Center, Helsinki, Finland; 94 Department of Public Health, University of Helsinki, Helsinki, Finland; 95 Population Health Unit, Finnish Institute for Health and Welfare, Helsinki, Finland; 96 Diabetes Research Group, King Abdulaziz University, Jeddah, Saudi Arabia; 97 Exeter Centre of Excellence for Diabetes Research (EXCEED), Genetics of Complex Traits, University of Exeter Medical School, University of Exeter, Exeter, UK; 98 Faculty of Medical Sciences, Newcastle University, Newcastle upon Tyne, UK; 99 Computational Medicine, Berlin Institute of Health at Charité– Universitätsmedizin, Berlin, Germany; 100 Precision Healthcare University Research Institute, Queen Mary University of London, London, UK

## Abstract

Distinct tissue-specific mechanisms mediate insulin action in fasting and postprandial states. Previous genetic studies have largely focused on insulin resistance in the fasting state, where hepatic insulin action dominates. Here we studied genetic variants influencing insulin levels measured 2 hours after a glucose challenge in >55,000 participants from three ancestry groups. We identified 10 novel loci (P<5x10^-8^) not previously associated with post-challenge insulin resistance, 8 of which were shown to share their genetic architecture with type 2 diabetes in colocalization analyses. We investigated candidate genes at a subset of associated loci in cultured cells and identified 9 candidate genes newly implicated in the expression or trafficking of GLUT4, the key glucose transporter in postprandial glucose uptake in muscle and fat. By focusing on post-prandial insulin resistance, we highlight mechanisms of action at T2D loci that are not adequately captured by studies of fasting glycaemic traits.

## Introduction

Insulin resistance is a state in which higher than normal levels of circulating insulin are required to maintain glucose homeostasis and compensate for decreased insulin sensitivity of key organs involved in metabolic control – liver, muscle, and fat^1^. If the pancreas fails to maintain the elevated insulin supply and glucose uptake into these tissues becomes insufficient, hyperglycaemia and impaired glucose tolerance can subsequently lead to the development of T2D^2^ and related cardiometabolic disorders^1^.

The mechanisms and tissues of action of insulin action are distinct in the fasting and postprandial state and therefore the aetiology of insulin resistance in the fasting and fed state differs across tissues. Most genetic studies of common forms of insulin resistance have focused on the fasting state, which largely reflects hepatic insulin sensitivity.^3–5^ In the liver, insulin controls the expression and post translational modification of genes controlling glycogenolysis and gluconeogenesis, thereby supressing hepatic glucose output and the promotion of glycogen synthesis.^6–8^ Postprandially, insulin rapidly stimulates the translocation of an intracellular pool of the glucose transporter GLUT4 (encoded by *SLC2A4)* to the cell surface in muscle and fat ^9–11^ Insulin resistance is associated with lower GLUT4 expression and translocation in adipose tissue^12^, and impaired GLUT4 translocation in muscle. ^13^ This postprandial distrubance in insulin action has been identified to be one of the key initating steps to onset of T2D^14^, however the genetic underprinnings of this have been understudied.

There are currently only a few examples of loss of function coding variants that contribute to tissue-specific changes in postprandial insulin action. A loss of function mutation in *TBC1D4*, encoding a Rab-GTPase activating protein involved in control of GLUT4 translocation^15^, was first reported to co-segregate with severe isolated post-prandial hyperinsulinaemia in a single family^16^. Subsequently a different loss of function mutation in a muscle specific isoform of *TBC1D4* was found to be common in Inuit populations with carriers having selective postprandial hyperglycaemia and hyperinsulinemia^17,18^. Thus, deeper understanding of the genetic architecture of tissue-specific insulin resistance in the fed state is needed to further the understanding of this process in T2D aetiology.

The gold standard method of measuring insulin resistance in these GLUT4-expressing tissues is the hyperinsulinemic-euglycaemic clamp combined with isotope-based measurement of glucose clearance. Such measures are not readily available at the scale required for robust genome-wide analyses^19,20^. Proxy measures derived from insulin and glucose measured during an oral glucose tolerance test (OGTT) have been developed^21,22^, and are available in much greater numbers.

Here, we studied two OGTT-derived measures of insulin resistance after a glucose challenge (subsequently referred to as post-challenge insulin resistance) to identify genetic variants underlying these traits in a genome-wide meta-analysis (GWAS) of >55,000 participants without diabetes from 3 ancestry groups across 28 studies. These measures were the Modified Stumvoll Insulin Sensitivity Index (ISI) and the fold-change in insulin concentration (insulin fold-change; IFC) following an OGTT.

IFC reflects the relationship between fasting and post challenge plasma insulin Higher IFC values suggest more insulin is required to lower blood glucose after a standard glucose load and is therefore indicative of post-challenge insulin resistance This is dramatically increased in carriers of loss of function mutations in *TBC1D4* that impair glucose transporter translocation^16–18^. IFC therefore can be leveraged as an index of post-challenge, rather than fasting, insulin resistance.

ISI incorporates measures of insulin and glucose during an OGTT and has been demonstrated to approximate hyperinsulinemic-euglycaemic measures of whole-body insulin sensitivity^22^. Lower values of ISI reflect greater insulin resistance. Previous meta-analysis efforts have examined the genetic architecture of ISI^23,24^. Whilst no genome wide significant loci were identified by Dimas *et al* (2014)^24^, Walford *et al* (2016) identified 3 loci significantly associated with ISI (P < 5x10^-8^; N = 16,753) ^23^. These include *BCL2* (rs12454712) and *NYAP2* (rs13422522; in Linkage Disequilibrium (LD) with previously known *IRS1* locus rs2943641). The *FAM19A2* locus (rs10506418) was identified to be significantly associated only in an interaction model testing the effect of genotype on ISI by body mass index (BMI), which we have not explored in this present study.^23^ Our expanded meta-analysis is >3x increased in sample size from this previous meta-analysis ^23^.

**Extended Data Figure 1:**
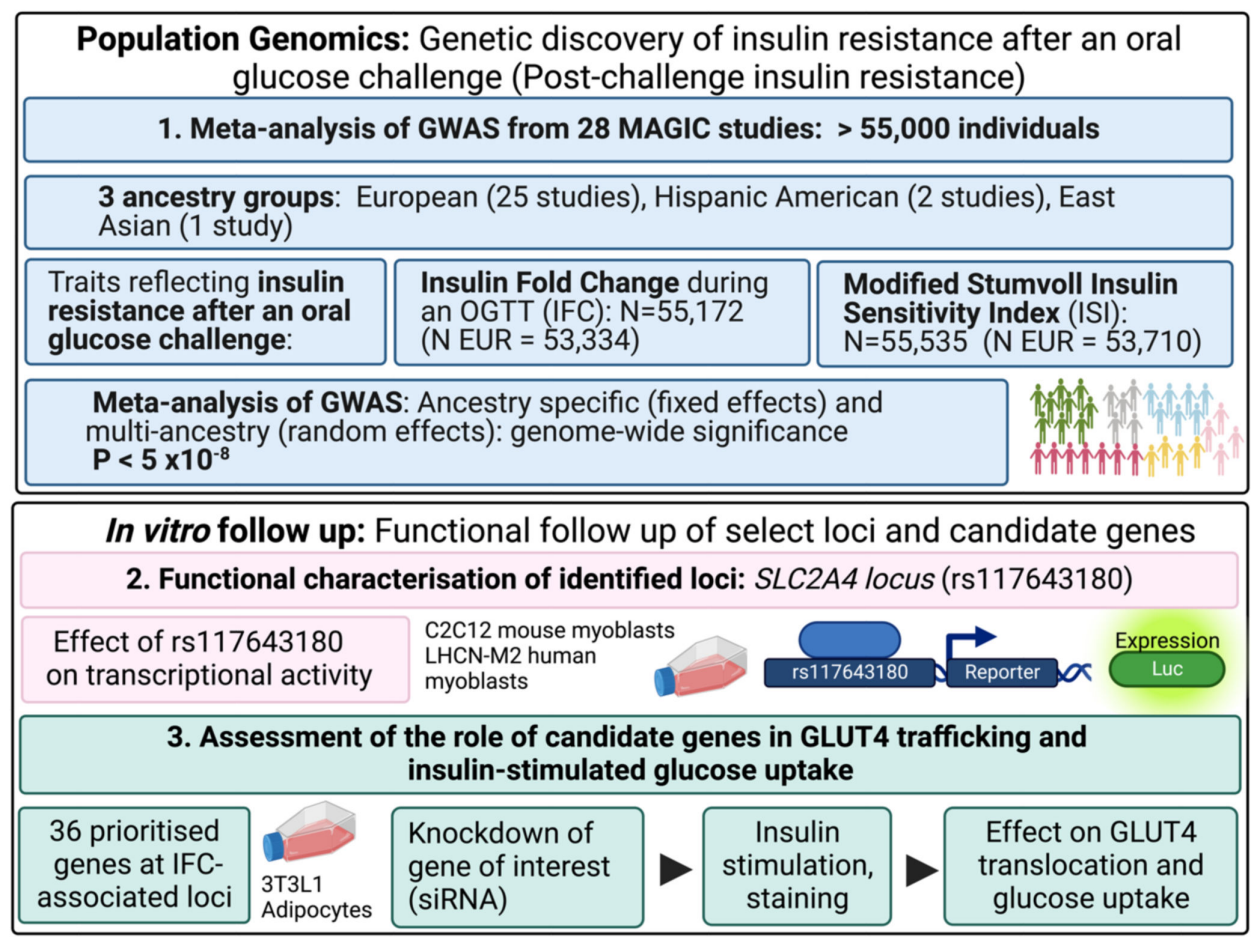
Overview of genetic discovery for insulin fold change and Modified Stumvoll ISI, and downstream genetic and in vitro studies. Created with BioRender.com.

Using a genetically driven approach, we identified 9 novel genetic loci specifically implicated in post-challenge insulin resistance in cohorts of European ancestry. This includes 4 novel loci for post-challenge insulin resistance significantly (P < 5x10^-8^) associated with IFC and 6 novel loci for post-challenge insulin resistance associated with ISI, with one locus associated with both traits. We systematically selected 36 genes from each locus associated with IFC and assessed the functional impact of reducing the expression of candidate genes from each locus in an insulin responsive cell-based reporter system that examines the translocation and abundance of GLUT4 protein (Extended Data Figure 1). This approach identified 9 candidate genes not previously implicated in insulin-regulated glucose uptake.

## Results

### Overview

**Extended Data Figure 2:**
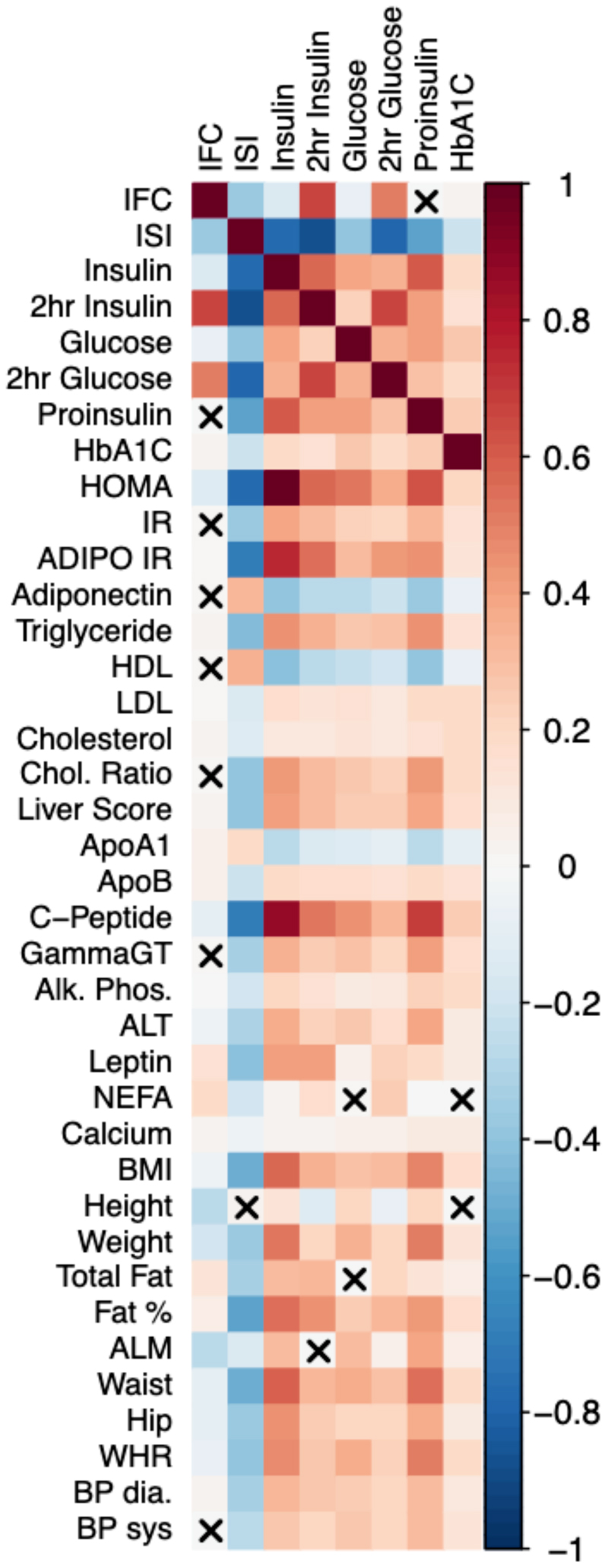
Observational Correlation of Insulin Fold Change and Modified Stumvoll ISI with metabolic traits in the Fenland Study. Pairwise Spearman’s Rank correlation. Red shades denote positive correlation, blue shared denote negative correlation between trait pairs. X denotes no significant correlation (P > 0.05).

**Extended Data Figure 3:**
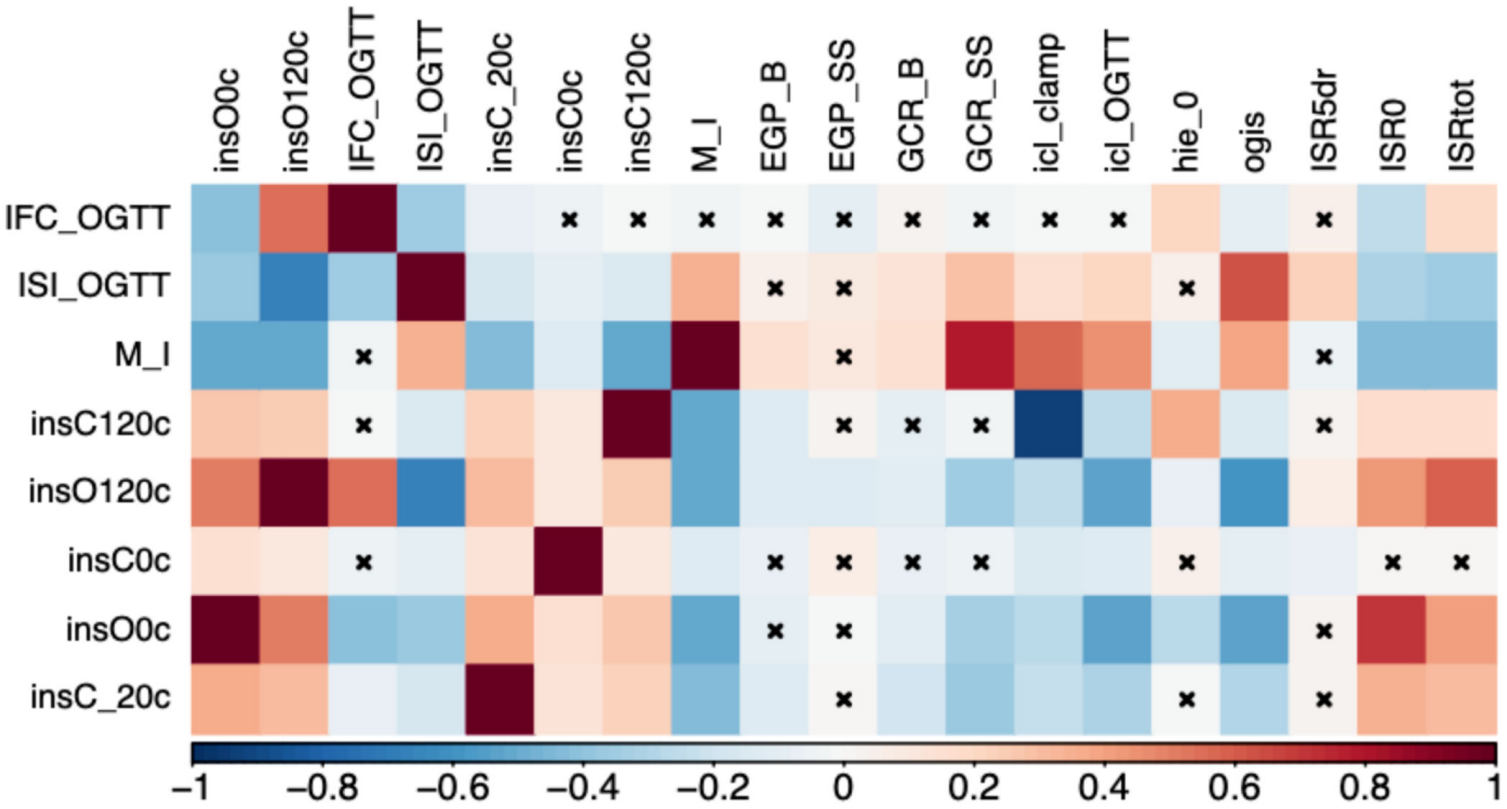
Observational Correlation of insulin sensitivity and clearance related traits in the RISC study. Pairwise Spearman’s Rank correlation. Red shades denote positive correlation, blue shared denote negative correlation between trait pairs, legend along bottom of heatmap shows colour scale relative to rho value. X denotes no significant correlation (P > 0.05). Abbreviations denote: IFC_OGTT – IFC calculated using OGTT measures, ISI_OGTT – Modified Stumvoll ISI calculated using OGTT measures. M_I – M/I index of insulin sensitivity measured by clamp. InsC0c – insulin measured at 0min during clamp. InsO0c - insulin measured at 0min during OGTT. InsC120c – insulin measured at 120min during clamp. InsO120c - insulin measured at 120min during OGTT. InsC_20c – insulin measured at 20min before clamp. EGP_B -basal glucose production, EGP_SS - glucose production during clamp, GCR_B - basal glucose clearance, ml/min/kg lean body mass, GCR_SS - steady state glucose clearance, ml/min/kg lean body mass, icl_clamp - peripheral insulin clearance (1/min/m^2^), icl_OGTT - endogenous "pre-hepatic" clearance during the OGTT, hie_0 - hepatic insulin extraction during clamp, OGIS - oral glucose insulin sensitivity index (ml min-1 m-2). ISR5dr - insulin secretion 5 mM glucose, beta cell dose response (pmol min-1m-2). ISR0 basal insulin secretion (pmol min-1m-2). ISRtot - total insulin secretion (nmol m-2).

Modified Stumvoll Insulin Sensitivity Index (ISI) and insulin fold change (IFC) were highly correlated with post-challenge measures of glucose and insulin during an OGTT in the Fenland study but were shown to capture different aspects of post-challenge insulin resistance suggesting value in studying both traits in parallel (See Supplementary Note, Extended Data Figure 2, Supplementary Table 4). In the RISC study we assessed the correlation of OGTT derived ISI and IFC with the clamp-based measure of insulin sensitivity M/I, which represents the amount of glucose metabolized per unit of plasma insulin during the clamp^25^. We identified significant observational correlations of both OGTT-derived traits with this clamp-derived measure of insulin sensitivity (ISI vs M/I Spearman’s rho = 0.522, P = 5.10x10^-76^; IFC vs M/I rho = -0.180, P = 3.29x10^-9^; Extended Data Figure 3, Supplementary Note; Supplementary Table 5). Therefore, we can approximate clamp-based measures with OGTT indices available in a greater number of studies. The weaker correlations with IFC and M/I is expected, with M/I being reflective of whole-body insulin sensitivity and not specifically post-challenge insulin sensitivity which IFC captures more explicitly. ISI and IFC were moderately correlated in the Fenland (IFC vs ISI Spearman’s rho = -0.375, P < 1 x 10^-300^) and RISC studies (IFC vs ISI Spearman’s rho = -0.458, P = 3.14x10^-57^). Therefore, ISI and IFC capture similar phenotypes, but also differing aspects of post-challenge insulin resistance, suggesting there is value in studying both traits in parallel.

**Extended Data Figure 4:**
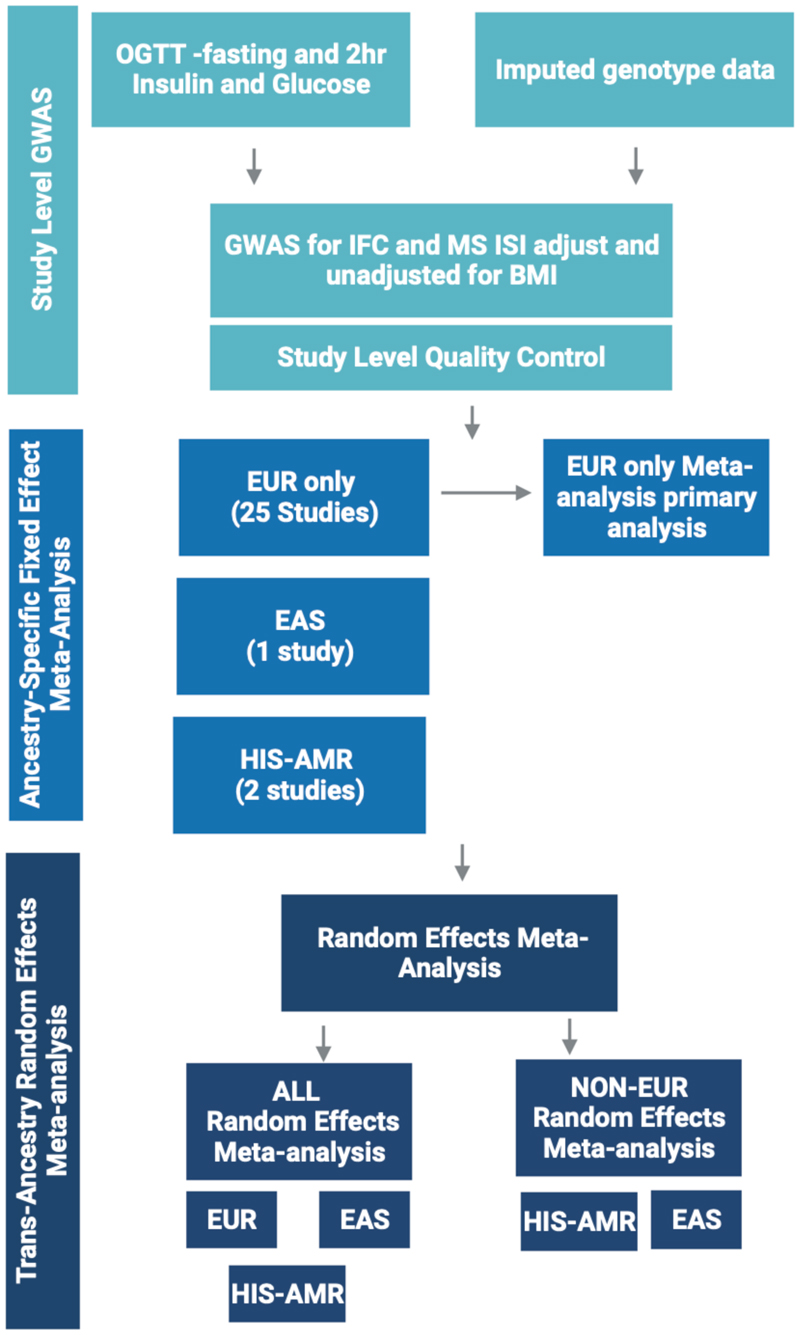
Meta-analysis workflow for genetic discovery analyses Analysis workflow for the meta-analysis of study-level GWAS results for Insulin fold change and Modified Stumvoll ISI. Created with BioRender.com

In this study we assessed the genetic architecture of post-challenge insulin resistance through meta-analysis of GWAS from a total of 28 studies of European, Hispanic American, and East Asian ancestry (Extended Data Figure 4; Supplementary Tables 1-3). We conducted ancestry specific fixed effect meta-analysis, with the primary analysis presented being the metaanalysis only of cohorts of European ancestry for IFC and ISI, adjusted for BMI. We additionally conducted random effects multi-ancestry meta-analysis across all ancestries as well as the 3 cohorts of East Asian and Hispanic American ancestry without cohorts of European ancestry.

### Genetic discovery of post-challenge insulin resistance

**Extended Data Figure 5:**
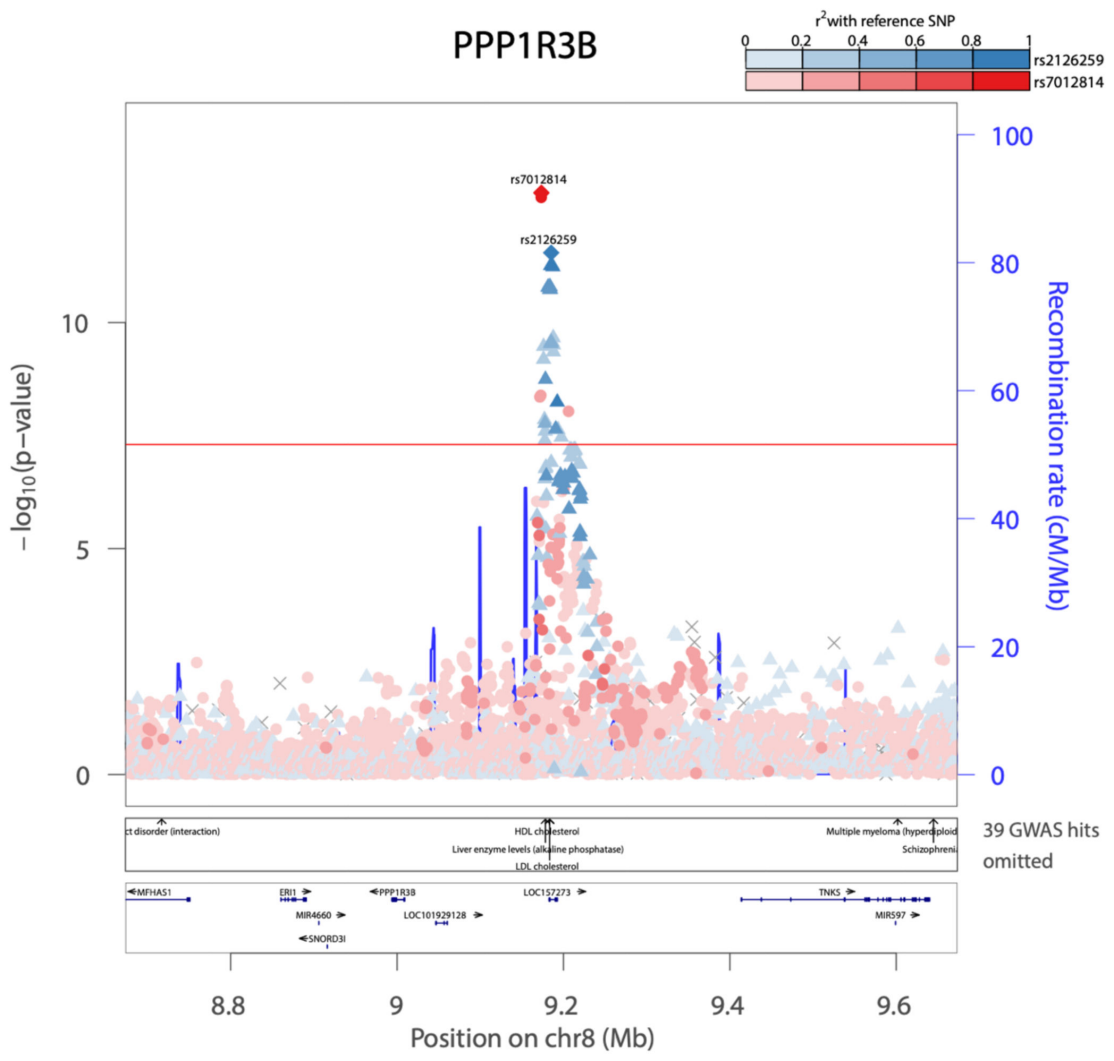
Two independent signals were identified at *PPP1R3B* for insulin fold change Conditional analyses identify a second independent signal at *PPP1R3B* for insulin fold change adjusted for BMI. The regional association plot shows the primary signal in red and the secondary signal in blue for marginal summary statistics for insulin fold change adjusted for BMI. Shade of point indicates pairwise LD (R^2^) with indicated lead variant.

**Figure 1:**
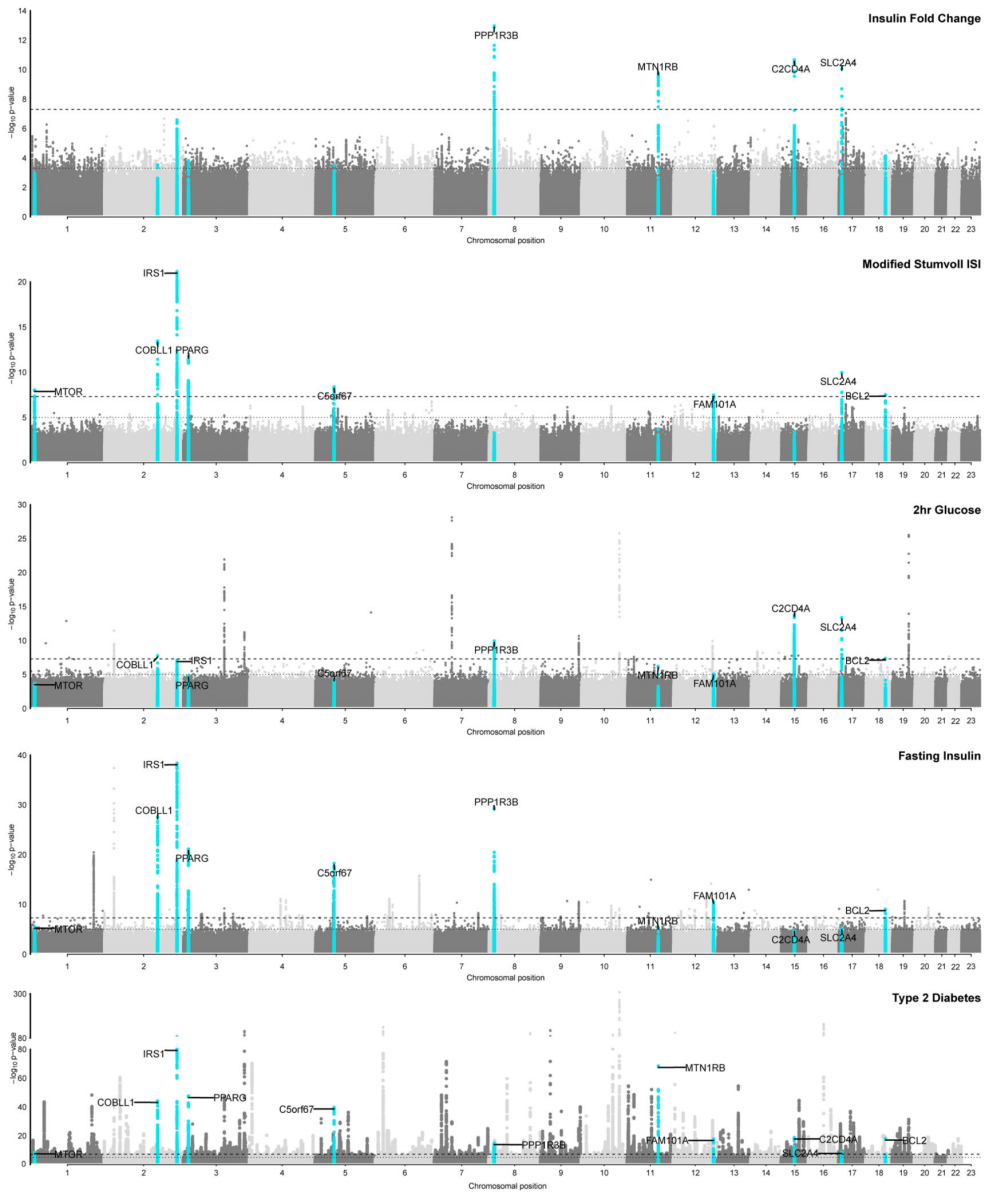
Loci associated with post-challenge insulin resistance overlap with associations with other glycaemic traits and T2D Manhattan plot of results for IFC, ISI and relative association with key glycaemic traits of interest. Y-axis denotes the -log10(p-value) of association for each trait. X-axis outlines the chromosomal position, with alternate chromosomes represented in light and dark grey. The dashed line defines genome-wide significance threshold (P = 5x10^-8^), and the dotted line denotes P = 1x10^-5^. Points highlighted in blue on each plot represent those that meet genome-wide significance for either IFC and ISI (P < 5x10^-8^). Labels for indicate the nearest gene at a locus defined at genome-wide significance for either IFC and ISI, and their relative association with other traits. Published GWAS were used for fasting insulin and 2 h glucose^3^,, and T2D^26^. For T2D y axis is cut at 1x10^-80^ for clarity, with the minimum p value truncated to P = 1x10^-300^. Uncorrected p-values are shown. All GWAS are in cohorts of European ancestry and adjusted for BMI, except T2D which is unadjusted for BMI.

We identified 11 genome-wide significant loci (P < 5x10^-8^) across both ISI and IFC in analyses adjusted for BMI in individuals without diabetes from 25 studies of European ancestry (See **methods**, Table 1, Supplementary Figures 1-4). Nine of these associations were not previously reported for post-challenge insulin resistance. Four of these loci, all not previously implicated in post-challenge insulin resistance, were significantly associated with IFC (N = 52,474; *SLC2A4, PPP1R3B, C2CD4A* and *MTNR1B;* Figure 1; Table 1; Supplementary Figures 3, 4 and 6; Supplementary Table 6 and 7). The remaining loci were associated with ISI, including 6 loci not previously implicated in post-challenge insulin resistance: *MTOR, COBLL1, PPARG, C5orf67, FAM101A, SLC2A4*. We replicated 2 previously-identified ISI loci at *IRS1* and *BCL2* (Figure 1; Table 1; Supplementary Figures 1, 2 and 5; Supplementary Table 6 and 7).^23^ Exact conditional analysis revealed 2 distinct signals at *PPP1R3B* for IFC, with no additional signals identified at other loci for either trait (Table 1; Extended Data Figure 5 ; Supplementary Tables 6 and 7). The *SLC2A4* (rs117643180) locus was the only locus significantly associated with both ISI and IFC (Supplementary Table 6 and 7).

We conducted fine mapping to define 99% credible sets that accounted for ≥99% of the posterior probability of association (PPA) for each of the 12 independent association signals (Supplementary Tables 8 and 9). At *SLC2A4* the 99% credible set included a single variant for ISI and 2 variants for IFC, with high confidence that rs117643180 is the candidate causal variant (IFC PPA for rs117643180 = 0.938; ISI PPA for rs117643180 = 0.985).

To fully assess the effect of adiposity, we repeated analyses without adjusting for BMI. IFC associations were largely unaffected by BMI adjustment (effect size R^2^ BMI adjusted vs BMI unadjusted = 0.98; Supplementary Figure 3 and 4, Supplementary Table 6 and 7). In contrast, for ISI, adjusted and unadjusted beta estimates were moderately correlated (Pearson correlation between betas = 0.78, but significance levels were weaker in BMI unadjusted models, as previously reported for other glycaemic traits^3^, with only *IRS1* and *SLC2A4* remaining significant at P < 5x10^-8^ (Supplementary Figure 1 and 2, Supplementary Table 6 and 7).

With 25 of 28 studies recruited being of European ancestry, 97% of total sample size, we did not identify additional loci in our multi-ancestry meta-analysis including all cohorts (Supplementary Table 7; Supplementary Figures 7-10). All 11 loci identified in the European only analyses remained genome wide significant (P < 5x10^-8^) (Supplementary Figures 7-10). Further, no loci were identified at genome wide significance in ancestry-specific analyses of cohorts of Hispanic American or East Asian ancestry for IFC or ISI. Therefore, to increase power, we conducted a random effects meta-analysis of studies of Hispanic American and East Asian ancestry (Supplementary Figures 11-14; Supplementary Table 6). This identified *BICC1* (rs60453193, beta = 0.43, SE = 0.08, P = 4.06x10^-8^, N = 1,837) as a signal associated with IFC (see **Methods**; Supplementary Note, Supplementary Table 7; Extended Data 6 and 7). This locus has not been implicated in post-challenge insulin resistance previously and no significant association was seen in cohorts of European ancestry (beta = -0.0026, SE = 0.01, p = 0.80, N = 50,671), despite the lead variant being common in all three ancestry groups (Supplementary Note). Ancestry-specific loci with such effects have been identified previously for glycaemic traits, and it is possible that heterogeneous effect sizes despite similar EAF may be due to genotype-by-environment interactions or other epistatic effects (Supplementary Note).^3^ Further, the variants detected here may be in LD with ancestry specific causal variants that were not investigated in this study and differ in frequency across ancestries.^3^

### Genetic colocalisation with type 2 diabetes

We identified strong evidence of a shared genetic signal between post-challenge insulin resistance and T2D^26^ for 10 of the 11 loci identified through meta-analysis of GWAS from studies of European ancestry, using statistical colocalisation methods (posterior probability of colocalisation > 0.7; Supplementary Table 10), providing strong evidence of a shared genetic signal underlying both post-challenge insulin resistance and T2D at each of these loci. Of the 11 loci, 8 loci were associated with T2D at genome wide significance and 2 loci at the suggestive significance threshold of P < 1x10^-4^ (Supplementary Table 6)^26^. Even though the *SLC2A4* lead variant (rs117643180) was associated with an increased risk of T2D at nominal significance (beta = 0.046, SE = 0.015, P = 0.0018, N = 1,041,200, OR = 1.047) ^26^, this was the only post-challenge insulin resistance locus identified in European ancestry cohorts without evidence of a shared signal with T2D using formal colocalisation testing. rs117643180 is in perfect linkage disequilibrium (LD; D’ = 1) with lead SNPs at T2D loci identified in East Asian

and Hispanic American ancestries. Several reasons may contribute to lack of colocalisation for example differences in the allele frequency and ancestry composition considered in the respective GWAS efforts (discussed further in Supplementary Note, Supplementary Figures 15 and 16, Supplementary Table 11). Further, it is possible that rs117643180 is the causal variant underlying differences in post-challenge insulin resistance but is not sufficient to cause T2D unless accompanied by beta cell dysfunction. The IFC *BICC1* locus identified in our meta-analysis of Hispanic American and East Asian ancestry cohorts also showed no evidence of a shared signal with T2D.

Using LD score regression and genetic risk score analyses, we identified significant associations between an increased genetic risk for post-challenge insulin resistance and a range of biochemical and cardiometabolic traits (Supplementary Note; Supplementary Tables 13-16). Using LDSC-SEG we further identified tissue-specific enrichment of IFC and ISI loci in regulatory regions in T2D relevant tissues (muscle, adipose, pancreas and liver) (Supplementary Note, Supplementary Tables 21-24)

### rs117643180 (*SLC2A4*) affects transcriptional regulation in muscle

**Figure 2:**
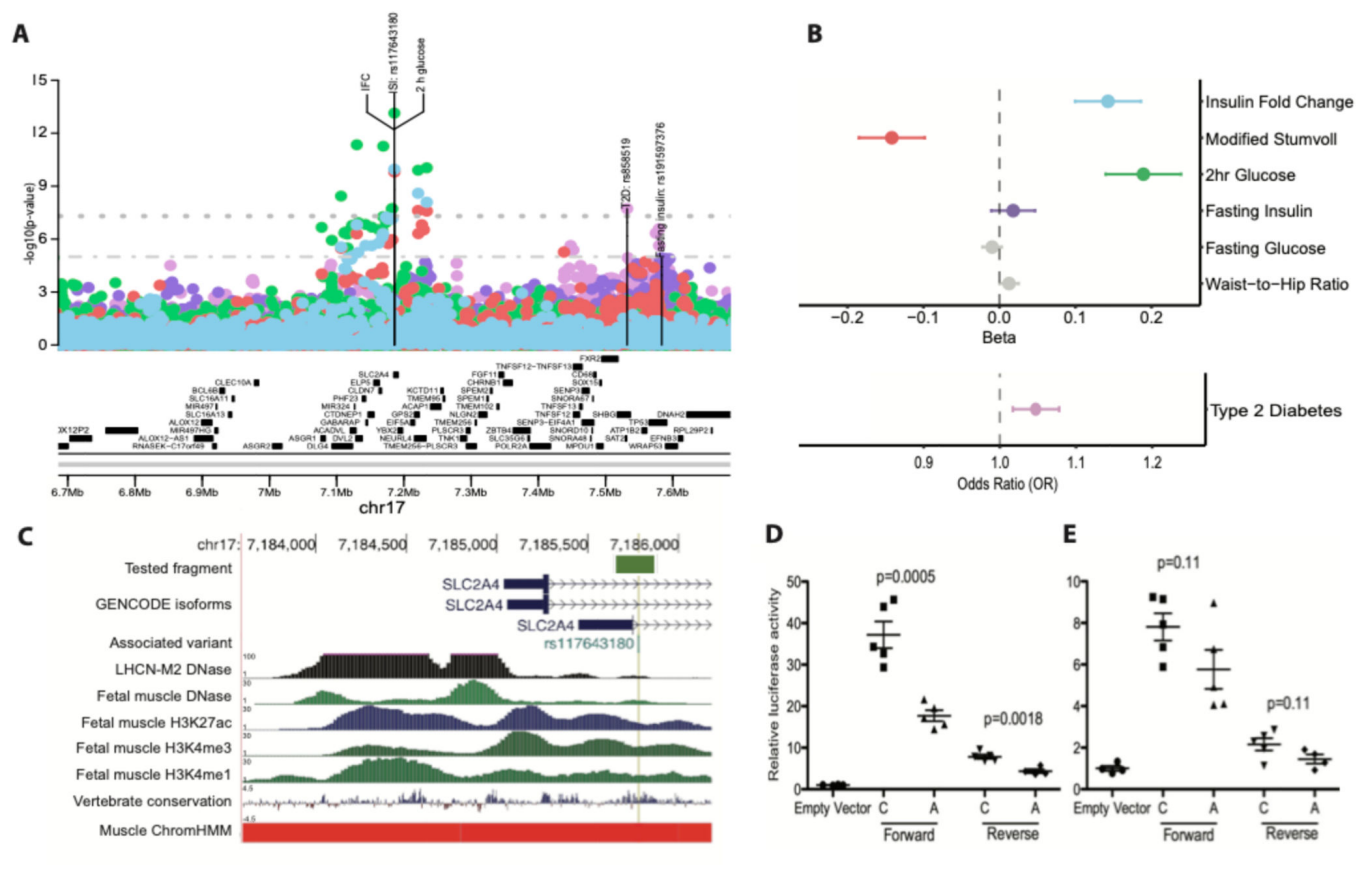
rs117643180 at *SLC2A4* affects GLUT4 expression and transcription regulation. **A**) Regional association plot showing ± 500kb around rs117643180 (SLC2A4) : insulin fold change (IFC) - blue, Modified Stumvoll (ISI) – red, and 2 h glucose^3^ – green, fasting insulin^3^ – purple, type 2 diabetes^26^ – pink. Y-axis denotes the unadjusted log10(p-value) of association. The dashed lines and dotted line indicate genome-wide (P = 5x10^-8^) and suggestive (P = 1x10^-5^) significance threshold, respectively. All association statistics are from BMI adjusted analyses in studies of European ancestry, except T2D which is unadjusted for BMI. Labels indicate the lead variant for each trait, with location indicating position. **B)** Association of rs117643180 with key metabolic traits of interest. Plot shows beta estimate or odds ratio of association for rs117643180 with traits outlined in (A) and fasting glucose^3^ and waist-to-hip ratio^53^ in grey. Point represents effect size and error bars indicate 95% CI. **C)** rs117643180 overlaps a transcriptionally active region in muscle tissues. rs117643180 is located ~730 bp downstream of the main *SLC2A4* transcription start site (TSS) and ~330 bp downstream of an alternate *SLC2A4* TSS. A 229-bp region flanking the variant, as shown by the orange bar, overlaps with accessible chromatin in LHCN-M2 (DNAase)^54^, chromatin marks of regulatory activity in fetal muscle leg tissue ^55^, a region conserved across species (vertebrate conservation), and a skeletal muscle ChromHMM^56^ (red =ActiveTSS). Created using http://genome.ucsc.edu^57^ **D-E)** rs117643180 exhibits allelic differences in transcriptional activity. Values represent fold-change of firefly luciferase/*Renilla* activity normalized to empty pGL4.23 vector in C2C12 cells, for the A (effect) and C (non-effect) alleles clones in forward and reverse orientation (See **Methods**) (D) and LHCN-M2 myotubes (E). Error bars represent the SEM of 4 to 5 independent clones tested in duplicate wells; points represent individual biological replicates. P-values are calculated from two-sided t-tests.

The only locus we identified at genome-wide significance for both ISI and IFC was *SLC2A4* (rs117643180). *SLC2A4* encodes GLUT4, the insulin-dependent glucose transporter responsible for postprandial glucose uptake into muscle and adipose tissues. rs117643180 was associated with post-challenge insulin resistance and was previously reported to be associated with higher 2 h glucose (Beta = 0.19, SE = 0.025, P = 7.31x10^-14^, N = 38,302)^3^ but not fasting insulin (P = 0.22)^3^ or fasting glucose (P = 0.14)^3^ suggesting the effect of this locus is specific to the postprandial state (Figure 2). This is, to our knowledge, the first time a post-challenge insulin resistance trait association has been reported at this locus.

rs117643180 is additionally in a region of accessible muscle chromatin since it overlaps with enhancer or promoter annotations in skeletal muscle, and adipose tissues (Figure 2c) and lies within a region of high conservation within intron 1 of *SLC2A4*. Together, these features suggest that rs117643180 is in a region of importance for *SLC2A4* gene regulation. In humans, *SLC2A4* expression is high in skeletal muscle relative to other tissues.^27,28^ rs117643180 is the lead SNP at a primary expression quantitative trait locus (eQTL) for *SLC2A4* expression in skeletal muscle, with the effect allele (A) associated with lower expression of *SLC2A4* mRNA (GTEx v8, European ancestry samples (EUR); NES (normalised effect size) = -0.50, SE = 0.073, P = 2.51x10^-11^).^29^

These data suggested altered GLUT4 levels in muscle tissue may underlie the associations of rs117643180 with post-challenge insulin resistance and its post-challenge specific glycaemic effect is due to impact on expression of *SLC2A4* mRNA ^27,30^.

We identified perfect colocalisation of the *SLC2A4* eQTL signal at rs117643180 in skeletal muscle with both IFC and ISI when conditioning on a secondary eQTL signal (rs222849) at this locus (posterior probability of colocalisation IFC/eQTL = 1.00, posterior probability of colocalisation ISI/eQTL = 1.00; Supplementary Table 17, Supplementary Figure 17). This colocalisation is specific to the primary eQTL signal for *SLC2A4* at rs117643180, the same genetic signal associated with IFC and ISI (Supplementary Table 17).

To determine whether rs117643180 exhibits allelic differences in transcriptional activity, we performed transcriptional reporter assays in murine C2C12 and human LHCN-M2 muscle cell lines using 229-bp fragments in forward and reverse orientations with respect to a minimal promoter. In all cell types, the fragment showed higher transcriptional activity than an empty vector control, especially in the forward orientation, consistent with a role as a promoter or enhancer (Figure 2D-E, Supplementary Figure 18). In mouse C2C12 myoblasts, the rs117643180-A effect allele showed half the transcriptional activity as the reference C allele in both orientations (P < 0.002) (Figure 2D). Similar results were observed in human LHCN-M2 myoblasts and myotubes (Figure 2E, Supplementary Figure 18). The lower transcriptional activity observed for the rs117643180-A allele is consistent with the GTEx v8 eQTL analysis of *SLC2A4* in skeletal muscle tissue.^29^ In addition, we carried out electrophoretic mobility shift assays using LHCN-M2 nuclear extract and probes spanning the rs117643180 alleles. These assays provided supporting evidence for allelic differences in transcription factor binding (Extended Data Figure 8). Taken together, these data suggest that rs117643180 may contribute to allele-specific differences in *SLC2A4* transcriptional activity in skeletal muscle.

### Genes implicated in insulin-stimulated glucose transport

IFC was identified to be associated with 4 loci at genome wide significance in our meta-analysis of studies of European ancestry. This included the *SLC2A4* locus directly implicating the encoded protein GLUT4. IFC further appears more specific to the post-challenge insulin resistance observationally, with IFC being more strongly correlated than ISI with 2 h measures of insulin and glucose in the Fenland Study (Supplementary Table 2, Extended Data Figure 2). Therefore, we reasoned that further study of genes at genetic loci associated with IFC, may identify additional novel genes involved in insulin-stimulated glucose disposal.

Since no such systematic follow up of genes at genetic loci associated with post-challenge insulin resistance has been reported previously, we used an inclusive approach to prioritise genes for screening their effects on GLUT4 and glucose transport in cultured cells (Extended Data Figure 1). In addition to selecting 12 genes at the 4 genome-wide IFC loci, we prioritised a further 24 genes at 4 ‘subthreshold’ loci at 5x10^-8^ < P < 5x10^-5^) that displayed a clear post-challenge specific insulin resistance association signature (See **methods**; Supplementary Note; Supplementary Table 19 and 20, Supplementary Figure 19 and 20). A total of 36 genes were selected following this strategy and we assessed the impact of siRNA gene knockdown on insulin-stimulated GLUT4 translocation in two 3T3-L1 adipocyte cell lines (Methods, Supplementary Figure 21 and 22, Supplementary Table 26).

This approach identified 4 of the 36 genes-of-interest already implicated in insulin signalling and GLUT4 trafficking significantly altered cell surface GLUT4 abundance (FDR < 5%): *Irs1*^31^, *Lnpep*^32^, *Tnks*^33,34^ and *Vps13c*^35^, as well as GLUT4 itself *(Slc2a4)* (Supplementary Tables 28 and 29). We identified a further 9 genes (*Alkbh5, Cmpk1, Drg2, Flii, Llgl1, Pdzk1ip1, Slc36a4, Toml12, Tvp23b*) whose siRNA knockdown significantly (FDR < 5%) altered absolute cell surface GLUT4 abundance and/or the ratio of cell surface GLUT4-to-total GLUT4 protein upon insulin stimulation, indicative of dysfunctional insulin-stimulated GLUT4 translocation (Supplementary Tables 28 and 29). To our knowledge, these genes have not been previously implicated in insulin action or GLUT4 trafficking and warrant further mechanistic investigation. The 14 genes identified to be implicated in GLUT4 trafficking are located across 8 IFC loci. Three of these genes were located at genome-wide loci and 11 from prioritised subthreshold loci. (Supplementary Table 30).

**Figure 3:**
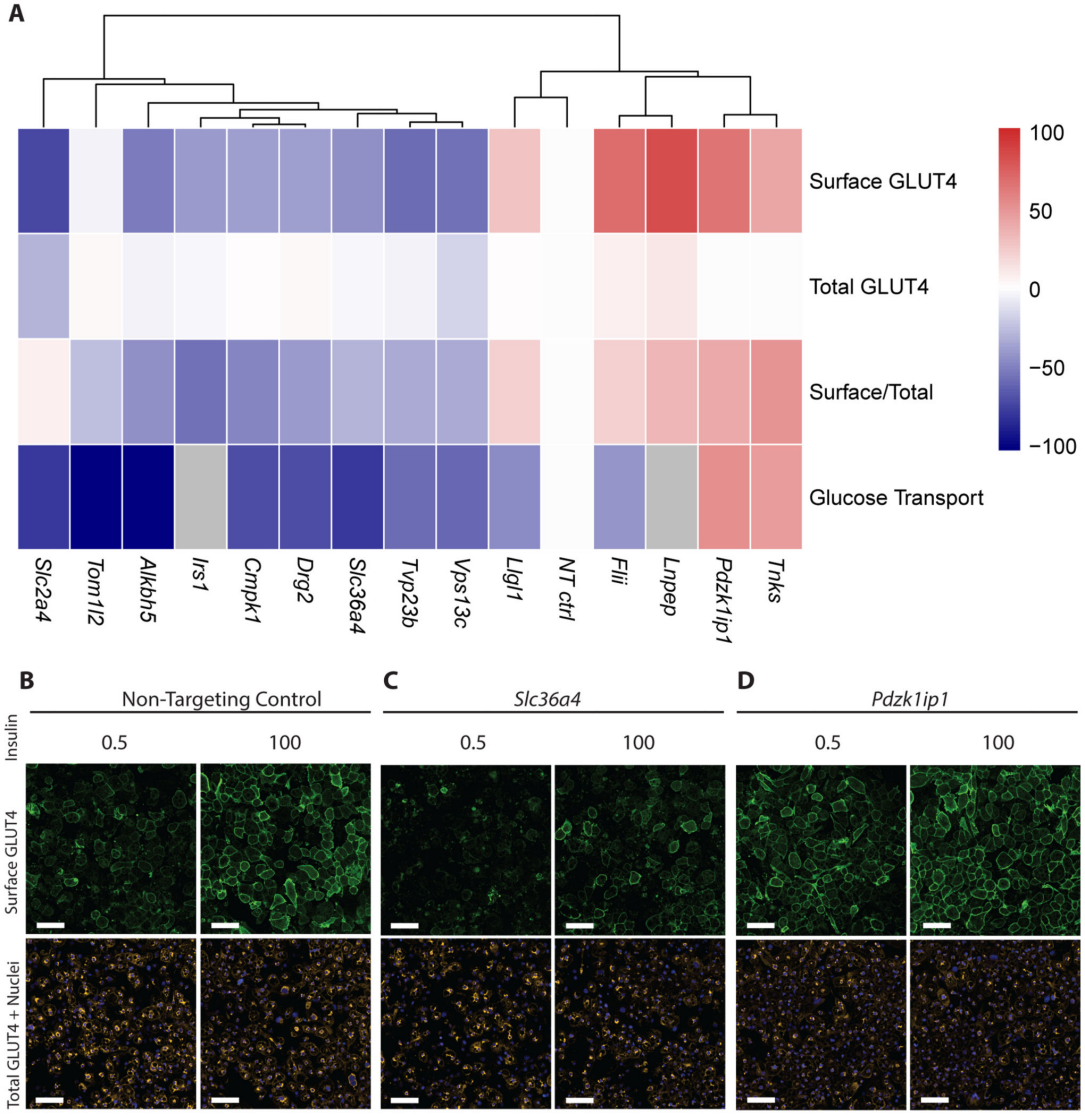
15 genes at loci associated with insulin fold change were identified to significantly impact GLUT4 trafficking in adipocytes a) Effect of gene knockdown on GLUT4 trafficking and glucose transport at 0.5 nM insulin stimulation in WT 3T3-L1 adipocytes (N = 3 biologically independent replicates, 2 technical replicates per N). Genes are clustered using hierarchical clustering. All results are normalised to non-targeting (NT) control. Grey represents genes for which glucose transport data was not collected as their role is established in the literature. Scale represents relative effect of gene knockdown compared to NT control for given measures – red positive, and blue negative, with the maximal scale representing the maximum absolute response relative to NT (0) across all measures, expressed as a percentage, to allow direct comparison across measures.-. B-D) Representative fluorescence microscopy images of example siRNA knockdown in WT 3T3-L1 adipocytes (N = 3 biologically independent replicates, 2 technical replicates per N) at 0.5 and 100nM insulin stimulation. Green represents the surface GLUT4 signal, orange the total GLUT4 signal and blue indicates individual nuclei. Scale bars indicate 100 μm. Quantification of *Slc36a4* and *Pdzk1ip1* compared to NT control and images at 0 nM are shown in Supplementary Figure 25. b) shows non-targeting control, c) Slc36a4 and d) Pdzk1ip1.

siRNA knockdown of 7 of the 9 novel genes also altered insulin-stimulated 2-deoxyglucose (2DOG) uptake in a directionally consistent manner to their observed effects on cell surface GLUT4 (Supplementary Figure 21 and 24; Supplementary Table 33). For example, knockdown of *Alkbh5, Cmpk1, Drg2, Ppp1r11, Slc36a4, Tom1l2,* and *Tvp23b* decreased cell surface GLUT4 and 2DOG uptake (Figure 3a, c) Conversely, *Pdzk1ip1* knockdown increased cell surface GLUT4 and 2DOG uptake (Figure 3a, d). Only *Flii* and *Llgl1* knockdown resulted in inconsistent effects on cell surface GLUT4 and 2DOG uptake, with increased cell surface GLUT4 but lower 2DOG uptake (Figure 3a).

## Discussion

The impairment of insulin’s ability to control glucose metabolism, or insulin resistance, is a major contributor to a range of metabolic and endocrine disorders including T2D. Previous studies of the genetic determinants of insulin resistance in humans without diabetes have predominantly used fasting samples of glucose and insulin, levels of which are largely determined by hepatic insulin sensitivity. Here, we used measures that reflect, at least in part, insulin’s important postprandial action on glucose uptake into muscle and adipose tissue. These traits act as valuable proxies of postprandial insulin resistance which are more readily scalable to population-based genetic studies than gold standard measures. Using this approach, we identified 9 loci not previously associated with post-challenge insulin resistance in studies of European ancestry, including *SLC2A4*, which encodes the glucose transporter implicated in insulin-stimulated glucose disposal. We further identified *BICC1* associated with IFC in a meta-analysis of cohorts of Hispanic American and East Asian ancestries.

The glucose transporter GLUT4 (encoded by *SLC2A4*) is enriched in muscle and fat but not present in liver. In response to insulin (and exercise in skeletal muscle), GLUT4 rapidly translocates from an intracellular pool to the cell surface to facilitate glucose uptake.^36^ Genetic ablation of GLUT4 in mice results in severe postprandial insulin resistance, hyperinsulinemia, and hyperglycaemia.^37^ Our results demonstrate that genetic variation directly influencing *SLC2A4* expression has a significant impact on human postprandial insulin action.

We also identified loci near genes directly implicated in insulin signalling including *IRS1 (reviewed in*^38^*)* and others near genes known to be involved more broadly in insulin sensitivity including *PPARG*^39^*, PPP1R3B*^40,41^, and *MTOR*^42^. The observation that 10 of the 11 loci we identified in European ancestry populations are additionally associated with increased T2D risk highlights the importance of insulin resistance in T2D pathogenesis and provides evidence of potential mechanisms behind the T2D association at these loci.

Notably, we identified genes such as *MTNR1B* and *C2CD4A* that have been implicated in T2D through an impact on pancreatic beta-cell function.^43,44^ As we used indirect measures of post-challenge insulin resistance that include measurements of circulating insulin, it is not surprising that some genetic variants regulating insulin secretion are also captured by our approach. However, at the *MTNR1B* locus, we identified a neighbouring gene *SLC36A4*, encoding a non-ionic amino acid transporter, the depletion of which resulted in a marked impairment of GLUT4 trafficking in 3T3-L1 adipocytes. Further investigations are warranted to identify possible additional effects of this locus on insulin resistance, possibly via impact on *SLC36A4*, as well as insulin secretion through *MTNR1B*.

The *BICC1* locus was the only locus associated with post-challenge insulin resistance at genome wide significance in cohorts of non-European ancestry, despite similar frequency of the lead variant in all 3 ancestry groups. We hypothesise that the identified lead variant may not be the causal variant at this locus and differences in frequency of the causal variant across ancestries may underlie the observed differences in associations.

Using technology that incorporated two complementary methods of detecting GLUT4 at the cell surface (see **Methods**), as well as direct measures of glucose uptake, we examined the impact of reducing the expression of 36 genes identified at genetic loci associated with IFC. We revealed 9 novel candidate genes implicated in insulin-stimulated glucose uptake and GLUT4 translocation from 4 IFC-associated loci. Although muscle is the major site of insulin-stimulated glucose uptake *in vivo,* the molecular mechanisms of insulin-stimulated glucose uptake are largely shared with adipocytes.^45^ To date, no skeletal muscle-based model to assess GLUT4 trafficking with insulin-responsiveness comparable to that of the mouse 3T3-L1 adipocyte has been developed.

Of the genes found to influence insulin stimulated glucose uptake, most did not affect total GLUT4 cellular content but are likely to be involved in its trafficking. These include *Llgl1*, (known to interact with Rab10^46^) and genes involved in the control of membrane traffic via the Golgi (*Drg2*^47,48^*, Tvp23b*^49^) and endosomes (*Pdzk1ip1*^50^*, Toml12*^51^), both key sites of GLUT4 traffic. In contrast interference with the expression of Alkbh5, a M6A RNA demethylase, which has previously been implicated in post-transcriptional regulation of GLUT4 expression in a breast cancer model^52^, also reduced total GLUT4 cellular content in our studies. Additional processes involved in the maintenance of the cellular GLUT4 pool, including transcription, stable intracellular localisation, or protein degradation may be involved in the case of other genes.

To conclude, here we report a total of 10 genome-wide loci not previously associated with post-challenge insulin resistance. We show that 8 of these share their genetic architecture with T2D and hence identify the likely mechanism of action that leads to T2D pathogenesis for these loci, and identify one locus, *BICC1*, to be specific to cohorts of Hispanic American and East Asian ancestries, despite much smaller sample size.

We identify a common intronic variant (rs117643180) in *SLC2A4* to be associated with post-challenge insulin resistance and demonstrate it’s *in silico* and *in vitro* effect on *SLC2A4* expression in skeletal muscle. *SLC2A4* encodes GLUT4 - a critical player in postprandial glucose disposal, and together this provides evidence for *the* mechanism underlying the post-challenge specific glycaemic signature seen for *SLC2A4* in individuals without diabetes. Finally, through the employment of cell-based screening methods, we identified 9 putative novel genes involved in GLUT4 trafficking. This study highlights the importance of characterising refined dynamic measures of insulin resistance to further the understanding of physiological mechanisms in T2D that are not sufficiently captured by more readily obtainable measures at a single timepoint.

## Materials and Methods

### Contributing studies

All contributing studies included in this analysis were approved by the relevant Institutional Review Boards, with all participants having given informed consent. Study specific ethics statements are provided in the **Reporting Summary**.

A total of 55,535 and 55,172 participants without diabetes across 28 studies contributing to the Meta-Analyses of Glucose and Insulin-related Traits Consortium (MAGIC) contributed to the analyses of Modified Stumvoll ISI and insulin fold change, respectively (Supplementary Tables 1-3). Two of these studies included participants of Hispanic American ancestry (MACAD and HTN-IR; N ISI = 1095, N IFC = 1099) and 1 included participants of East Asian ancestry (TAICHI; N ISI = 730 N IFC = 739); the remaining 25 studies included individuals of European ancestry.

Ancestry group was defined at the study level as the ancestry reported by each respective study at point of data collection. Within each study, we excluded individuals with missing traits or covariates from the analyses.

### Phenotype definition

Individuals were excluded from study-level analyses if they were diagnosed with type 2 diabetes, reported use of diabetes medication, or had Fasting Glucose ≥ 7mmol/L, 2hr Glucose ≥ 11.1 mmol/L or HbA1c ≥ 6.5% (Supplementary Table 1).

Fasting measures of plasma insulin and glucose were collected before a standard 75g oral glucose tolerance test (OGTT) with 2hr glucose and insulin measures obtained 120mins after the ingestion of the glucose challenge. Biochemical assays used to quantify insulin and glucose in individual studies are outlined in Supplementary Table 1.

Modified Stumvoll insulin sensitivity index (ISI) was calculated as previously described using the formula^22,58^:

$$\begin{array}{l} \;Modified\;Stumvoll\;ISI\;\\ \;=0.156-(0.0000456\times \;insulin{\;}_{120\text{min}[\text{pmol}/L]})\\ \;-(0.000321\times \;insulin{\;}_{0\text{min}[\text{pmol}/L]})\\ \;-(0.0054\times glu\text{cos}e_{120\;\text{min}[\text{mmol}/L]}) \end{array}$$

Insulin fold change (IFC) was calculated using fasting and 120 min insulin measures during OGTT using formula:

$$IFC=\text{ln}\left(\frac{{\text{Insulin}\;}_{120\text{mins}[\text{pmol}/\text{L}]}}{{\text{Insulin}\;}_{0\text{mins}[\text{pmol}/\text{L}]}}\right)$$

Formulae terms: insulin_120min_ – insulin levels at 120 mins post-ingestion of glucose challenge in pmol/L. Insulin_0min_ – fasting insulin levels prior to glucose challenge in pmol/L. Glucose_120min_ – glucose levels at 120 mins post-ingestion of glucose challenge in mmol/L.

### Phenotypic correlations of ISI and IFC with relevant traits

We assessed observational correlations of relevant traits was assessed in the Fenland^59^ and RISC^60^ studies using Spearman's Rank correlation for pairwise complete observations. ISI and IFC were calculated as defined above and were restricted to individuals without diabetes. Those who reported use of diabetes medication or had fasting glucose ≥ 7 mmol/L, 2hr glucose ≥ 11.1 mmol/L or HbA1c ≥ 6.5% were excluded. Blood biochemistry measures were natural log transformed. In RISC and the Fenland Study, IFC was calculated using OGTT measures of insulin at 0 min and 120 min.

### Genotyping, quality control, (QC), and imputation

Study-level QC, genotype imputation, and genome-wide association study (GWAS) analyses were conducted following a shared analysis plan to ensure analyses were conducted consistently across studies. Most study samples were genotyped using commercially available genome-wide genotyping arrays, with a small subset of study samples genotyped on targeted arrays such as Illumina Cardio-Metabochip (Metabochip) array (Supplementary Table 1).

Genotype imputation was conducted for all autosomes and the X chromosome in all contributing studies on build 37 (Hg19) on the forward strand. Most studies of European ancestry were imputed to the Human Reference Consortium (HRC) reference panel.^61^ Studies of non-European ancestry were imputed to population-specific 1000 genomes (1000G) reference panel^62^. Botnia-PPP was imputed to the SISu v3 reference panel in genome build 38^63^ and variants were mapped to build 37 using UCSC LiftOver, after GWAS analyses^64^.

### Study-level GWAS

Study-level GWAS were performed using an additive model, modelling on the inverse normal transformed residuals of each trait and adjusting for age, sex, population structure, and study-specific covariates with or without adjustment for BMI. (Supplementary Table 1). To control for type 1 error rate of low-frequency variants and remove trait-covariate correlations, covariate adjustment for each trait was conducted in two steps before association analysis. First, a given trait was adjusted for all covariates in a model and residuals were derived. These residuals were inverse normal transformed and then adjusted for all covariates.

### Study-level GWAS QC

Each contributing study shared GWAS summary statistics for both traits and both models. Study-level GWAS QC was performed by the central analysis group using easyQC (v17.8), to assess trait transformation and exclude variants that failed QC from downstream analyses.^65^ Variants were flagged if the differences of their minor allele frequency (MAF) between the study and the corresponding frequencies in the imputation reference panel were greater than 0.2. In addition, variants were excluded if they met one or more of the following criteria: monomorphic, low genotype call rate (< 95%), poor imputation quality (INFO < 0.3), duplicate variants, minor allele count (MAC) < 3 per study, or not in Hardy Weinberg Equilibrium (HWE P < 1x10^-5^). Variants were additionally excluded if these had standard error of the effect size > 10 or were missing standard error, effect estimate, or p-value in the summary statistic file. For the RISC study variants were excluded where INFO < 0.8.

### Single-ancestry meta-analysis

An inverse variance fixed-effect meta-analysis of the study level GWAS was done using METAL (v.2011-03-25) for each ancestry, using beta-coefficients and standard errors from each study-level GWAS.^66^ We applied genomic control correction in two stages, first to study-specific GWAS results and then to the meta-analysis. Study-specific Metabochip results were corrected by genomic control using test statistics for 4986 SNPs included on the Metabochip array associated with QT interval, a trait that is not correlated with glycaemic traits, consistent with previous analyses of other glycaemic traits.^3,67^

In each individual study variants from with a MAF < 0.005 were removed prior to meta-analysis. Genome-wide significance was considered at P < 5x10^-8^, with a genomic locus considered as a 1 Mb window (± 500kb) around the lead variant at each locus. Loci were annotated with the nearest protein-coding gene where applicable or based on previously reported gene annotation in the literature. Lead variant associations were manually inspected across all individual studies to ensure consistency of association.

Conditional analysis was conducted using the –cojo-slct option in GCTA (v1.26.0) using BMI adjusted summary statistics for IFC and ISI in European studies to identify independent signals at each locus^68^. A collinearity threshold of 0.8 and conditional p-value threshold of P < 5x10^-8^ were used to identify conditionally independent genetic signals at each locus. The Fenland-OMICS subset (N = 8,925) of the Fenland Study with dense genome wide genotyping was used as an LD reference panel.

### Multi-ancestry meta-analysis

We conducted a multi-ancestry random-effects meta-analysis using random-metal (2017-07-24; https://github.com/explodecomputer/random-metal) to meta-analyse the European, Hispanic American (His-Amr) and East Asian (EAS) ancestry specific analyses (see above), to delineate any ancestry specific effects on these traits.^66,69^ The meta-analysis strategy is summarised in Extended Data Figure 4.

### Post meta-analysis QC

Post-meta-analysis, variants with a total sample size less than 11,000 in the European ancestry only or multi-ancestry (EUR, HIS-AMR, and EAS) analyses were removed, ensuring that the associations observed were contributed to by at least 2 studies. In the non-European ancestry (HIS-AMR and EAS) analysis, variants detected in < 1,000 individuals were removed, ensuring these associations were seen in at least 2 of the 3 studies.

Novel loci were defined as those loci which have not been reported to be associated with post-challenge glycaemic traits or indices reflecting post-challenge insulin resistance.

For both IFC and ISI, fixed effect meta-analyses of GWAS from BMI adjusted (adjBMI) analyses in cohorts of European ancestry were considered the primary analyses. Unless specified otherwise, the results of this primary analysis are reported.

### Fine mapping

We performed fine-mapping of the 11 loci significantly associated with IFC or ISI in European ancestry meta-analysis to define 99% credible sets for each locus. We generated approximate Baye’s factors (ABFs) using the method described by Wakefield (2009)^70^ and calculated the posterior probability that each variant was causal. Credible sets were defined including all variants that contained cumulative posterior probability greater than 99% in each region. Credible sets were calculated using marginal summary statistics for all loci where there was identified to only be one independent signal. At *PPP1R3B* where 2 independent signals were identified for insulin fold change, conditional summary statistics were used to calculate the 99% credible set for each signal.

### Statistical colocalization of IFC and ISI with T2D

Bayesian colocalisation analyses were conducted using coloc.abf from R package coloc(v 5.1.0)^71^ to test for genetic colocalisation of IFC and ISI with T2D^26^. BMI adjusted summary statistics in studies of European ancestry were used except for T2D where BMI adjusted stats were not available. Colocalisation was considered at a threshold of posterior probability > 0.7 for hypothesis “PP.H4.abf”: a locus is associated with both traits and there is one shared causal SNP for the pair of traits being assessed.

### Phenotypic annotation of associated loci

We used gene expression data from the GTEX project to assess whether lead variants are annotated as tissue specific expression quantitative trait loci (eQTLs) at a suggestive significance threshold of P < 1x10^-4^ in tissues relevant to type 2 diabetes – skeletal muscle, adipose (subcutaneous and visceral), liver, kidney^29^. We additionally used summary statistics for relevant traits that are publicly available to further annotate the loci of interest: T2D (N control = 965,732, N case = 148,726)^26^, Waist-to-hip ratio (WHR) (N = 694,649)^53^, fasting insulin (N=151,000)^3^, fasting glucose (N = 200,600)^3^, 2hr glucose (N = 63,400)^3^. BMI adjusted summary statistics in studies of European ancestry were used for all traits except T2D where BMI adjusted statistics were not available. These were additionally used to annotate lead variants at loci identified in the integrated GWAS approach (see below). Rare variant burden association statistics were obtained from the AstraZeneca PheWAS Portal (https://azphewas.com/) which includes pheWAS analysis across exomes from ~ 450,000 individuals of European ancestry in UK Bioabank. We extracted statistics for rare damaging missense variants which were defined using the “raredmgmtr” variant mask – which includes high quality calls for missense variants that are rare (MAF < 0.005%), damaging (REVEL score >= 0.25), and fall into a region of constrained variation according to the missense tolerance score metric (MTR < 0.78 or MTR_centil < 0.5). For more information, please see the publication outlining this work.^72^

### Genetic risk scores and LD Score regression

Genetic risk scores (GRS) for IFC and ISI, and LD score regression^73^ were used to assess the association of IFC and ISI with other cardiometabolic traits. We constructed genetic risk scores (GRS) for IFC and ISI including lead variants at primary association signals in Europeans, weighted by their effects on IFC or ISI as appropriate, both adjusted and unadjusted for BMI (Supplementary Note, Supplementary Table 12). LD scores for ISI and IFC were calculated using BMI adjusted summary statistics from our analyses in cohorts of European ancestry. LDSC^73^ was used to assess the genome wide correlation with other cardiometabolic traits. LDSC-SEG was additionally used to assess the genome-wide enrichment of tissue-specific and cell type specific annotations.^74^ Further details can be found in the Supplementary Note.

### Statistical colocalisation with relevant traits

We further assessed colocalisation of traits using hyprcoloc(v1.0)^75^ to identify shared signals across the following traits: T2D^26^, WHR, fasting insulin^3^, 2hr glucose^3^, ISI, and IFC. BMI adjusted summary statistics in studies of European ancestry, expect T2D which was unadjusted for BMI. Loci were defined as ±500kb around the lead variant for IFC and ISI, at a significance threshold of p < 1 x10^-5^. Additional traits assessed were dropped from the analysis at each of the selected loci where P > 5x10^-4^. Hyprcoloc was run across default priors (p1 = 1x10^-4^, p2 = 0.98 and threshold = 0.5) as well as looped over a grid of priors to allow for optimisation (p1 = 1x10^-4^; p2 = 0.95, 0.98, 0.99, 0.999; threshold = 0.5,0.6, 0.7, 0.8, 0.9). Colocalisation of traits was considered at a threshold of posterior probability of colocalisation > 0.8, the largest and most stable trait cluster using the most the most stringent set of priors at this threshold was selected. The stability of trait clusters was inspected using sensitivity plots, and pairwise colocalisation between traits at these loci, also using hyprcoloc.

### Statistical colocalisation with eQTLs in relevant issues

We additionally tested for colocalisation of eQTLs in relevant tissues (skeletal muscle, adipose, liver, and pancreatic islet) with IFC/ISI at the loci of interest. We considered co-localisation significant when the colocalisation posterior probability was > 0.7. We used eQTL marginal summary statistics in individuals of European ancestry from the GTEx consortium (v8) in relevant tissues (Adipose – Subcutaneous, Adipose – Visceral Omentum, Muscle – Skeletal, Pancreas, Liver).^27,30^ Bayesian colocalisation analyses were conducted using coloc.abf from R package coloc using default priors.^71^

### *In vitro* assessment of rs117643180 at SLC2A4

#### Cell culture

C2C12 murine myoblasts (TCF-UNC/ATCC; CRL 1772) were grown in high-glucose Dulbecco’s Modified Eagle’s Medium (Sigma-Aldrich, St. Louis, MO) supplemented with 10% fetal bovine serum. LHCN-M2 immortalized human myoblasts^76^ (Purchased from Evercyte, Vienna, AT) were grown on dishes coated with 0.1% pigskin gelatin (Sigma-Aldrich) in a medium consisting of four parts 4.5 g/L DMEM and one-part M199 (both Gibco, Waltham MA) and supplemented with 15% FBS, 20 mM HEPES buffer, 30 ng/mL zinc sulfate, 1.4 ug/mL vitamin B12, 55 ng/mL dexamethasone, 2.5 ng/mL HFG, and 10 ng/mL basic FGF. To differentiate LHCN-M2 myoblasts to myotubes, cells were cultured on gelatin-coated dishes for seven days in 4:1 DMEM: M199 supplemented with 20 mM HEPES buffer, 30 ng/mL zinc sulfate, 1.4 ug/mL vitamin B12, 10 ug/mL insulin, and 100 ug/mL apotransferrin. All cells were maintained in a humidified incubator at 37°C with 5% CO2. Cells tested negative for *Mycoplasma* contamination in accordance with the MycoAlert Mycoplasma Detection Kit (Lonza, Morristown, NJ).

#### Transcriptional reporter assays

Dual-luciferase reporter assays were performed as previously described^77^. A 229-base pair fragment surrounding rs117643180 (chr17:7185651-7185879, GRCh37/hg19) was amplified via PCR and cloned into the firefly luciferase reporter vector pGL4.23 (Promega, Madison, WI) in both orientations with respect to the minimal promoter. This fragment is located in *SLC2A4* intron 1 and includes approximately 100 bp of an untranslated alternate first exon. Alleles for additional variants located within the insert were kept consistent across all plasmids. To create plasmids containing the rs117643180-A allele, we performed site-directed mutagenesis using QuikChange II (Agilent Technologies, Santa Clara, CA) and PAGE-purified primers (Integrated DNA Technologies, Newark, NJ). Four or five clones for each allele, in each orientation, were purified and sequence confirmed (GeneWiz, RTP, NC). Primer sequences can be found in Supplementary Table 18.

Duplicate wells of C2C12 cells (35,000 per 1.9 cm^2^) were co-transfected with 500 ng of luciferase plasmid and 80 ng of phRL-TK *Renilla* luciferase reporter plasmid (Promega) using transfection reagent Lipofectamine 3000 (Thermo Fisher, Waltham, MA). Duplicate wells of LHCN-M2 cells (40,000 per 1.9 cm^2^) were co-transfected similarly either in an undifferentiated state or on day 6 of differentiation using transfection reagent LTX (Thermo Fisher). Reporter assays were performed 30 hours (LHCN-M2) or 48 hours (C2C12) after transfection. Luciferase measures were normalized to corresponding *Renilla* measures, and mean fold-change across duplicate wells was calculated relative to two to four independent preparations of empty vector pGL4.23. Alleles were compared using two-sided unpaired t-tests (α = 0.05). All transfections were repeated on a different day and showed consistent results.

#### Electrophoretic mobility shift assays

Assays were conducted as previously described using C2C12 or LHCN-M2 nuclear extracts.^78^ Seventeen-bp complementary oligonucleotides centred around rs117643180-C or rs117643180-A and biotinylated at the 5’ end (IDT) were annealed (Supplementary Table 18).. Binding reactions were carried out with 6-10 μg of nuclear extract, 200-400 fmol of biotinylated probe, and 10- to 30-fold excess of unlabelled competing probe.

#### Integrated GWAS approach

We employed an integrated GWAS approach, which has been successful in identifying variants associated with insulin resistance^79^, to identify genetic variants which had a similar association pattern to that of the *SLC2A4* locus, using a more permissive significance threshold. We filtered for variants which were associated with IFC and ISI (P < 5x10^-5^). Those prioritised for IFC were used as a starting point for gene selection for in vitro follow up work assessing the role of candidate genes in GLUT4 trafficking (see below).

We subsequently integrated associations for IFC and ISI with the associations reported for fasting insulin^3^, 2hr glucose^3^, and skeletal muscle eQTLs^29^. We used BMI adjusted summary statistics from meta-analyses including studies of European ancestry for all traits. For IFC and Modified Stumvoll ISI, we used the European only BMI adjusted analyses. For 2hr glucose and fasting insulin, we utilised the largest available BMI adjusted summary statistics in European studies.^3,5^ Lead variants for IFC or ISI at the loci selected to not be associated with fasting insulin (strategy 2 outlined below), were subsequently assessed for their association and statistical colocalisation (see below) with mRNA expression levels in skeletal muscle (eQTLs) in GTEx v8^29^.

We used two variant selection strategies to refine loci which have the following phenotypic patterns of association:

1. Identify loci additionally associated 2hr Glucose (P < 5x10^-4^)
2. Identify loci not associated with Fasting insulin (P > 0.05) and reported to be an eQTL in skeletal muscle (GTEx^29^, P < 5x10^-4^). We also annotated loci with eQTLs in skeletal muscle identified in FUSION (P < 0.05)^80^.

Where conditions were met at a given locus for both ISI and IFC, the lead variant was selected to be that that met the threshold for both ISI and IFC.

#### Assessing the role of genes at IFC loci in GLUT4 trafficking

IFC is correlated with post-challenge insulin and glucose to a greater extent than fasting measures (Supplementary Note; Extended Data Figures 2 and 3). Therefore, following our identification of the *SLC2A4* locus directly implicating the encoding protein, GLUT4, we reasoned that studying genes at genetic loci associated with IFC may allow identification of additional novel regulators of insulin-stimulated glucose disposal. Loci for IFC identified at genome-wide significance and those prioritised through the integrated GWAS approach were used for gene selection.

We prioritised all protein-coding genes defined in UCSC genome browser^81^ located ±500kb of a lead variant at a given locus. Genes were subsequently filtered based on evidence of expression in human skeletal muscle and adipose tissue (GTEx v8) – the primary tissues where insulin-stimulated GLUT4 trafficking occurs^36^. Evidence of expression was considered where transcripts per million (TPM) greater than 1 was reported at least one of these tissues for a given gene.

We further assessed the expression of these genes in differentiated 3T3-L1 adipocytes in a publicly available RNA-sequencing dataset (GEO accession: GSE129957; FPKM >1), and by qPCR (see below).^82^

36 genes at genetic loci associated with IFC were depleted using short interfering RNA (siRNA) and the effect of gene knockdown on insulin-stimulated GLUT4 translocation was assessed in 3T3-L1 adipocytes (Supplementary Figure 21, Supplementary Table 26). These experiments were conducted in 3T3-L1 adipocytes stably expressing a GLUT4 construct (HA-GLUT4-mRuby3), and complementary experiments were conducted in wild type 3T3-L1 adipocytes to ensure findings were relevant to endogenous GLUT4 (Supplementary Figure 25 and 26, Supplementary Tables 28 and 29). Using this approach, knockdown of the kinases *Akt1* and *Akt2* as well as the Rab protein *Rab10* impaired insulin-stimulated HA-GLUT4-mRuby and endogenous GLUT4 translocation, as expected. ^83,84^ *Tbc1d4* knockdown suggested an increase in cell surface GLUT4, consistent with its role as a negative regulator of GLUT4 translocation^15^ but this did not reach significance threshold of FDR < 5%. Based on testing a subset of 20 siRNAs by qPCR, including all genes reaching significance, as well as other genes where siRNA knockdown did not affect GLUT4 trafficking, the average knockdown efficiency was 85% (Supplementary Figure 23; Supplementary Table 32).

#### Cell culture

3T3-L1 preadipocytes (originally obtained from Howard Green, Harvard Medical School) were maintained in Dulbecco’s modified Eagle’s medium (DMEM) supplemented with 10% foetal bovine serum (FBS) and GlutaMAX in a humidified incubator at 37 °C with 10% CO2. For 3T3-L1 stable cell lines, preadipocytes were transduced with pBABE-puro retrovirus expressing HA-GLUT4-mRuby3. Puromycin (2 μg/mL) was used to select transduced cells. 3T3-L1 preadipocytes were seeded into 6 well plates and were differentiated into adipocytes. Differentiation was initiated at 100% confluence through addition of DMEM/FBS/GlutaMAX supplemented with 350 nM insulin, 0.5 mM 3-isobutyl-1-methylxanthine (IBMX), 250 nM dexamethasone, and 400 nM biotin. After 3 d, cells were moved to DMEM/FBS/GlutaMAX supplemented with 350 nM insulin for a further 3 d. Cells were reverse transfected with control or targeting siRNAs 6 d after the initiation of differentiation.

#### siRNA-mediated gene knockdown in differentiated adipocytes

Dharmacon siGenome siRNA pools (Horizon Discovery) to genes-of-interest (see Supplementary Table 23 for details) and *Akt1 and Akt2, Tbc1d4, Rab10* as positive controls were resuspended in RNAse-free water in a laminar flow hood in RNAse free environment following manufacturer specifications. The concentration of resuspended siRNA was measured using Nanodrop and standardized to 25 μM. Aliquots of diluted siRNA were stored at -80°C and thawed on ice prior to use.

For reverse transfection, 2.5 μL of TransIT-X2 transfection reagent and 1.25 μL of siRNA (25 μM) was added to 63 μL Opti-MEM and incubated at room temperature for 30 min. 3T3-L1 adipocytes 6 days post-differentiation were trypsinised in 10x trypsin-EDTA at 37°C, resuspended in DMEM/FBS media and centrifuged at 200 x *g* for 5 min at room temperature. Pelleted cells were resuspended in DMEM/FBS/GlutaMAX (13.5 mL per 6-well plate) and 563 μL of resuspended cells were added to each tube of transfection reagent. Media containing cells and siRNA complexes were reseeded into Matrigel-coated 96-well plates (95 μL per well). For seeding of cells into 24- or 48-well plates, the amounts of OptiMEM, transfection reagent, siRNA and cells were adjusted appropriately based on well area. After 24 h, cells were washed with DMEM/FBS/GlutaMAX and media was replaced every 24 h. Cells were assayed 96 h postknockdown.

Knockdown of positive control genes, where involvement in GLUT4 trafficking is well established, was validated at the protein level by western blot (Extended Data Figure 9). Knockdown of genes that were identified to be of interest following GLUT4 translocation assay were validated at the mRNA level by qPCR (Supplementary Figure 25). See methods below for further details.

### GLUT4 translocation assay

#### Assay design

siRNA knockdown in each experiment was performed in technical duplicates. Non-targeting (NT) siRNA was used as the control condition in all experiments, with three sets of NT control per assay plate. Positive control siRNA targeting *Akt1 and Akt2, Tbc1d4, Rab10* mRNA were included in all experiments.

#### Insulin stimulation and immunofluorescence staining

96 h after transfection cells were washed 3x in serum-free DMEM and incubated in DMEM/GlutaMAX/0.2% BSA for 2 h. Cells were stimulated with 0.5 nM or 100 nM insulin for 20 min. Cells were washed by gently immersing the 96-well plates 12 times in beakers containing ice-cold PBS (6 washes in each of two 1 L beakers containing PBS; all the subsequent PBS washes were performed using this method). Plates were placed on ice and residual PBS was removed with a multichannel pipette. Cells were fixed with 4% paraformaldehyde (PFA) for 5 min on ice, and 15 min at room temperature. PFA was quenched with 50 mM glycine in PBS for 10 min. Cells were washed in PBS and blocked with 5% Normal Swine Serum (NSS, Dako, X0901) in PBS for 20 min. Cells were then incubated with primary antibody solution in 2% NSS in PBS for 1 h at room temperature. For detecting endogenous PM GLUT4 in wild type 3T3-L1 adipocytes, cells were incubated with human anti-GLUT4 antibody (1:1000; LM048; antibody kindly provided by Joseph Rucker, Integral Molecular, PA, USA)^85^. For detecting PM HA-GLUT4-mRuby3, cells were incubated with mouse anti-HA (1:500, Covance, MMS-101R). After PBS washes, cells were incubated with secondary antibody solution in 2% NSS in PBS containing Hoechst 33342 (1:5000; Life Technologies) for 1 h at room temperature. For detecting endogenous GLUT4, we used Alexa-488-conjugted anti-human IgG antibody (1:500; Life Technologies). For HA-GLUT4-mRuby3 expressing cells, we used Alexa-488-conjugted anti-mouse IgG antibody. 1:500; (Life Technologies). Cells were washed 12 times in RT PBS and stored in PBS containing 2.5% DABCO, 10% glycerol, pH 8.5, sealed and kept at 4°C in the dark prior to imaging. Plates were equilibrated to room temperature for 30 min before imaging.

Plates were imaged on the Perkin Elmer Opera Phenix High Content Screening System and mid-section confocal images were obtained using a 20x water objective (N.A 1.0), with 2-pixel binning. Excitation wavelengths and emission filters used were as follows: endogenous surface GLUT4 and HA-tagged surface GLUT4: 488 nm, 500-550 nm; endogenous total GLUT4 and mRuby-tagged total GLUT4: 561 nm, 570-630 nm; Hoechst: 405 nm, 435-480 nm. The microscope settings were automated and pre-defined before imaging of the entire plate, meaning identical positions in each well were sampled across all wells/siRNA knockdown conditions without any human supervision. Six to eight positions in the centre of each well were selected for imaging. As knockdowns were performed in technical duplicates across two 96-well plates, for samples where 8 fields of view were captured per a well each condition represents the mean of 16 images, per biological replicate. Following acquisition, positions across each plate and well were inspected at random to ensure proper seeding/staining for quality assurance.

All targeting siRNAs were assigned numbers throughout the entire screen to preserve anonymity. The seeding order was rearranged for each biological replicate to control for any variability arising from the well position in the plate.

To quantify total endogenous GLUT4 expression, in WT 3T3-L1 adipocytes, cells were permeabilised and stained with anti-GLUT4 antibody after plates had been imaged for surface GLUT4. Cells were permeabilised in 5% NSS in PBS containing 0.1% saponin for 20 min, followed by incubation in 2% NSS in PBS containing rabbit anti-GLUT4 antibody (1:200; Courtesy of Professor David James, University of Sydney)^86^. After washing with PBS, cells were incubated with 2% NSS in PBS containing Alexa-568-conjugated goat anti-rabbit IgG antibody (1:500; Life Technologies) for 1 h at room temperature. Plates were then re-imaged on the Perkin Elmer Opera Phenix High Content Screening System as described above.

#### Imaging data analysis

Imaging data were analysed using Harmony phenoLOGIC Software (v4.9; Perkin Elmer). For endogenous GLUT4 indicated by the Alexa 488 signal was quantified for surface GLUT4 prior to permeabilisation and total GLUT4 following permeabilisation and re-staining with Alexa-568 (see above). Surface GLUT4 was quantified in HA-GLUT4-mRuby cells by Alexa-488 antibody and total GLUT4 by the mRuby signal. Nuclei were quantified using the Hoechst signal, and nuclei were defined and quantified using pre-set method A. Surface and total GLUT4 staining were quantified by taking the mean intensity across the entire field of view – the fluorescence intensity across 8 fields of view were then averaged to provide a final fluorescence intensity for each well. The intensity across two technical replicates (wells on separate 96-well plates) were then averaged providing a mean intensity for 16 fields of view, per biological replicate. The surface GLUT4 levels were subsequently normalised to nuclei number. The ratio of surface to total GLUT4 levels were calculated from the surface GLUT4 levels normalised to the total GLUT4 signal.

### Data processing and statistical analysis

Experimental measures per plate were normalised to the average signal of all conditions across each plate for a given parameter Differences in mean measures for a given knockdown were compared to the non-targeting control values using a two-sided t-test in R (v4.0.4) across all replicates (N biological endogenous GLUT4 = 3; N biological replicates HA-GLUT4-mRuby = 5, 2 technical replicates per N). Significance was considered at a false discovery rate of 5% (FDR < 5%). Candidate genes which were significantly different to control (q < 0.05) for surface/nuclei# and/or surface/total GLUT4 in at least 1 of the assayed cell lines were taken forward for Radiolabelled Glucose Transport Assays.

### Radiolabelled glucose transport assay

Following siRNA-knockdown, 3T3-L1 adipocytes were serum-starved for 2 h in DMEM containing 0.2% BSA and GlutMAX media in a humidified incubator, 37°C 10% CO_2_. Cells were washed three times in pre-warmed Krebs-Ringer buffer (KRP; 0.6 mM Na_2_HPO_4_, 0.4 mM NaH_2_PO_4_, 120 mM NaCl, 6 mM KCl, 1 mM CaCl_2_, 1.2 mM MgSO_4_ and 12.5 mM HEPES (pH 7.4)) at 37°C. Cells were stimulated with 0.5 nM insulin for 20 min where indicated. To determine the non-specific glucose uptake 25 μM cytochalasin B (Sigma Aldrich) was added to the wells before the addition of H^3^-2-deoxyglucose (2DOG). During the final 5 min of insulin stimulation, 2DOG (0.25 μCi, 50 μM unlabelled 2DOG) was added to wells to measure glucose uptake rates. Following three washes in ice-cold PBS, cells were solubilized in 1% (w/v) Triton X-100 in PBS on a shaker for 1 h. Tracer uptake was assessed by liquid scintillation counting using a β-scintillation counter. Values for all samples were normalized to protein content, as determined by bicinchoninic acid (BCA) assay (Thermo Fisher Scientific).

### Confirmation of gene knockdown

Gene knockdown by siRNA was confirmed by qPCR for a subset of 20 targets, including all genes where knockdown resulted in a significant impact on GLUT4 (Supplementary Note, Supplementary Table 31 and 32; Supplementary Figure 23). For genes already established to be implicated in GLUT4 trafficking – Irs1, Lnpep, Tbc1d4, Slc2a4 and Akt, SDS-PAGE and immunoblotting was also used to confirm knockdown at the protein level (Extended Data Figure 9). Further details can be found in the Supplementary Note.

## Extended Data

**Extended Data Figure 6:**
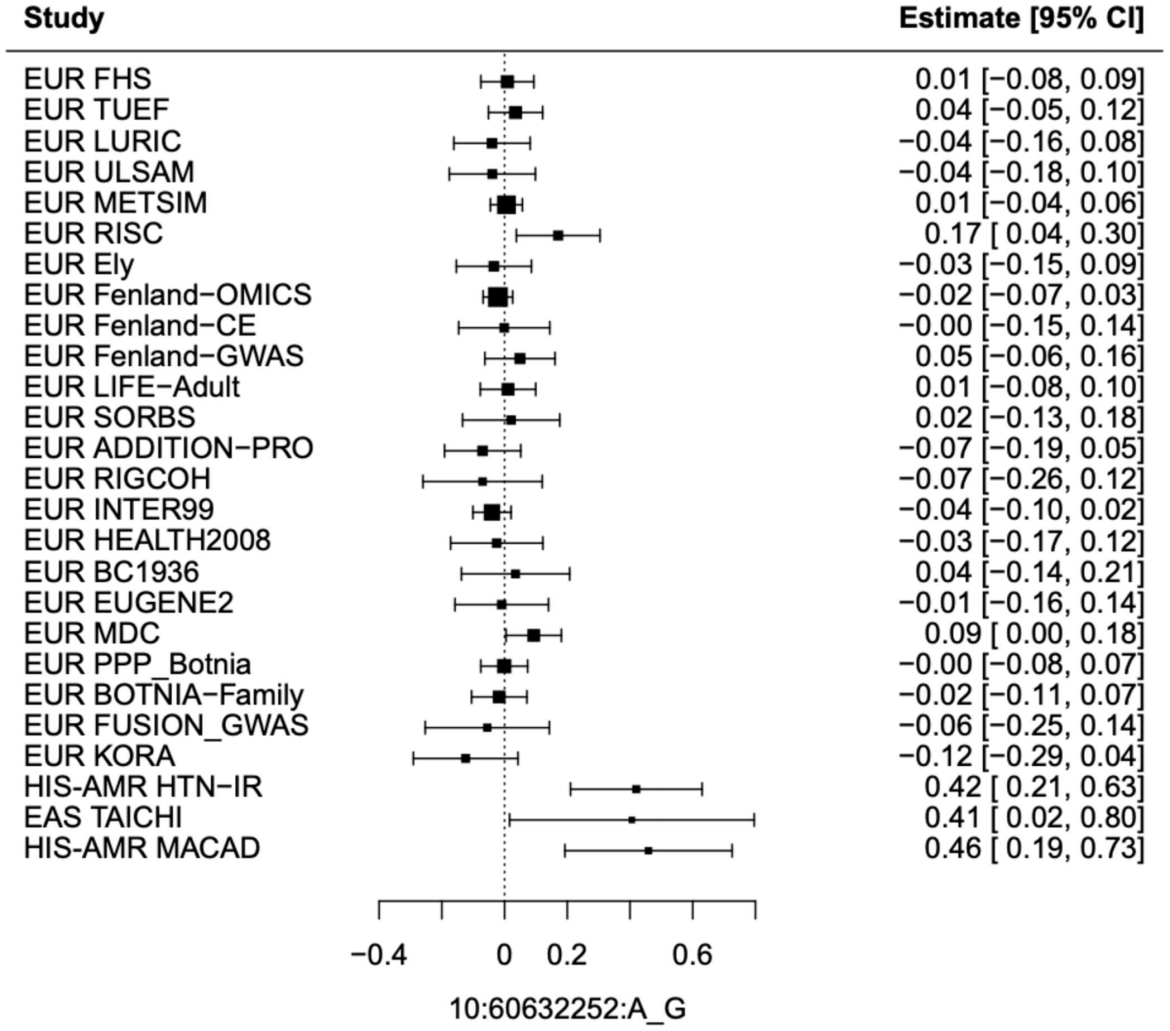
Forest plot of beta estimates for the association of rs60453193 with insulin fold change in individual cohorts. Labels on the right-hand side indicate the ancestry of the study and study name. EUR-European ancestry, HISAMR – Hispanic American ancestry, EAS – East Asian ancestry. Left-hand side values are beta estimate and 95% confidence interval. Error bars denote a 95% confidence interval. X-axis denotes the beta estimate of associations with insulin fold change in BMI adjusted analyses.

**Extended Data Figure 7:**
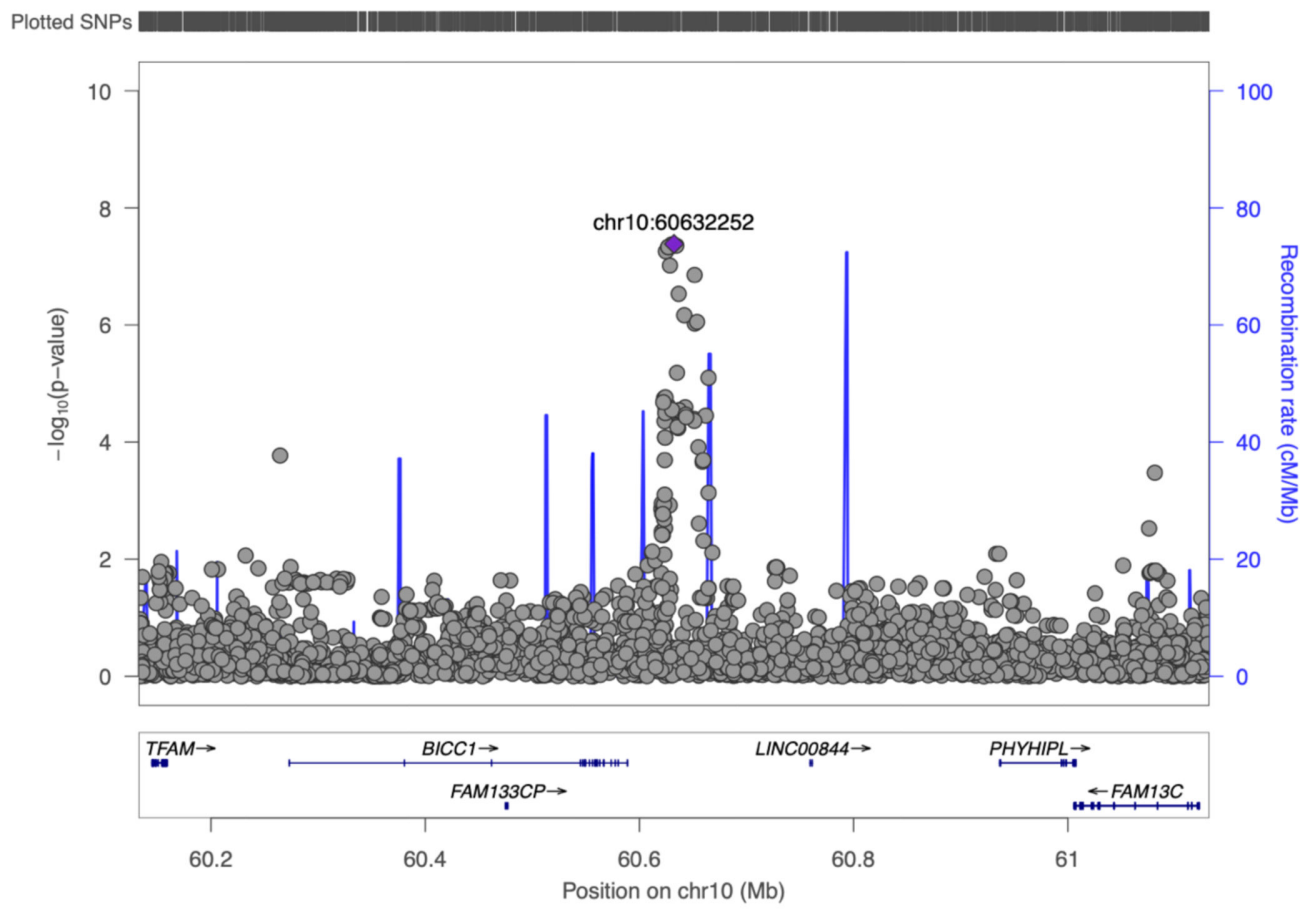
Regional association plot at *BICC1* (rs60453193)for insulin fold change in meta-analysis of non-European cohorts. Unadjusted -log10 p-values are indicated on the y axis. Lead variant indicated by purple diamond.

**Extended Data Figure 8:**
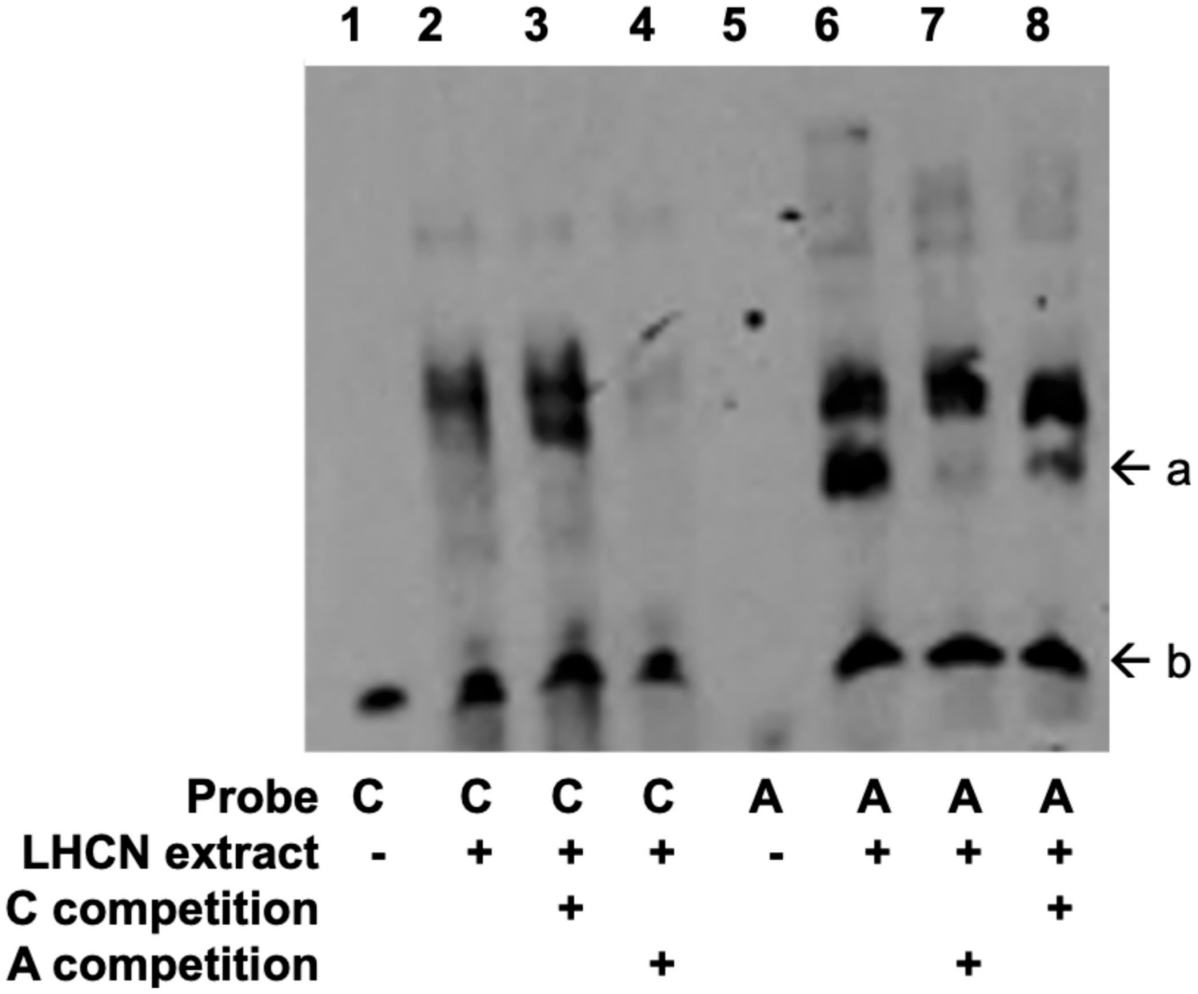
rs117643180 exhibits allelic differences in transcription factor binding. An EMSA using 6μg per lane of nuclear extract from undifferentiated LHCN-M2 cells shows protein-DNA interactions for probes centered around each both alleles of rs117643180. The probe containing rs117643180-A shows allele-specific protein binding (Arrow A, lane 6), relative to the probe containing rs117643180-C (lane 2). A 25-fold excess unlabeled probe containing the A allele competed away A-specific bands more effectively (lane 7) than 25-fold excess unlabeled probe containing the C allele (lane 8). Arrow B shows a biotinylated free probe (200 fmol per lane). Uncropped image is available in Source Data.

**Extended Data Figure 9:**
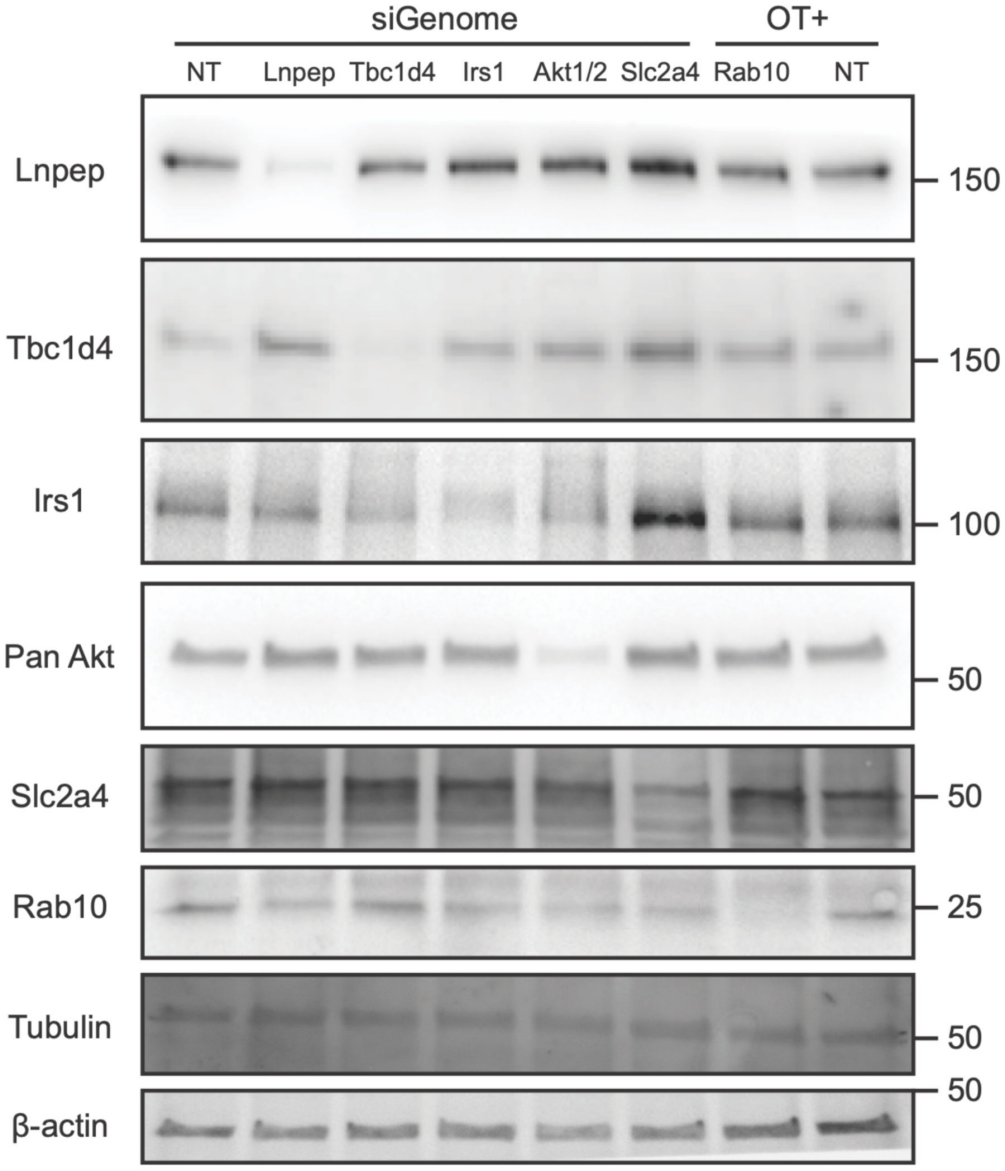
Confirmation of knockdown of positive control genes in wildtype 3T3-L1 adipocytes by Western Blot. Representative blot from N=2. Marker indicates protein size in kDa is outlined on the right-hand side of the blot. siGenome and OT+ represent siRNA pools with their corresponding targets indicated below (see methods) and NT denotes non-targeting control. Antibodies are outlined on the left-hand side of the blot with Tubulin and B-actin used as loading controls. Uncropped blots are provided in Source Data.

## Supplementary Material

Supplementary Note

Supplementary Tables

## Figures and Tables

**Table 1:** Genome wide significant loci for Insulin fold change and Modified Stumvoll ISI, adjusted for BMI in meta-analysis of cohorts of European ancestry.

Trait	Locus	CHR:POS	rsid	EA	OA	EAF	Beta	SE	P-value	N
Insulin Fold Change	*MTN1RB*	11:92673828	rs1387153	T	C	0.3023	0.0438	0.0069	2.25E-10	52698
	*PPP1R3B* (primary)	8:9173358	rs7012814	A	G	0.4796	0.0481	0.0065	1.35E-13	52458
	*PPP1R3B* (secondary)	8:9185146	rs2126259	T	C	0.1265	-0.0688	0.0099	2.86E-12	52698
	*C2CD4A*	15:62396189	rs7167878	A	C	0.4606	-0.0426	0.0064	2.76E-11	53287
	*SLC2A4*	17:7185779	rs11764318 0	A	C	0.0231	0.1428	0.0221	1.11E-10	50671
Modified Stumvoll ISI	*IRS1*	2:227101411	rs2972144	A	G	0.366	0.0644	0.0067	1.10E-21	50511
	*COBLL1*	2:165558252	rs12692738	T	C	0.7724	-0.0568	0.0075	5.24E-14	53657
	*PPARG*	3:12385828	rs11128603	A	G	0.8595	-0.0635	0.0091	2.64E-12	53657
	*SLC2A4*	17:7185779	rs11764318 0	A	C	0.0231	-0.1416	0.0221	1.58E-10	51187
	*C5orf67*	5:55806751	rs459193	A	G	0.2816	0.0409	0.007	6.18E-09	53657
	*MTOR*	1:11322628	rs2295080	T	G	0.6961	-0.0394	0.0069	1.34E-08	53657
	*BCL2*	18:60845884	rs12454712	T	C	0.5858	-0.0374	0.0068	4.42E-08	52701
	*FAM101A*	12:124539537	rs1906937	A	C	0.2894	-0.0384	0.007	4.86E-08	53657

EA – effect allele, OA – other allele, EAF – effect allele frequency, SE – standard error. CHR:POS – chromosome:position GRCh37 (hg19).

## Data Availability

GWAS summary statistics will be made available on the MAGIC Investigators Website (https://magicinvestigators.org/downloads/) and GWAS catalog (https://www.ebi.ac.uk/gwas/home): GCST90267567, GCST90267568, GCST90267569, GCST90267570, GCST90267571, GCST90267572, GCST90267573, GCST90267574, GCST90267575, GCST90267576, GCST90267577, GCST90267578. Data from the Fenland cohort can be requested by bona fide researchers for specified scientific purposes via the study website (https://www.mrc-epid.cam.ac.uk/research/studies/fenland/information-for-researchers/). Data will either be shared through an institutional data sharing agreement or arrangements will be made for analyses to be conducted remotely without the necessity for data transfer. All data used in genetic risk score association analyses are available from the UK Biobank (UKBB) upon application (https://www.ukbiobank.ac.uk). All analyses in UKBB in this manuscript were conducted under application # 44448. Further details about the RISC Study and data availability can be found here: http://www.egir.org/egirrisc/. The Genotype-Tissue Expression (GTEx) Project was supported by the Common Fund of the Office of the Director of the National Institutes of Health, and by NCI, NHGRI, NHLBI, NIDA, NIMH, and NINDS. The data used for the analyses described in this manuscript can be obtained from the GTEx Portal (https://www.gtexportal.org/home/) and dbGaP accession number phs000424.v8.p2. Genome regulatory annotations from ENCODE (https://www.encodeproject.org/) and Roadmap Epigenomics Consortium (https://egg2.wustl.edu/roadmap/web_portal/) were explored via UCSC Genome Browser (http://genome.ucsc.edu). Published differentiated 3T3-L1 RNA sequencing data used in this study is available from GEO accession GSE129957 (https://www.ncbi.nlm.nih.gov/geo/)

## Code Availability

No previously unreported custom code or algorithm was used to generate results. The following software and packages were used for data analysis: METAL v.2011-03-25 (http://csg.sph.umich.edu/abecasis/Metal/download/), random-metal v2017-07-24 (https://github.com/explodecomputer/random-metal), LD score regression v.1.0.1 (https://github.com/bulik/ldsc), R v 3.6.0 and v.4.0.3 (https://www.r-project.org/). R packages coloc v 5.1.0 (https://cran.r-project.org/web/packages/coloc/), Hyprcoloc v1.0 (https://github.com/jrs95/hyprcoloc).

GCTA 1.26.0 (https://yanglab.westlake.edu.cn/software/gcta/#Overview). Easy QC v17.8 (https://www.uni-regensburg.de/medizin/epidemiologie-praeventivmedizin/genetische-epidemiologie/software/index.html). Associated code and scripts used in this manuscript are available on GitHub: https://github.com/MRC-Epid/GWAS_postchallenge_insulin.^87^

## References

[1] James DE, Stöckli J, Birnbaum MJ (2021). The aetiology and molecular landscape of insulin resistance. Nat Rev Mol Cell Biol.

[2] Defronzo RA (2009). From the Triumvirate to the Ominous Octet: A New Paradigm for the Treatment of Type 2 Diabetes Mellitus. Diabetes.

[3] Chen J (2021). The trans-ancestral genomic architecture of glycemic traits. Nat Genet.

[4] Scott RA (2012). Large-scale association analyses identify new loci influencing glycemic traits and provide insight into the underlying biological pathways. Nat Genet.

[5] Lagou V (2021). Sex-dimorphic genetic effects and novel loci for fasting glucose and insulin variability. Nat Commun.

[6] Taylor R (1996). Direct assessment of liver glycogen storage by 13C nuclear magnetic resonance spectroscopy and regulation of glucose homeostasis after a mixed meal in normal subjects. J Clin Invest.

[7] Jue T, Rothman DL, Tavitian BA, Shulman RG (1989). Natural-abundance 13C NMR study of glycogen repletion in human liver and muscle. Proc Natl Acad Sci U S A.

[8] Petersen MC, Shulman GI (2018). Mechanisms of Insulin Action and Insulin Resistance. Physiol Rev.

[9] Fischer Y (1997). Insulin-induced Recruitment of Glucose Transporter 4 (GLUT4) and GLUT1 in Isolated Rat Cardiac Myocytes: Evidence Of The Existence Of Different Intracellular Glut4 Vesicle Populations. J Biol Chem.

[10] Goodyear LJ (1996). Glucose Ingestion Causes GLUT4 Translocation in Human Skeletal Muscle. Diabetes.

[11] Kahn BB (1994). Dietary Regulation of Glucose Transporter Gene Expression: Tissue Specific Effects in Adipose Cells and Muscle. J Nutr.

[12] Maianu L, Keller SR, Garvey WT (2001). Adipocytes Exhibit Abnormal Subcellular Distribution and Translocation of Vesicles Containing Glucose Transporter 4 and Insulin-Regulated Aminopeptidase in Type 2 Diabetes Mellitus: Implications Regarding Defects in Vesicle Trafficking. J Clin Endocrinol Metab.

[13] Rothman DL (1995). Decreased muscle glucose transport/phosphorylation is an early defect in the pathogenesis of non-insulin-dependent diabetes mellitus. Proc Natl Acad Sci U S A.

[14] DeFronzo RA, Tripathy D (2009). Skeletal Muscle Insulin Resistance Is the Primary Defect in Type 2 Diabetes. Diabetes Care.

[15] Sano H (2003). Insulin-stimulated phosphorylation of a Rab GTPase-activating protein regulates GLUT4 translocation. J Biol Chem.

[16] Dash S (2009). A truncation mutation in TBC1D4 in a family with acanthosis nigricans and postprandial hyperinsulinemia. Proc Natl Acad Sci U S A.

[17] Grarup N (2018). Identification of novel high-impact recessively inherited type 2 diabetes risk variants in the Greenlandic population. Diabetologia.

[18] Moltke I (2014). A common Greenlandic TBC1D4 variant confers muscle insulin resistance and type 2 diabetes. Nature.

[19] Tam CS (2012). Defining insulin resistance from hyperinsulinemic-euglycemic clamps. Diabetes Care.

[20] Reinauer H (1990). Determination of glucose turnover and glucose oxidation rates in man with stable isotope tracers. J Clin Chem Clin Biochem.

[21] Muniyappa R, Lee S, Chen H, Quon MJ (2008). Current approaches for assessing insulin sensitivity and resistance in vivo: Advantages, limitations, and appropriate usage. Am J Physiol-Endocrinol Metab.

[22] Stumvoll M (2000). Use of the oral glucose tolerance test to assess insulin release and insulin sensitivity. Diabetes Care.

[23] Walford GA (2016). Genome-wide association study of the modified stumvoll insulin sensitivity index identifies BCL2 and FAM19A2 as novel insulin sensitivity loci. Diabetes.

[24] Dimas AS (2014). Impact of Type 2 Diabetes Susceptibility Variants on Quantitative Glycemic Traits Reveals Mechanistic Heterogeneity. Diabetes.

[25] DeFronzo RA, Tobin JD, Andres R (1979). Glucose clamp technique: A method for quantifying insulin secretion and resistance. Am J Physiol Endocrinol Metab Gastrointest Physiol.

[26] Vujkovic M (2020). Discovery of 318 new risk loci for type 2 diabetes and related vascular outcomes among 1.4 million participants in a multi-ancestry meta-analysis. Nat Genet.

[27] Zhu Y, Wang L, Yin Y, Yang E (2017). Systematic analysis of gene expression patterns associated with postmortem interval in human tissues. Sci Rep.

[28] Uhlén M (2015). Tissue-based map of the human proteome. Science (80-).

[29] Aguet F (2020). The GTEx Consortium atlas of genetic regulatory effects across human tissues. Science (80-).

[30] Lonsdale J (2013). The Genotype-Tissue Expression (GTEx) project. Nature Genetics.

[31] Kanai F (1993). Insulin-stimulated GLUT4 translocation is relevant to the phosphorylation of IRS-1 and the activity of PI3-kinase. Biochem Biophys Res Commun.

[32] Keller SR, Scott HM, Mastick CC, Aebersold R, Lienhard GE (1995). Cloning and Characterization of a Novel Insulin-regulated Membrane Aminopeptidase from Glut4 Vesicles. J Biol Chem.

[33] Chi NW, Lodish HF (2000). Tankyrase is a golgi-associated mitogen-activated protein kinase substrate that interacts with IRAP in GLUT4 vesicles. J Biol Chem.

[34] Guo HL (2012). The Axin/TNKS complex interacts with KIF3A and is required for insulin-stimulated GLUT4 translocation. Cell Res.

[35] Hook SC (2020). TBC1D1 interacting proteins, VPS13A and VPS13C, regulate GLUT4 homeostasis in C2C12 myotubes. Sci Rep.

[36] Klip A, McGraw TE, James DE (2019). Thirty sweet years of GLUT4.

[37] Stenbit AE (1997). GLUT4 heterozygous knockout mice develop muscle insulin resistance and diabetes. Nat Med.

[38] Gual P, Le Marchand-Brustel Y, Tanti JF (2005). Positive and negative regulation of insulin signaling through IRS-1 phosphorylation. Biochimie.

[39] Barroso I Dominant negative mutations in human PPARγ associated with severe insulin resistance, diabetes mellitus and hypertension. Nature.

[40] Li Q (2019). The Protein Phosphatase 1 Complex Is a Direct Target of AKT that Links Insulin Signaling to Hepatic Glycogen Deposition. Cell Rep.

[41] Agius L (2015). Role of glycogen phosphorylase in liver glycogen metabolism. Mol Aspects Med.

[42] Yoon MS (2017). The Role of Mammalian Target of Rapamycin (mTOR) in Insulin Signaling. Nutrients.

[43] Kuo T (2019). Identification of C2CD4A as a human diabetes susceptibility gene with a role in β cell insulin secretion. Proc Natl Acad Sci U S A.

[44] Lyssenko V (2009). Common variant in MTNR1B associated with increased risk of type 2 diabetes and impaired early insulin secretion. Nat Genet.

[45] Huang S, Czech MP (2007). The GLUT4 Glucose Transporter. Cell Metab.

[46] Degrandmaison J (2020). In vivo mapping of a GPCR interactome using knockin mice. Proc Natl Acad Sci U S A.

[47] Mani M (2019). DRG2 knockdown induces Golgi fragmentation via GSK3β phosphorylation and microtubule stabilization. Biochim Biophys acta Mol cell Res.

[48] Mani M (2016). Developmentally regulated GTP-binding protein 2 coordinates Rab5 activity and transferrin recycling. Mol Biol Cell.

[49] Gendre D (2011). Conserved Arabidopsis ECHIDNA protein mediates trans-Golgi-network trafficking and cell elongation. Proc Natl Acad Sci U S A.

[50] Gonzales PA (2009). Large-scale proteomics and phosphoproteomics of urinary exosomes. J Am Soc Nephrol.

[51] Wang T, Liu NS, Seet LF, Hong W (2010). The emerging role of VHS domaincontaining Tom1, Tom1L1 and Tom1L2 in membrane trafficking. Traffic.

[52] Liu H (2022). ALKBH5-mediated m6A demethylation of GLUT4 mRNA promotes glycolysis and resistance to HER2-targeted therapy in breast cancer. Cancer Res.

[53] Pulit SL (2019). Meta-analysis of genome-wide association studies for body fat distribution in 694 649 individuals of European ancestry. Hum Mol Genet.

[54] Dunham I (2012). An integrated encyclopedia of DNA elements in the human genome. Nature.

[55] Kundaje A (2015). Integrative analysis of 111 reference human epigenomes. Nature.

[56] Ernst J, Kellis M (2012). ChromHMM: automating chromatin-state discovery and characterization. Nat Methods.

[57] Kent WJ (2002). The human genome browser at UCSC. Genome Res.

[58] Stumvoll M, Van Haeften T, Fritsche A, Gerich J (2001). Oral glucose tolerance test indexes for insulin sensitivity and secretion based on various availabilities of sampling times [11]. Diabetes Care.

[59] Lindsay T (2019). Descriptive epidemiology of physical activity energy expenditure in UK adults (The Fenland study). Int J Behav Nutr Phys Act.

[60] Hills SA (2004). The EGIR-RISC Study (the European group for the study of insulin resistance: Relationship between insulin sensitivity and cardiovascular disease risk): I. Methodology and Objectives. Diabetologia.

[61] McCarthy S (2016). A reference panel of 64,976 haplotypes for genotype imputation. Nat Genet.

[62] Auton A (2015). A global reference for human genetic variation. Nature.

[63] Lim ET (2014). Distribution and Medical Impact of Loss-of-Function Variants in the Finnish Founder Population. PLoS Genet.

[64] FinnGen (2020). FinnGen Documentation of R3 release.

[65] Winkler TW (2014). Quality control and conduct of genome-wide association meta-analyses. Tsegaselassie Work.

[66] Willer CJ, Li Y, Abecasis GR (2010). METAL: Fast and efficient meta-analysis of genomewide association scans. Bioinformatics.

[67] Scott RA (2012). Large-scale association analyses identify new loci influencing glycemic traits and provide insight into the underlying biological pathways. Nat Genet.

[68] Yang J (2012). Conditional and joint multiple-SNP analysis of GWAS summary statistics identifies additional variants influencing complex traits. Nat Genet.

[69] GitHub-explodecomputer/random-metal: Adding random effects model to the METAL software.

[70] Wakefield J (2009). Bayes factors for genome-wide association studies: comparison with P-values. Genet Epidemiol.

[71] Giambartolomei C (2014). Bayesian Test for Colocalisation between Pairs of Genetic Association Studies Using Summary Statistics. PLOS Genet.

[72] Wang Q (2021). Rare variant contribution to human disease in 281,104 UK Biobank exomes. Nat.

[73] Bulik-Sullivan B (2015). LD Score regression distinguishes confounding from polygenicity in genome-wide association studies. Nat Genet.

[74] Finucane HK (2018). Heritability enrichment of specifically expressed genes identifies disease-relevant tissues and cell types. Nat Genet.

[75] Foley CN (2021). A fast and efficient colocalization algorithm for identifying shared genetic risk factors across multiple traits. Nat Commun.

[76] Zhu CH (2007). Cellular senescence in human myoblasts is overcome by human telomerase reverse transcriptase and cyclin-dependent kinase 4: Consequences in aging muscle and therapeutic strategies for muscular dystrophies. Aging Cell.

[77] Fogarty MP, Cannon ME, Vadlamudi S, Gaulton KJ, Mohlke KL (2014). Identification of a Regulatory Variant That Binds FOXA1 and FOXA2 at the CDC123/CAMK1D Type 2 Diabetes GWAS Locus. PLoS Genet.

[78] Roman TS (2017). A type 2 diabetes-associated functional regulatory variant in a pancreatic islet enhancer at the ADCY5 locus. Diabetes.

[79] Lotta LA (2017). Integrative genomic analysis implicates limited peripheral adipose storage capacity in the pathogenesis of human insulin resistance. Nat Genet.

[80] Leland Taylor D (2019). Integrative analysis of gene expression, DNA methylation, physiological traits, and genetic variation in human skeletal muscle. Proc Natl Acad Sci U S A.

[81] Kuhn RM, Haussler D, Kent WJ (2013). The UCSC genome browser and associated tools. Brief Bioinform.

[82] Sun W (2020). A Transcriptomic Analysis Reveals Novel Patterns of Gene Expression During 3T3-L1 Adipocyte Differentiation. Front Mol Biosci.

[83] Ng Y, Ramm G, Lopez JA, James DE (2008). Rapid activation of Akt2 is sufficient to stimulate GLUT4 translocation in 3T3-L1 adipocytes. Cell Metab.

[84] Kohn AD, Summers SA, Birnbaum MJ, Roth RA (1996). Expression of a constitutively active Akt Ser/Thr kinase in 3T3-L1 adipocytes stimulates glucose uptake and glucose transporter 4 translocation. J Biol Chem.

[85] Tucker DF (2018). Isolation of state-dependent monoclonal antibodies against the 12-transmembrane domain glucose transporter 4 using virus-like particles. Proc Natl Acad Sci U S A.

[86] Diaz-Vegas A (2023). A high-content endogenous GLUT4 trafficking assay reveals new aspects of adipocyte biology. Life Sci alliance.

[87] https://zenodo.org/record/7805583#.ZC7C_exBxhE.

