# Supplementary material for: Genome-wide association study and functional characterisation identifies candidate genes for insulin-stimulated glucose uptake
Source: Nat Genet. Author manuscript; Available in PMC 2023 Jul 14. (PMC7614755; doi:10.1038/s41588-023-01408-9)
Supplement: Supplementary Note [file EMS178611-supplement-Supplementary_Note.pdf]

Supplementary Note:

Contents

Supplementary Note: 1

Study Acknowledgements 3

Individual Acknowledgments 5

Supplementary Methods 8

Genetic Risk Scores for ISI and IFC 8

LD Score Regression 8

Confirmation of gene knockdown in 3T3-L1 adipocytes 9

1. Correlation of post-challenge insulin traits with other metabolic traits in the Fenland Study and RISC cohort 12

2. Genetic Discovery of post-challenge insulin resistance: European-only meta-analyses 14

Supplementary Figure 1: Manhattan and QQ plots for European-only meta-analysis of Modified Stumvoll ISI adjusted for BMI 15

Supplementary Figure 2: Manhattan and QQ plots for European-only meta-analysis of Modified Stumvoll ISI unadjusted for BMI 15

Supplementary Figure 3: Manhattan and QQ plots for European-only meta-analysis of insulin fold change adjusted for BMI 16

Supplementary Figure 4: Manhattan and QQ plots for European-only meta-analysis of insulin fold change unadjusted for BMI 17

3. Regional plots of genetic loci associated with Modified Stumvoll ISI adjBMI (Supplementary Figure 5) 18

Supplementary Figure 5: Regional association plots of genetic loci associated with Modified Stumvoll ISI adjBMI in European-only meta-analysis. 18

4. Regional plots of genetic loci associated with Insulin Fold Change adjusted for BMI (Supplementary Figure 6) 22

Supplementary Figure 6: Regional association plots of genetic loci associated with insulin fold change adjBMI in European-only meta-analysis. 22

5. Genetic Discovery of post-challenge insulin resistance: non-European and multi-ancestry meta-analyses (Supplementary Figures 7-14) 25

Supplementary Figure 7: Manhattan and QQ plots for multi-ancestry (EUR, HISAMR, EAS) meta-analysis Insulin Fold Change adjusted for BMI 25

Supplementary Figure 8: Manhattan and QQ plots for multi-ancestry (EUR, HISAMR, EAS) meta-analysis Insulin Fold Change unadjusted for BMI 26

Supplementary Figure 9: Manhattan and QQ plots for multi-ancestry (EUR, HISAMR, EAS) meta-analysis Modified Stumvoll ISI adjusted for BMI 27

Supplementary Figure 10: Manhattan and QQ plots for multi-ancestry (EUR, HISAMR, EAS) meta-analysis Modified Stumvoll ISI adjusted for BMI 28

Supplementary Figure 11: Manhattan and QQ plots for non-European Ancestry (HISAMR, EAS) meta-analysis Insulin Fold Change adjusted for BMI 29

Supplementary Figure 12: Manhattan and QQ plots for non-European Ancestry (HISAMR, EAS) meta-analysis Insulin Fold Change unadjusted for BMI 30

Supplementary Figure 13: Manhattan and QQ plots for non-European Ancestry (HISAMR, EAS) meta-analysis Modified Stumvoll ISI adjusted for BMI 31

Supplementary Figure 14: Manhattan and QQ plots for non-European Ancestry (HISAMR, EAS) meta-analysis Modified Stumvoll ISI unadjusted for BMI 32

6. rs60453193 (chr10:60632252_A_G (b37)) at *BICC1* is associated with Insulin Fold Change specifically in Non-European Cohorts 33

7. SLC2A4 is in perfect D' with variants reported to be associated with type 2 diabetes in Hispanic American and East Asian ancestries. 34

Supplementary Figure 15: SLC2A4 locus regional association plot for Type 2 Diabetes across studies in different ancestry groups. 35

Supplementary Figure 16: LD matrix for lead SNPs in IFC, ISI and T2D GWAS at SLC2A4 locus in European ancestry 36

8. Genetic associations of IFC and ISI with other cardiometabolic traits........ 38

9. Colocalisation of post-challenge insulin resistance and eQTL for SLC2A4 in skeletal muscle at the SLC2A4 locus 39

10. rs117643180 (*SLC2A4*) affects expression of GLUT4 in skeletal muscle through changes in transcriptional regulation 42

Supplementary Figure 18: rs117643180 exhibits allelic differences in transcriptional activity. 42

11. Tissue of Action at post-challenge insulin reflects loci reflect tissues implicated in post-prandial insulin action. 43

12. Integration of additional phenotypic layers identifies additional loci implicated in post-challenge insulin action 44

Supplementary Figure 19: Integrated GWAS approach highlights additional loci including those implicating glucose transport. 47

Supplementary Figure 20: Assessment of colocalisation at loci associated with IFC or ISI with related traits 48

Supplementary Figure 22: Overall screen surface/total GLUT4 comparison for HA-GLUT4-mRuby and WT 3T3-L1 adipocytes........ 50

Supplementary Figure 23: Average percentage of mRNA detected in knockdown samples compared to NT control. 51

N = 2-3 independent biological replicates per gene, points represent independent samples. Error bar represents +/- standard error of mean. 51

Supplementary Figure 24: Glucose Transport relative to NT control........ 52

Supplementary Figure 25: Visualisation and quantification of impact on parameters of interest of knockdown Slc36a4 and Pdzk1ip1 in 3T3-L1 adipocytes........ 53

References for Supplementary Note: 54

Study Acknowledgements

Study	Acknowledgements
ADDITION-PRO	The ADDITION-PRO study was funded by an unrestricted grant from the European Foundation for the Study of Diabetes/Pfzer for Research into Cardiovascular Disease Risk Reduction in Patients with Diabetes (74550801), by the Danish Council for Strategic Research and by internal research and equipment funds from Steno Diabetes Center
Botnia Family Study	The Botnia Family Study and the PPP Botnia Study have been financially supported by grants from Folkhälsan Research Foundation, the Sigrid Juselius Foundation, The Academy of Finland (grants no. 263401, 267882, 312063 and 336822 to LG; 312072 and 336826 to TT), University of Helsinki, Nordic Center of Excellence in Disease Genetics, EU (EXGENESIS, MOSAIC FP7-600914), Ollqvist Foundation, Swedish Cultural Foundation in Finland, Finnish Diabetes Research Foundation, Foundation for Life and Health in Finland, Signe and Ane Gyllenberg Foundation, Finnish Medical Society, Paavo Nurmi Foundation, State Research Funding via the Helsinki University Hospital, Perklén Foundation, Närpes Health Care Foundation and Ahokas Foundation. The study has also been supported by the Ministry of Education in Finland, Municipal Heath Care Center and Hospital in Jakobstad and Health Care Centers in Vasa, Närpes and Korsholm. The research leading to these results has received funding from the European Research Council under the European Union's Seventh Framework Programme (FP7/2007-2013) / ERC grant agreement n° 269045. The skillful assistance of the Botnia Study Group is gratefully acknowledged.
Botnia PPP	The Botnia Family Study and the PPP Botnia Study have been financially supported by grants from Folkhälsan Research Foundation, the Sigrid Juselius Foundation, The Academy of Finland (grants no. 263401, 267882, 312063 and 336822 to LG; 312072 and 336826 to TT), University of Helsinki, Nordic Center of Excellence in Disease Genetics, EU (EXGENESIS, MOSAIC FP7-600914), Ollqvist Foundation, Swedish Cultural Foundation in Finland, Finnish Diabetes Research Foundation, Foundation for Life and Health in Finland, Signe and Ane Gyllenberg Foundation, Finnish Medical Society, Paavo Nurmi Foundation, State Research Funding via the Helsinki University Hospital, Perklén Foundation, Närpes Health Care Foundation and Ahokas Foundation. The study has also been supported by the Ministry of Education in Finland, Municipal Heath Care Center and Hospital in Jakobstad and Health Care Centers in Vasa, Närpes and Korsholm. The research leading to these results has received funding from the European Research Council under the European Union's Seventh Framework Programme (FP7/2007-2013) / ERC grant agreement n° 269045. The skillful assistance of the Botnia Study Group is gratefully acknowledged.
DIAGEN	The DIAGEN study was supported by the Commission of the European Communities, Directorate C -Public Health and Risk Assessment, Health & Consumer Protection, Grant Agreement number - 2004310 and by the Dresden University of Technology Funding Grant, Med Drive. We are grateful to all of the patients who cooperated in this study and to their referring physicians and diabetologists in Saxony.
DPS	The DPS has been financially supported by grants from the Academy of Finland (117844 and 40758, 211497, and 118590; The EVO funding of the Kuopio University Hospital from Ministry of Health and Social Affairs
DR's EXTRA Study	The DR's EXTRA study was supported by grants from Ministry of Education and Culture of Finland, Academy of Finland, European Commission FP6 Integrated Project (EXGENESIS), LSHM-CT-2004-005272, City of Kuopio, Juho Vainio Foundation, Finnish Diabetes Association, Finnish Foundation for Cardiovascular Research, Kuopio University Hospital, Päivikki and Sakari Sohlberg Foundation, Social Insurance Institution of Finland.
Ely	The Ely study was supported by the Medical Research Council (MC_UU_12015/1) and NHS Research and Development.
FHS	The Framingham Heart Study (FHS) was supported by the National Heart, Lung and Blood Institute's Framingham Heart Study Contract Nos. N01-HC-25195, HHSN268201500001I amd 75N92019D00031, and its contract with Affymetrix, Inc for genotyping services (Contract No. N02-HL-6-4278).
FUSION GWAS	The study was supported by the National Institutes of Health (R01 DK062370, DK072193, DK093757; ZIA HG000024)

FUSION2	The study was supported by the National Institutes of Health (R01 DK062370, DK072193; ZIA HG000024)
Health2008	Health2008 was supported by the Timber Merchant Vilhelm Bang's Foundation, the Danish Heart Foundation (Grant number 07-10-R61-A1754-B838-22392F), and the Health Insurance Foundation (Helsefonden) (Grant number 2012B233).
HTN-IR / MACAD	Supported in part by contacts HL-088457 (MACAD), HL-0697974 (HTN-IR), HL-055798 (NIDDM-Athero), and DK-079888. Andy by the National Center for Advancing Translational Sciences, CTSI grant UL1TR001881, and the National Institute of Diabetes and Digestive and Kidney Disease Diabetes Research Center (DRC) grant DK063491 to the Southern California Diabetes Endocrinology Research Center. Infrastructure for the CHARGE Consortium is supported in part by the National Heart, Lung, and Blood Institute (NHLBI) grant R01HL105756
Inter99	The study was financially supported by research grants from the Danish Research Council, the Danish Centre for Health Technology Assessment, Novo Nordisk Inc., Research Foundation of Copenhagen County, Ministry of Internal Affairs and Health, the Danish Heart Foundation, the Danish Pharmaceutical Association, the Augustinus Foundation, the Ib Henriksen Foundation, the Becket Foundation, and the Danish Diabetes Association.
KORA	The KORA study was initiated and financed by the Helmholtz Zentrum München – German Research Center for Environmental Health, which is funded by the German Federal Ministry of Education and Research (BMBF) and by the State of Bavaria.
LIFE-Adult	LIFE-Adult was funded by the Leipzig Research Center for Civilization Diseases (LIFE). LIFE was funded by means of the European Union, the European Regional Development Fund (ERDF, grant 713-24120) and by means of the Free State of Saxony within the framework of the excellence initiative (14505/2470).
LURIC	We thank all participants of the LURIC study, as well as the study teams who were either temporarily or permanently involved in patient recruitment as well as sample and data handling. We also thank the laboratory staff at the Ludwigshafen General Hospital and the Universities of Freiburg, Ulm, and Heidelberg in Germany. The genotyping of the LURIC participants was supported by the 7th Framework Programs AtheroRemo (grant agreement number 201668) and RiskyCAD (grant agreement number 305739) of the European Union. LURIC received further support by the H2020 Programs TO_AITION (grant agreement number 848146) and TIMELY (grant agreement number 101017424) of the European Union and the Competence Cluster of Nutrition and Cardiovascular Health (nutriCARD), which is funded by the German Federal Ministry of Education and Research (grant agreement number 01EA1808).
METSIM	The METSIM study was supported by the Academy of Finland (321428) and Sigrid Juselius Foundation.
RIGCOH	The RigCoh-study was supported by The Lundbeck Foundation, Civilingeniør H. C. Bechgaard's Foundation, The Danish Diabetes Association, The Danish Medical Research Council, the Gangsted Foundation, A. P. Moeller og Hustru Chastine Mc-Kinney Moeller's Foundation, Aase og Ejner Danielsen's Foundation, The Augustinus Foundation, and The Research Foundation of Rigshospitalet
RISC	The RISC study was supported by the EU 5th Framework (EU contract QLG1-CT-2001-01252) with additional funding from AstraZeneca.
TAICHI-G	The TAICHI-G study was supported by grants from the National Health Research Institutes, Taiwan (PH-099-PP-03, PH-100-PP-03, and PH-101-PP-03); the National Science Council, Taiwan (NSC 101-2314-B-075A-006-MY3, MOST 104-2314-B-075A-006-MY3, MOST 104-2314-B-075A-007, and MOST 105-2314-B-075A-003); and the Taichung Veterans General Hospital, Taiwan (TCVGH-1020101C, TCVGH-1020102D, TCVGH- 1023102B, TCVGH-1023107D, TCVGH-1030101C, TCVGH- 1030105D, TCVGH-1033503C, TCVGH-1033102B, TCVGH-1033108D, TCVGH-1040101C, TCVGH-1040102D, TCVGH-1043504C, and TCVGH-1043104B). The provision of genotyping data was supported in part by the National Center for Advancing Translational Sciences, CTSI grant UL1TR001881, and the National Institute of Diabetes and Digestive and Kidney Disease Diabetes Research (DRC) grant DK063491
The Fenland Study	The Fenland Study (10.22025/2017.10.101.00001) is funded by the Medical Research Council (MC UU 12015/1). We are grateful to all the volunteers and to the General

	Practitioners and practice staff for assistance with recruitment. We thank the Fenland Study Investigators, Fenland Study Co-ordination team and the Epidemiology Field, Data and Laboratory teams. We further acknowledge support for genomics from the Medical Research Council (MC PC 13046).
SORBS	This project was supported by grants from the Deutsche Forschungsgemeinschaft (DFG, German Research Foundation – Projektnummer 209933838 – SFB 1052 "Obesity mechanisms") and by the German Diabetes Association.
TUEF	This study was supported in parts by a grant (01GI0925) from the Federal Ministry of Education and Research to the German Center for Diabetes Research.
ULSAM	Genotyping in Swedish cohorts was performed by the SNP&SEQ Technology Platform in Uppsala.

Individual Acknowledgments

First	Middle	Last	Acknowledgement
Emma		*Ahlqvist*	Swedish Research Council (Grant.no 2020-02191)
Mette	*K*	*Andersen*	Novo Nordisk Foundation (grant.nr. NNF18CC0034900)
Inês		Barroso	IB was funded by an "Expanding excellence in England" award from Research England. This study was supported by the National Institute for Health and Care Research Exeter Biomedical Research Centre. The views expressed are those of the author(s) and not necessarily those of the NIHR or the Department of Health and Social Care.
Michael		Boehnke	US NIH R01 DK062370
Lori	L	Bonnycastle	National Institutes of Health, National Human Genome Research Institute, ZIA HG000024
K.	Alaine	Broadway	US National Institutes of Health (NIH) T32HL129982
Yii-Der Ida		Chen	The TAICHI-G study was supported by grants from the National Health Research Institutes, Taiwan (PH-099-PP-03, PH-100-PP-03, and PH-101-PP-03); the National Science Council, Taiwan (NSC 101-2314-B-075A-006-MY3, MOST 104-2314-B-075A-006-MY3, MOST 104-2314-B-075A-007, and MOST 105-2314-B-075A-003); and the Taichung Veterans General Hospital, Taiwan (TCVGH-1020101C, TCVGH-1020102D, TCVGH-1023102B, TCVGH-1023107D, TCVGH-1030101C, TCVGH- 1030105D, TCVGH-1033503C, TCVGH-1033102B, TCVGH-1033108D, TCVGH-1040101C, TCVGH-1040102D, TCVGH-1043504C, and TCVGH-1043104B). The provision of genotyping data was supported in part by the National Center for Advancing Translational Sciences, CTSI grant UL1TR001881, and the National Institute of Diabetes and Digestive and Kidney Disease Diabetes Research (DRC) grant DK063491
Francis	S	Collins	National Institutes of Health, National Human Genome Research Institute, ZIA HG000024
Josée		Dupuis	NIH R01DK078616, UM1DK078616, R01HL151855 Grants: NIH U01 DK078616; NIH R01 HL151855 Individuals supported: James B Meigs (PI of both of these grants), Josée Dupuis, Ching-Ti Liu, Peitao Wu
Michael	R	Erdos	National Institutes of Health, National Human Genome Research Institute, ZIA HG000024

Tove		Fall	T.F. was supported by Swedish Research Council grants (nos. 2015-03477 and 2018-02784), grant support from the Göran Gustafsson Foundation, and by a Starting Grant from the European Research Council (GUTSY - 801965).
Daniel	J	Fazakerley	D.J.F. was supported by an UKRI-MRC Career Development Award MR/S007091/1
Jose	C	Florez	NIH K24 DK110550
Andreas		Fritsche	Deutsche Forschungsgemeinschaft (DFG) Deutsches Zentrum für Diabetesforschung (DZD)
Mark	O	Goodarzi	M.O.G. was supported by the Eris M. Field Chair in Diabetes Research.
Niels		Grarup	Novo Nordisk Foundation (grant.nr. NNF18CC0034900), Independent Research Fund Denmark (grant. nr. 1030-00280B)
Xiuqing		Guo	Supported in part by the American Diabetes Association Distinguished Clinical Scientist Award (BetaGene), HL-088457 (MACAD), HL-0697974 (HTN-IR), HL-055798 (NIDDM-Athero), and DK-079888.(work related to insulin clearance in HTN-IR, MACAD, and NIDDM-Athero). Also supported by the National Center for Advancing Translational Sciences, CTSI grant UL1TR001881, and the National Institute of Diabetes and Digestive and Kidney Disease Diabetes Research Center (DRC) grant DK063491 to the Southern California Diabetes Endocrinology Research Center. Infrastructure for the CHARGE Consortium is supported in part by the National Heart, Lung, and Blood Institute (NHLBI) grant R01HL105756
Torben		*Hansen*	Novo Nordisk Foundation (grant.nr. NNF18CC0034900)
Kurt		Højlund	The RISC Study was supported by European Union grant QLG1-CT-2001-01252, and AstraZeneca and Merck & Co Inc.
Anna		*Jonsson*	Novo Nordisk Foundation (grant.nr. NNF18CC0034900)
Markku		*Laakso*	Grant from Academy of Finland
Timo	A	Lakka	Academy of Finland (211119)
Claudia		Langenberg	Medical Research Council (MC_UU_00006/1 - Etiology and Mechanisms)
Cecilia	M	Lindgren	C.M.L is supported by the Li Ka Shing Foundation, NIHR Oxford Biomedical Research Centre, Oxford, NIH (1P50HD104224-01), Gates Foundation (INV-024200), and a Wellcome Trust Investigator Award (221782/Z/20/Z).
Ching-Ti		Liu	NIH R01DK078616, UM1DK078616, R01HL151855
Jian'an		Luan	Medical Research Council MC_UU_00006/1 - Etiology and Mechanisms
James	B	Meigs	NIH R01DK078616, UM1DK078616, R01HL151855
Karen	L	Mohlke	US NIH R01 DK072193, R01 DK093757, UM1 DK126185
Narisu		Narisu	National Institutes of Health, National Human Genome Research Institute, ZIA HG000024
Stephen		O'Rahilly	S.O. is supported by a Wellcome Investigator award (WT 095515/Z/11/Z) and the NIHR Cambridge Biomedical Research Centre
Rashmi	B	Prasad	Swedish Research Council (2021-02623)

Rainer		Rauramaa	Personal; Ministry of Education and Culture of Finland (722 and 627; 2004-2010); Academy of Finland (102318, 104943, 123885); European Commission FP6 Integrated Project (EXGENESIS), LSHM-CT-2004-005272; Social Insurance Institution of Finland 4/26/2010; Päivikki and Sakari Sohlberg Foundation; Finnish Diabetes Association; Finnish Foundation for Cardiovascular Research. Groups; City of Kuopio; Juho Vainio Foundation; Kuopio University Hospital;
Carsten	*F*	*Rundsten*	Novo Nordisk Foundation (grant.nr. NNF18CC0034900)
Chloé		Sarnowski	NIH R01DK078616, R01HL15185, NIH K99AG066849-02
Kai	P	Savonen	No conflicts of interest. No financial support to declare.
Sara	*E*	*Stinson*	Novo Nordisk Foundation (grant.nr. NNF18CC0034900, NNF18CC0033668)
Michael		*Stumvoll*	This project was supported by grants from the Deutsche Forschungsgemeinschaft (DFG, German Research Foundation – Projektnummer 209933838 – SFB 1052 "Obesity mechanisms") and by the German Diabetes Association.
Sufyan		*Suleman*	Novo Nordisk Foundation (grant.nr. NNF18CC0034900)
Kent	D	Taylor	This study was supported in part by the National Institutes of Health, National Heart, Lung, and Blood Institute contract R01 HL0767711. Also supported by the National Center for Advancing Translational Sciences, CTSI grant UL1TR001881, and the National Institute of Diabetes and Digestive and Kidney Disease Diabetes Research Center (DRC) grant DK063491 to the Southern California Diabetes Endocrinology Research Center. Infrastructure for the CHARGE Consortium is supported in part by the National Heart, Lung, and Blood Institute (NHLBI) grant R01HL105756.
Robert		Wagner	Deutsche Forschungsgemeinschaft (DFG) Deutsches Zentrum für Diabetesforschung (DZD)
Mark		Walker	The RISC Study was supported by European Union grant QLG1-CT-2001-01252, and AstraZeneca and Merck & Co Inc.
Nicholas	J	Wareham	Medical Research Council (MC_UU_00006/1 - Etiology and Mechanisms
Eleanor		Wheeler	Medical Research Council (MC_UU_00006/1 - Etiology and Mechanisms
Alice		Williamson	Medical Research Council (MC_UU_00006/1) - Etiology and Mechanisms; AW holds a PhD studentship funded by the Wellcome Trust
Emma	P	Wilson	US NIH T32GM067553
Peitao		Wu	NIH R01DK078616, R01HL15185
Björn		Zethelius	No conflicts of interest. No financial support to declare.

Supplementary Methods

Genetic Risk Scores for ISI and IFC

We additionally constructed genetic risk scores (GRS) for IFC and ISI including lead variants at primary association signals in Europeans. GRS were constructed using the sum of allele scores for each individual where allele counts were weighted by the effect of this allele on IFC or ISI, adjusted or unadjusted for BMI as appropriate. Weights used in the construction of the GRS are outlined in **Supplementary Table 12**. Allele counts were discrete for genotyped variants (0 = reference, 1 = heterozygous, 2 = homozygous) and weights were aligned to the dosage coding allele prior to weighting of each variant. Weighted allele counts were summed for each individual to generate the GRS. GRS were constructed for ISI and IFC adjusted and unadjusted for BMI in unrelated individuals of European ancestry in UK Biobank[1] (N max = 351,987) and the OMICS subset Fenland Study[2] (N max = 8,925). Blood biochemistry measures in the Fenland Study and UK Biobank were natural log transformed prior to scaling. Phenotypes were prepared as has been previously described.[3–5]

Linear regression models were run for each genetic score and phenotype pair adjusting for age, sex and the 1st 10 genetic principal components to account for population structure. Phenotypes were scaled to a mean of 0 and SD of 1 to allow direct comparison across phenotypes. Significance was considered at a Bonferroni significance threshold in UKBB ($P < 4.5 \times 10^{-4}$; N GRS = 4, N phenotypes = 28) and nominal significance threshold in the Fenland Study ($P < 0.05$).

LD Score Regression

We applied LD score regression[6] using python-based software LDSC (accessed at https://github.com/bulik/ldsc) to assess genome-wide genetic correlations between IFC and ISI with other cardiometabolic traits. We calculated LD scores for ISI, IFC using BMI adjusted summary statistics from our analyses in cohorts of European ancestry. We further calculated LD scores for additional T2D related traits from publicly available summary statistics for 2 h glucose[7], fasting insulin[7], T2D[8] and WHRadjBMI[9] in cohorts of European ancestry. Further pre-computed LD scores were obtained online for additional cardiometabolic traits of interest (https://data.broadinstitute.org/alkesgroup/LDSCORE/). The genetic correlations were

calculated restricting to HapMap3 SNPs with a MAF > 0.05, and we used the 1000G European LD Score reference[6].

Tissue specificity

To dissect the tissue of action of IFC and ISI loci, we used LDSC-SEG to assess the genome-wide enrichment of tissue-specific and cell type specific annotations[10]. In brief, LDSC-SEG employs stratified LD score regression to assess for genome-wide enrichment of sets of specifically expressed autosomal genes for a given trait of interest. We utilised LD scores available for a total of 205 specific tissue and cell types from GTEx (53 tissues; RNA-seq) and Franke Lab dataset which consists of expression data for 152 tissues of cell types in human, mouse, and rat[10]. In addition, we examined LD scores also generated by Finucane *et al* (2018)[10] from chromatin annotation from Roadmap[11] and ENTEx data from ENCODE[12]. Suggestive enrichment was considered at a nominal significance threshold of $P < 0.05$, with no result surviving multiple testing correction.

Confirmation of gene knockdown in 3T3-L1 adipocytes

RNA extraction

RNA was extracted from 3T3-L1 adipocytes cultured in 24-well plates 4 d after siRNA knockdown (see above). Cells were washed 2x in PBS and TRI reagent (Zymo Research) was added to each well. Cells were then flash frozen in the plate on dry ice and stored at -80°C. RNA extraction was conducted in an RNAse free environment using the Zymo Research Directzol RNA Extraction Kit following standard manufacture protocol. Briefly, plates were thawed on ice and cells were lysed by manual cell scraping and incubation at room temperature for 10 min. Samples were mixed with an equal volume of 100% ethanol and added directly to an RNA extraction column. RNA was eluted in 30 µL of RNAse and DNAse free water. All centrifugation steps were performed at room temperature at 12,000 x g.

Reverse transcription

RNA was diluted to a consistent concentration per biological replicate. Random primers (Promega) were annealed in a total volume of 30 µL at 70°C for 5 min, and immediately rapidly cooled on ice, in line with manufacturers' specifications. Reverse transcription was conducted in a total volume of 50 µL for each sample using MMLV Reverse Transcriptase (Promega) and

RNassin RNA inhibitor (Promega). Reverse transcription was conducted using BioRad Tetrad 2 Thermal Cycler at 25°C for 10 minutes, 37°C for 1 h and 85°C for 10 min before being stored at -20°C.

qPCR RNA quantification

Quantitative PCR was conducted using Thermo Fisher TaqMan gene expression assays following the standard manufacturer protocol. Probe specifications for targets of interest are outlined in **Supplementary Table 31**. These were added to master mix containing TaqMan 2x Universal PCR Master Mix (ThermoFisher) and nuclease-free water. Polymerase chain reaction was carried out using QuantStudio 7 or Quantstudio 5 Real-Time PCR system (Thermo Fisher Scientific). *ActB* (Mm04394036_g1) and *Canx* (Mm00500330_m1) were used as housekeeping controls for all samples. The standard pre-set cycling conditions for TaqMan reagents were used.

qPCR data analysis

Double delta CT values (ddCT) were calculated and the fold change relative to the NT control was used to assess knockdown efficiency. The geometric mean CT value of the housekeeping genes *ActB* and *Canx* were used for normalisation for a given sample. One-way ANOVA was used to compare delta CT (dCT) values for a given knockdown target compared to NT control, and significance was considered at FDR < 5%.

SDS-PAGE and immunoblotting

Typically, 10 µg of protein was resolved by SDS-PAGE and transferred to PVDF or nitrocellulose membranes. Membranes were blocked in 5% BSA or skim milk powder in TBS-T (0.1% v/v Tween-20 in Tris-buffered saline) for 1 h followed by overnight incubation at 4°C with specific primary antibodies (listed below). Following incubation with horseradish peroxidase (HRP)-conjugated anti-rabbit or mouse immunoglobulin G (IgG) or Alexa-647-conjugated anti-mouse IgG secondary antibodies for 1 h. Protein bands were visualised using ECL (Thermo Scientific) or 647-fluorescence intensity on the Chemidoc MP (Bio-Rad). The following antibodies were used: Lnpep/IRAP (Cell Signaling Technologies, #6918); TBC1D4 (Cell Signaling Technologies, #2670); IRS1 (Cell Signaling Technologies, #3407); PanAkt (Cell Signaling Technologies, #2920); GLUT4 (polyclonal antibody recognising the C-

terminus of GLUT4 provided by Professor David James, University of Sydney)[13]; Rab10 (Thermo Fisher; # MA5-15670); alpha-tubulin (Merck; #T9026) and beta-actin (Cell Signaling Technologies; #8457).

1. Correlation of post-challenge insulin traits with other metabolic traits in the Fenland Study and RISC cohort

To determine the traits correlated with insulin resistance in the fasted and fed state, we conducted observational correlation analyses for fasting insulin and dynamic insulin traits Modified Stumvoll Insulin Sensitivity Index (ISI) and Insulin Fold Change during an OGTT (IFC), with metabolic traits measured in the Fenland Study (pairwise N= 1454 (c-peptide)-11787). Fasting insulin was positively correlated with many metabolic traits including glycaemic traits fasting and 2hr glucose, proinsulin, HbA1C. Further fasting insulin is positively correlated with LDL triglycerides and liver biomarkers as well as BMI, WHR and blood pressure (**Extended Data Figure 2**).

We observed that both IFC and ISI show strong correlation with measures of insulin and glucose taken at 2hrs during a standard OGTT, however are poorly correlated with fasting measures, suggesting these are primarily capturing the post-challenge insulin response. ISI is additionally inversely correlated with liver-related traits, as seen with fasting insulin (hepatic steatosis score and measures of circulating alkaline phosphatase, gamma-glutamyl transferase, alanine aminotransferase), suggesting that ISI may additionally capture insulin action on the liver in the fasted state (**Extended Data Figure 2**). IFC was not correlated with any liver-related traits, suggesting that this potentially more specific to the post-prandial state. This is further evidenced by observations that ISI is more strongly correlated with HOMA-IR, an index of fasting insulin resistance (**Extended Data Figure 2**). Further, IFC was weakly correlated with ISI (rho = -0.35), compared to other insulin related traits e.g. 2 hour insulin, suggesting that a GWAS of this measure may capture variants that are distinct from those that are driving 2-hour insulin or alternatively that these traits are able to capture different aspects and pathways that contribute to the post-challenge insulin response.

We conducted similar analyses in participants without diabetes in the RISC cohort (N=1,138; methods) where hyperinsulinemic-euglycemic clamp and OGTT-derived measures of insulin sensitivity are available. We assessed and compared the correlation of clamp and OGTT-derived measures of insulin sensitivity with relevant traits, as was done in the Fenland Study above. We replicated the strong positive correlation of IFC with insulin at 120 min during an OGTT (rho = 0.725, P = 4.21e-177) and saw a weak negative correlation of IFC with fasting

insulin during an OGTT (rho = -0.165, P = 4.57e-8) as well as no correlation with insulin taken 20min before clamp (rho = 0.009, P =0.768). We additionally identified a significant inverse correlation between IFC and clamp-derived measure of insulin sensitivity M/I (rho = -0.180, P = 3.29e-9). For ISI we identify strong inverse correlation with insulin at fasting (rho = -0.68, P = 6.38e-149) and 120mins during an OGTT (rho = -0.869, P = 0), as seen in the Fenland Study **(Extended Data Figure 3).** We additionally identified a strong positive correlation of ISI with M/I (rho = 0.522, P = 5.10e-76). Overall correlations were similar and consistent in direction between RISC and Fenland studies for primary overlapping traits of interest.

2. Genetic Discovery of post-challenge insulin resistance: European-only meta-analyses

We performed fixed-effect meta-analyses within cohorts of European ancestry using METAL[14] for IFC and ISI. Manhattan and QQ plots summarising the results can be found in **Supplementary Figures 1-4** below. Unadjusted -log10 P-values are indicated on the y axis of the Manhattan plots. QQ plots show the observed (Y) vs expected (X) -log10 P value. Unadjusted P values are shown. In Manhattan plots on the left, the green points indicate those that meet genome wide significance. Red line indicates genome wide significance (P < 5e-8)_ and blue line represents suggestive significance (P < 1e-5).

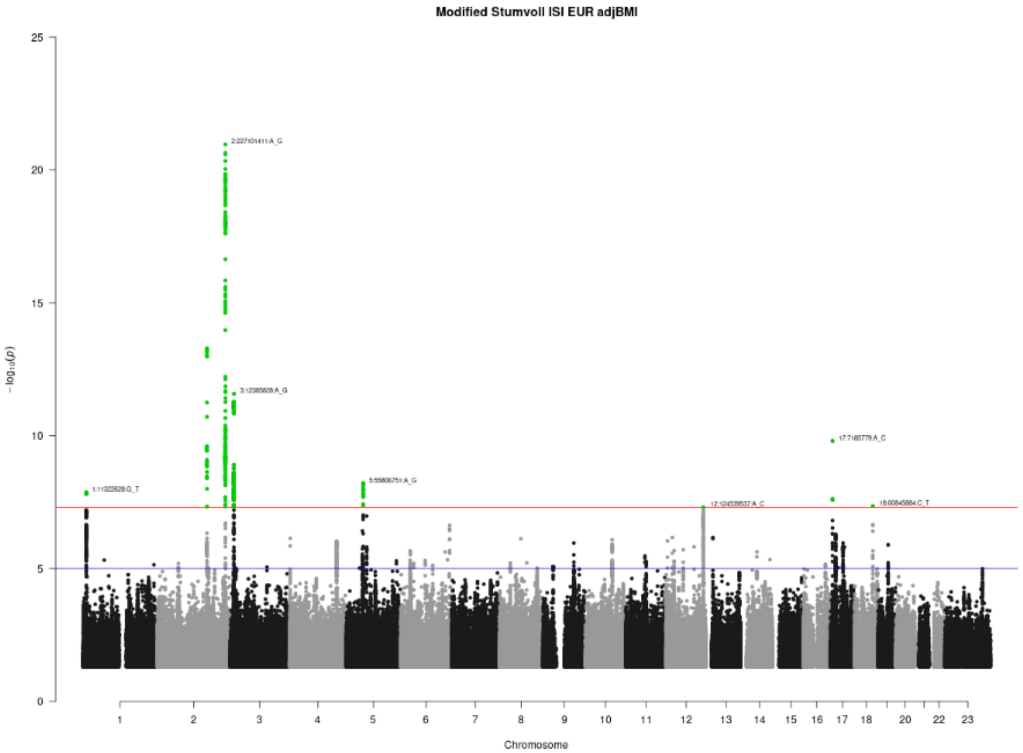

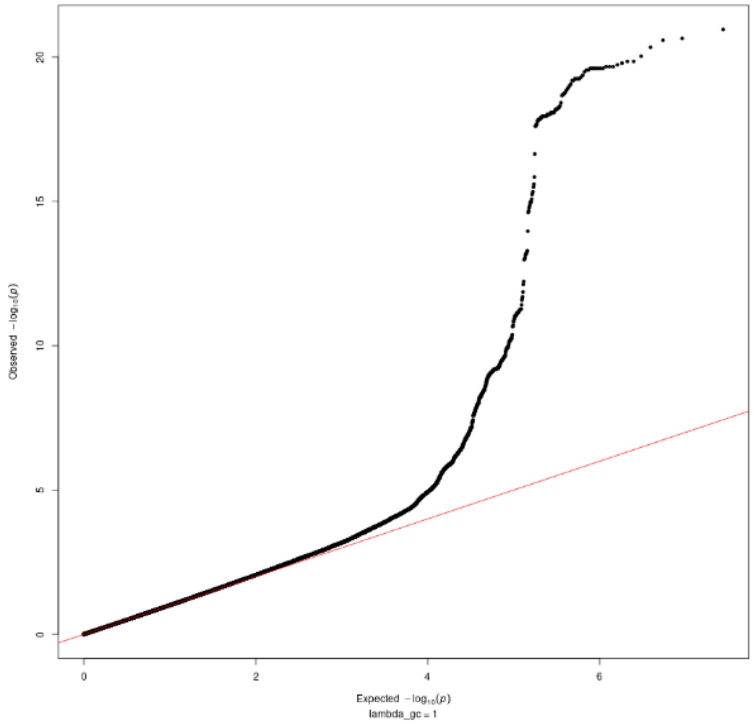

Supplementary Figure 1: Manhattan and QQ plots for European-only meta-analysis of Modified Stumvoll ISI adjusted for BMI

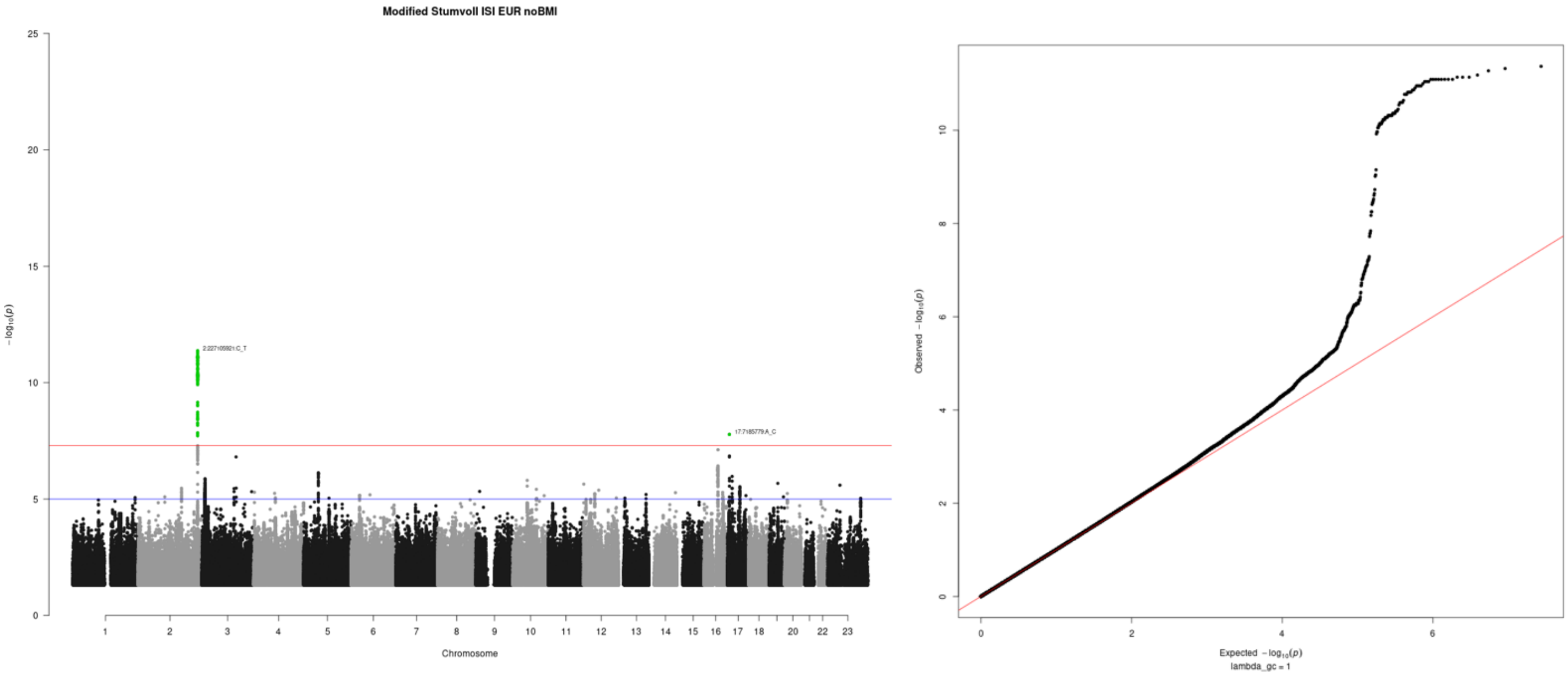

Supplementary Figure 2: Manhattan and QQ plots for European-only meta-analysis of Modified Stumvoll ISI unadjusted for BMI

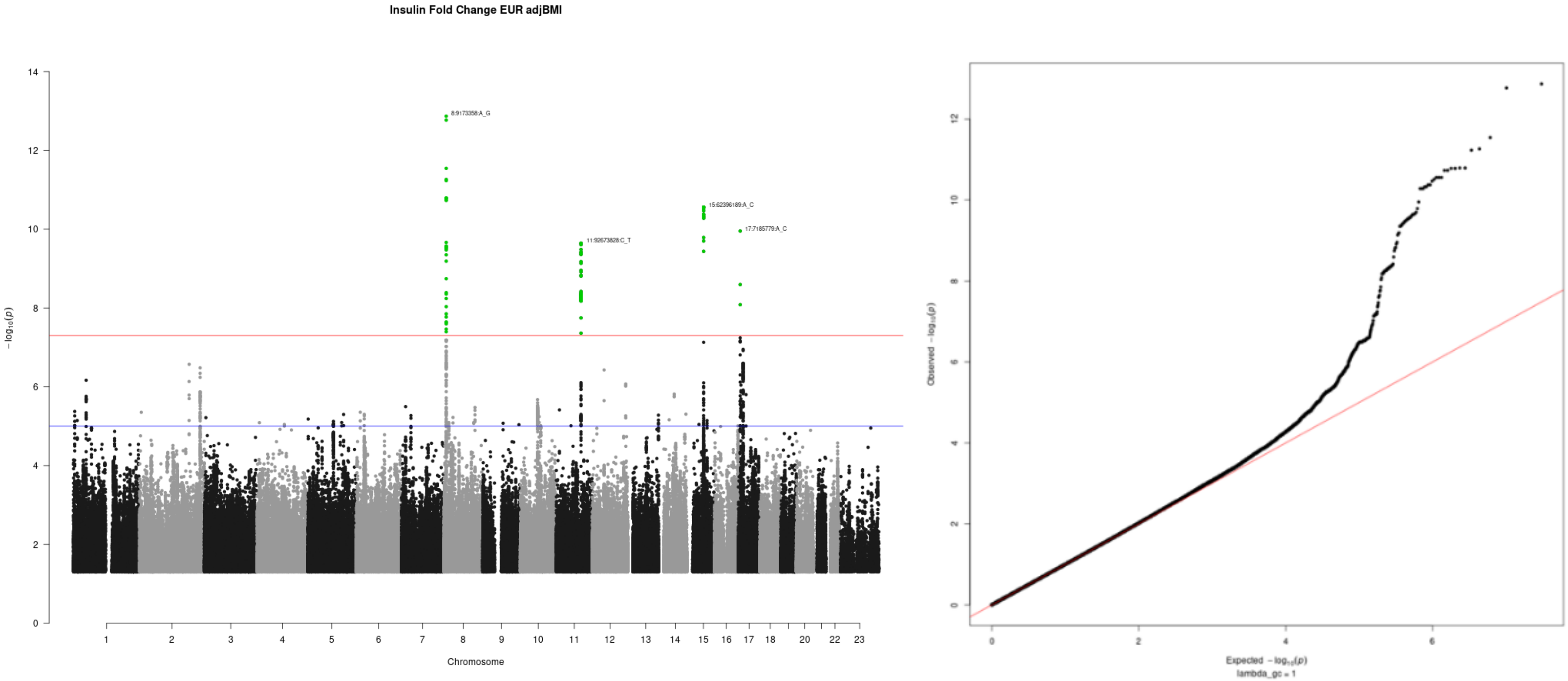

Supplementary Figure 3: Manhattan and QQ plots for European-only meta-analysis of insulin fold change adjusted for BMI

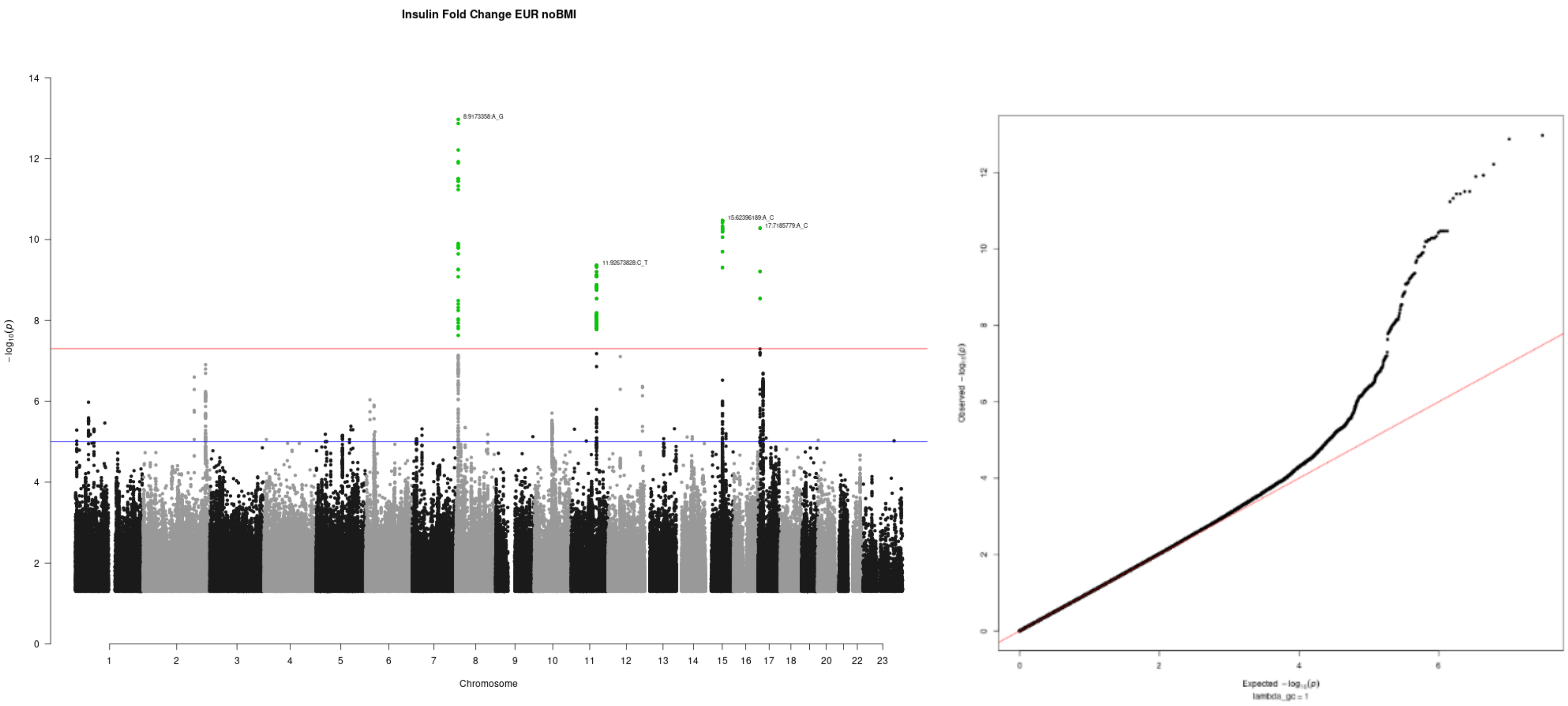

Supplementary Figure 4: Manhattan and QQ plots for European-only meta-analysis of insulin fold change unadjusted for BMI

3. Regional plots of genetic loci associated with Modified Stumvoll ISI adjBMI (Supplementary Figure 5)

Supplementary Figure 5: Regional association plots of genetic loci associated with Modified Stumvoll ISI adjBMI in European-only meta-analysis.

A locus was defined as ±500kb of the lead variant at a given locus. LD reference panel is 1000 Genomes CEU (European), the lead variant at a given locus is indicated by the purple triangle. Relative LD (R^2) of other variants with this lead variants is indicated by the colour scale shown in the legend on the left hand side of the plot. Unadjusted -log10 P-values are indicated on the y axis.

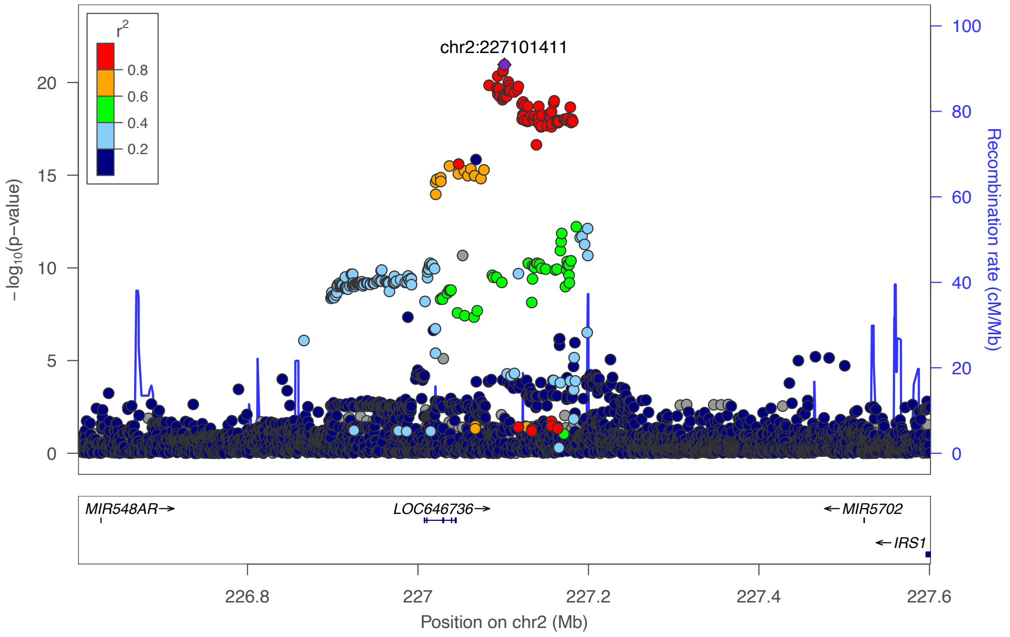

Supplementary Figure 5 a) IRS1

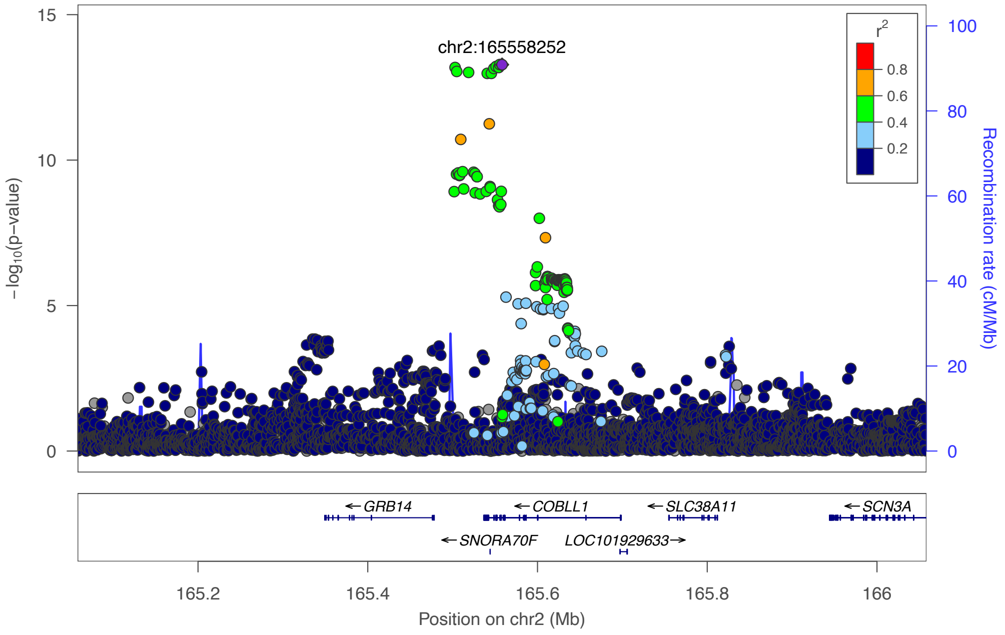

Supplementary Figure 5 b) COBLL1

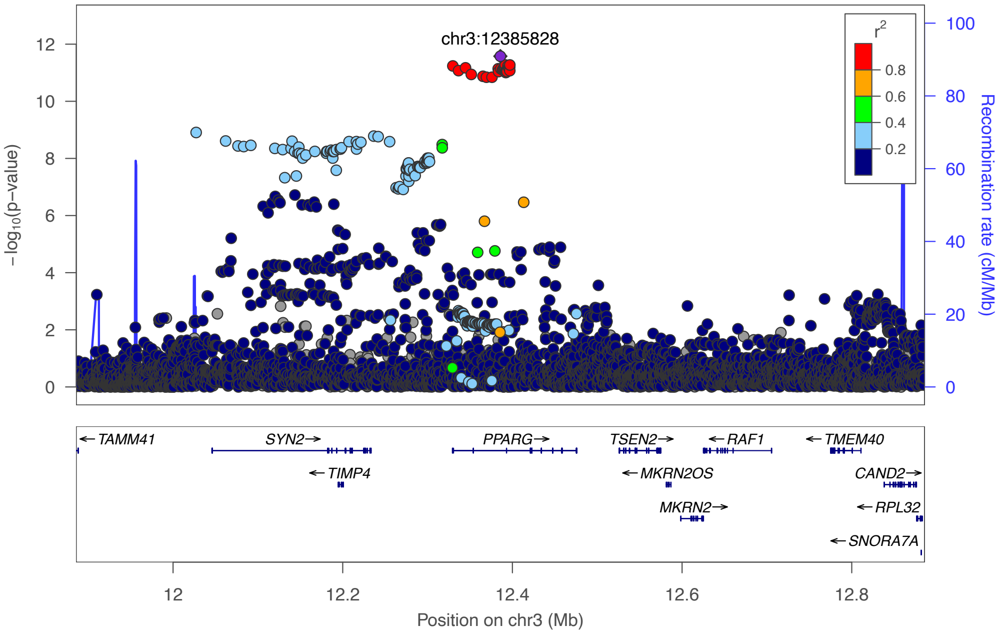

Supplementary Figure 5 c) PPARG

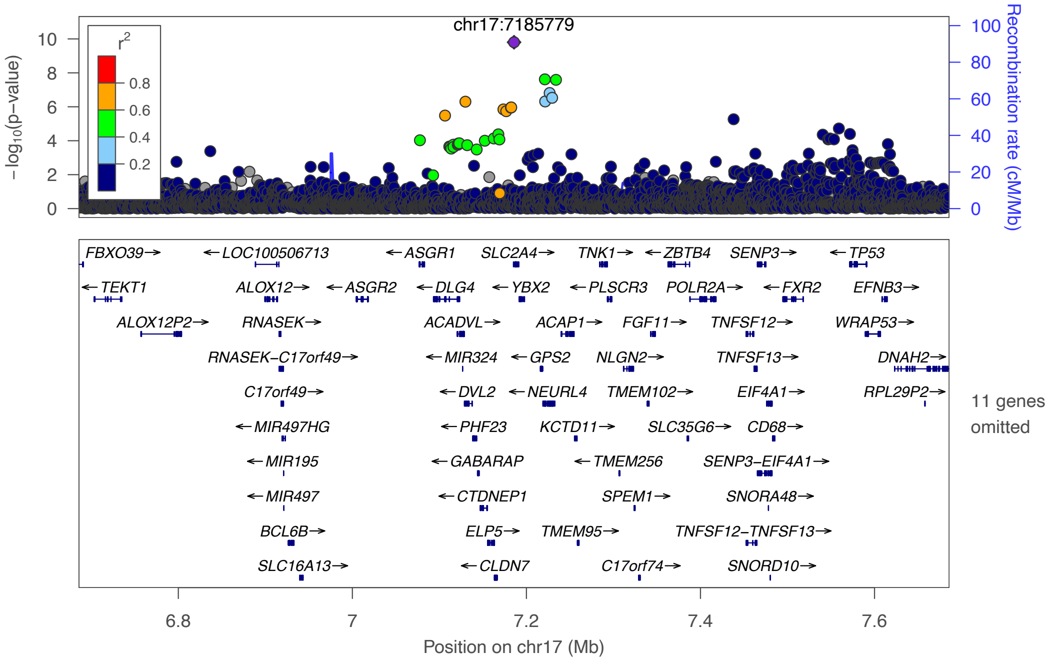

Supplementary Figure 5 d) SLC2A4

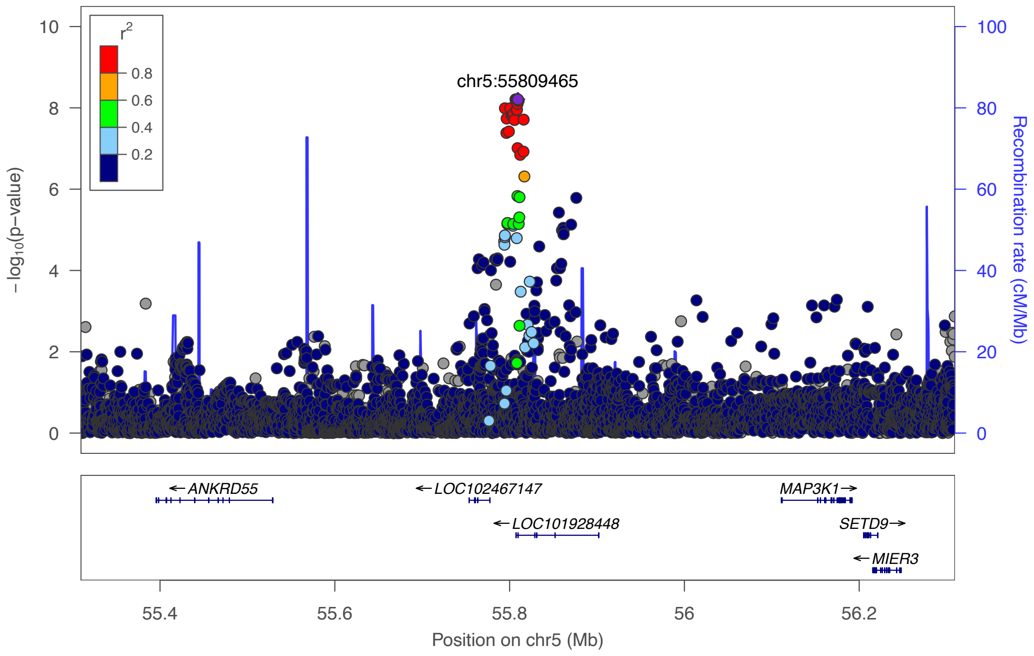

Supplementary Figure 5 e) C5orf67

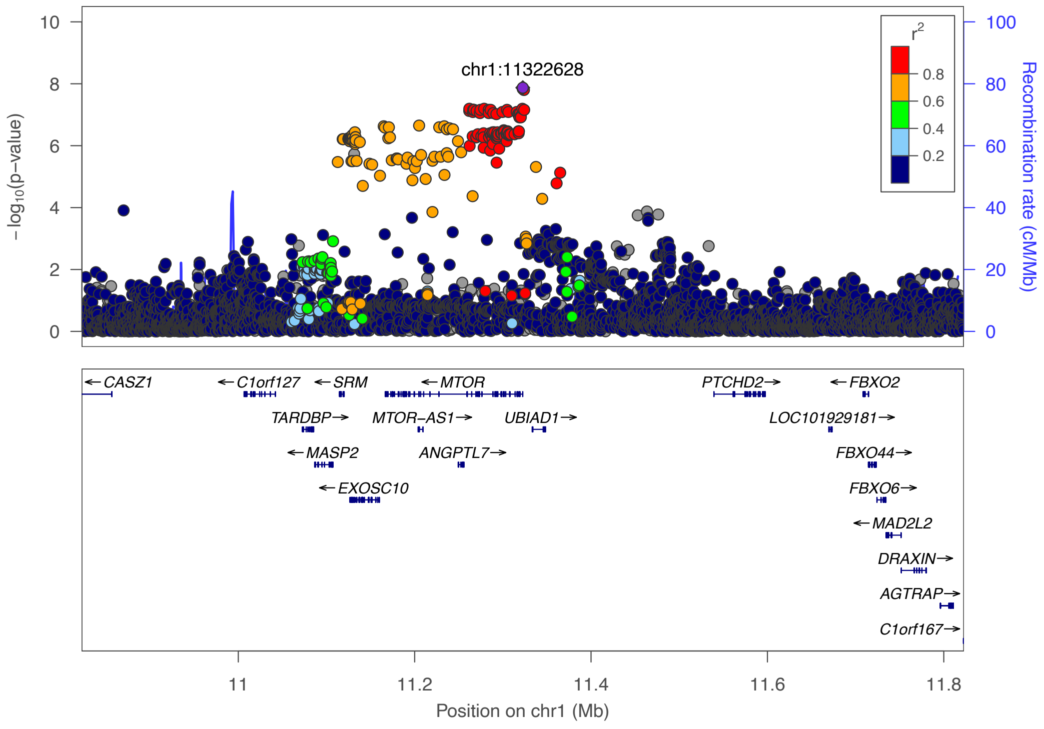

Supplementary Figure 5 f) MTOR

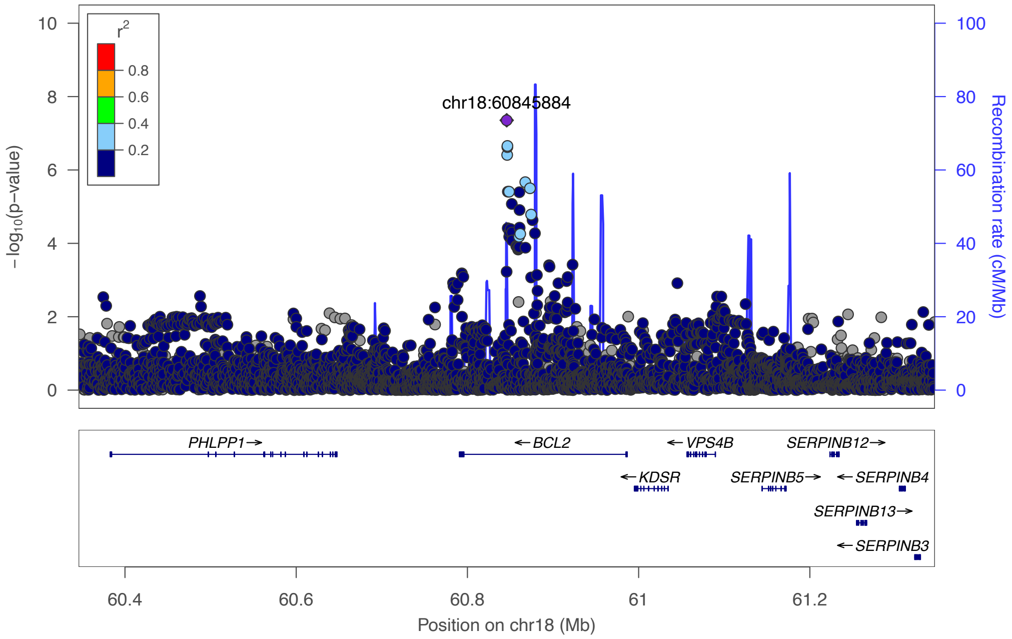

Supplementary Figure 5 g) BCL2

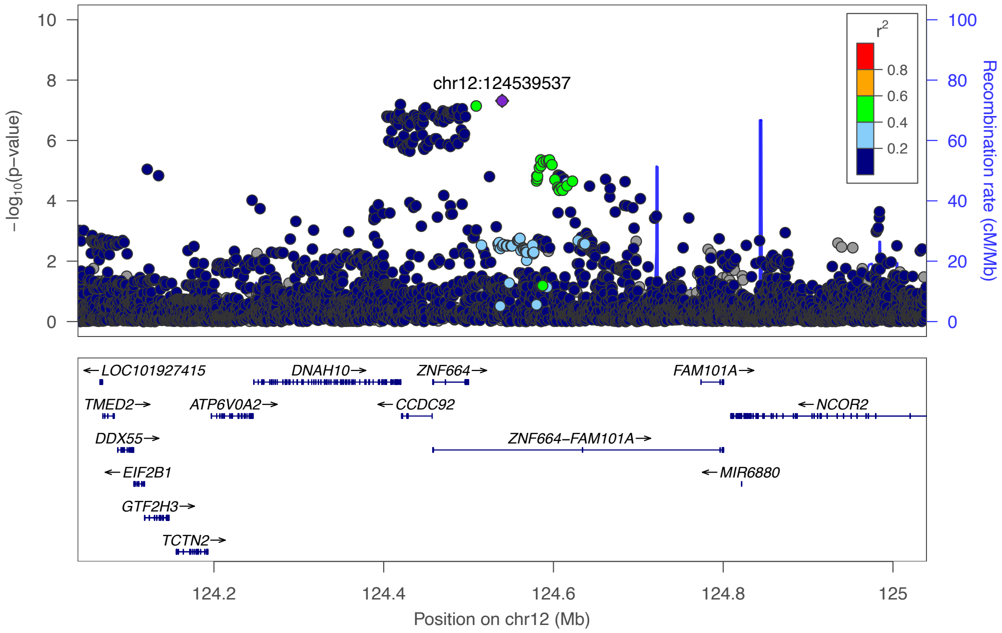

Supplementary Figure 5 h) FAM101A

4. Regional plots of genetic loci associated with Insulin Fold Change adjusted for BMI (Supplementary Figure 6)

Supplementary Figure 6: Regional association plots of genetic loci associated with insulin fold change adjBMI in European-only meta-analysis.

A locus was defined as ±500kb of the lead variant at a given, locus. LD reference panel is 1000 Genomes CEU (European), the lead variant at a given locus is indicated by the purple triangle. Relative LD (R^2) of other variants with this lead variants is indicated by the colour scale shown in the legend on the left hand side of the plot. Unadjusted -log10 P-values are indicated on the y axis. Unadjusted -log10 P-values are indicated on the y axis.

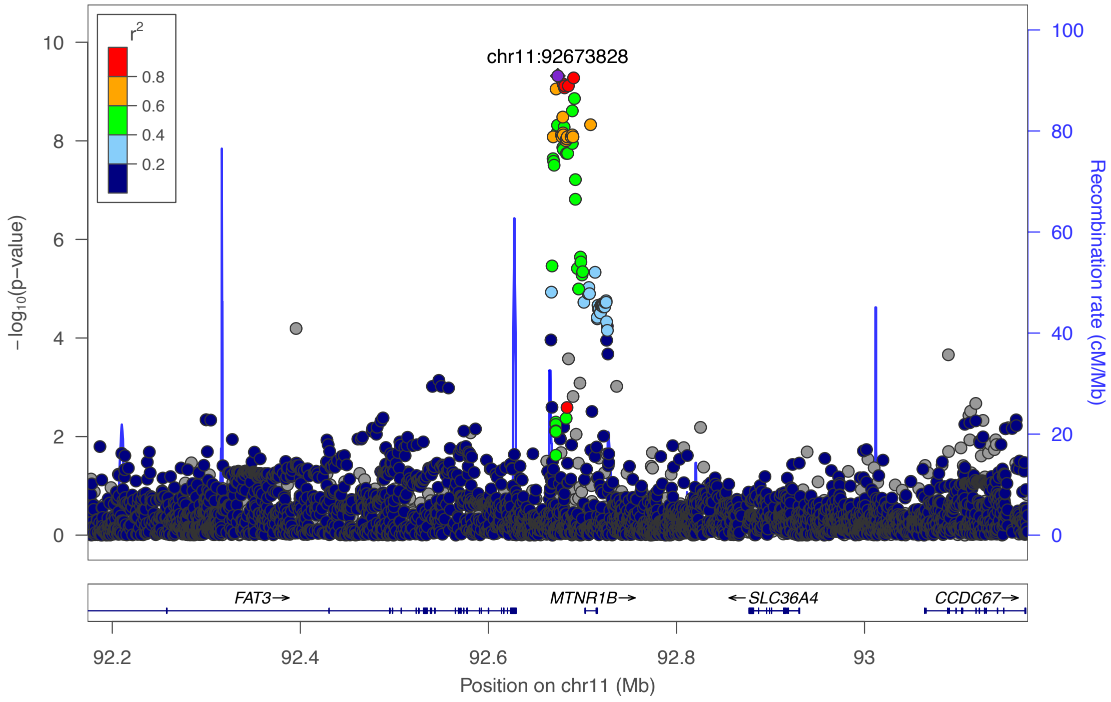

Supplementary Figure 6 a) MTN1RB

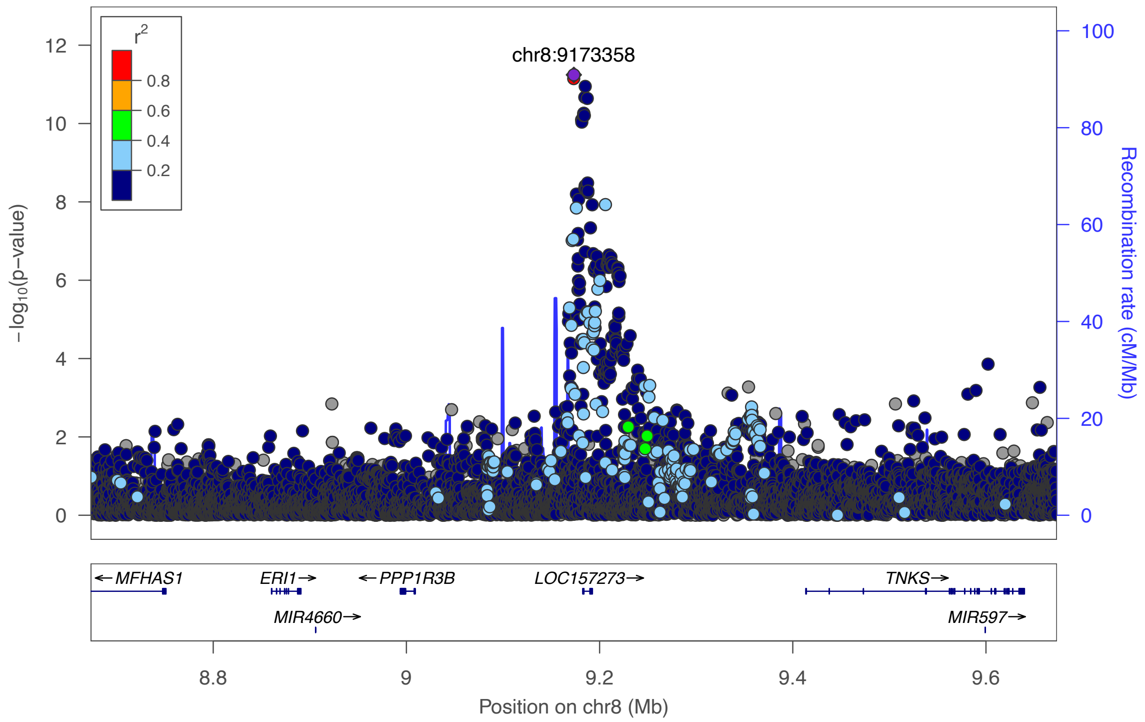

Supplementary Figure 6 b) PPP1R3B

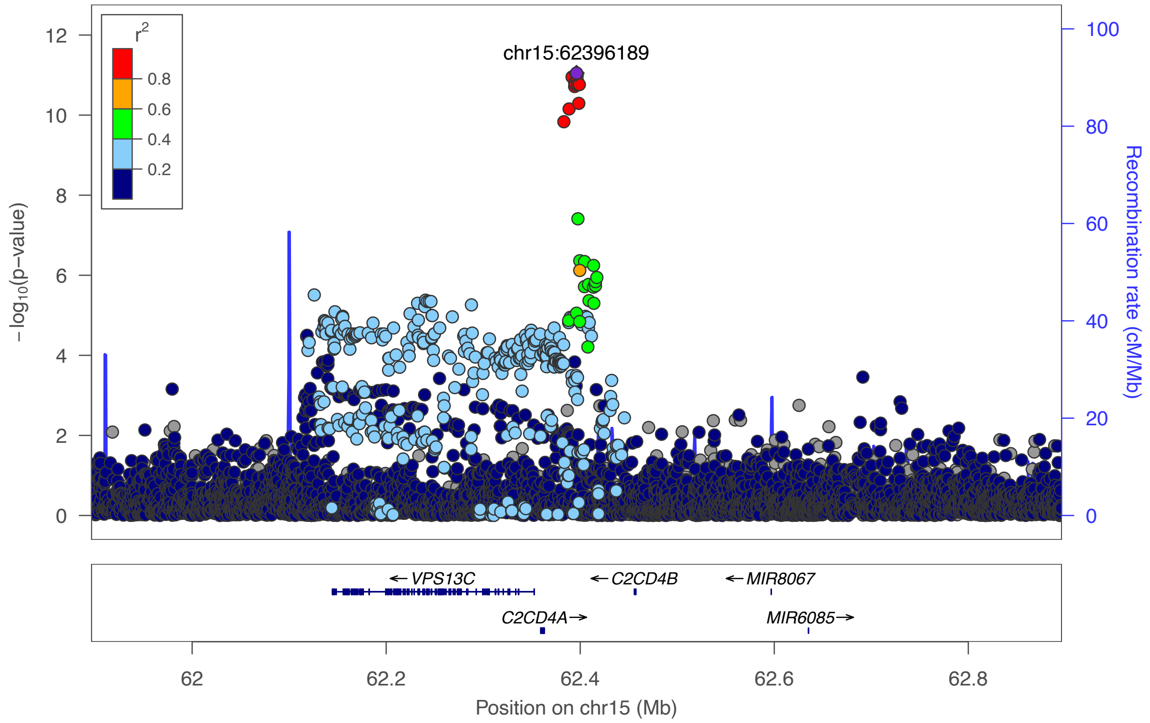

Supplementary Figure 6 c) C2CD4A

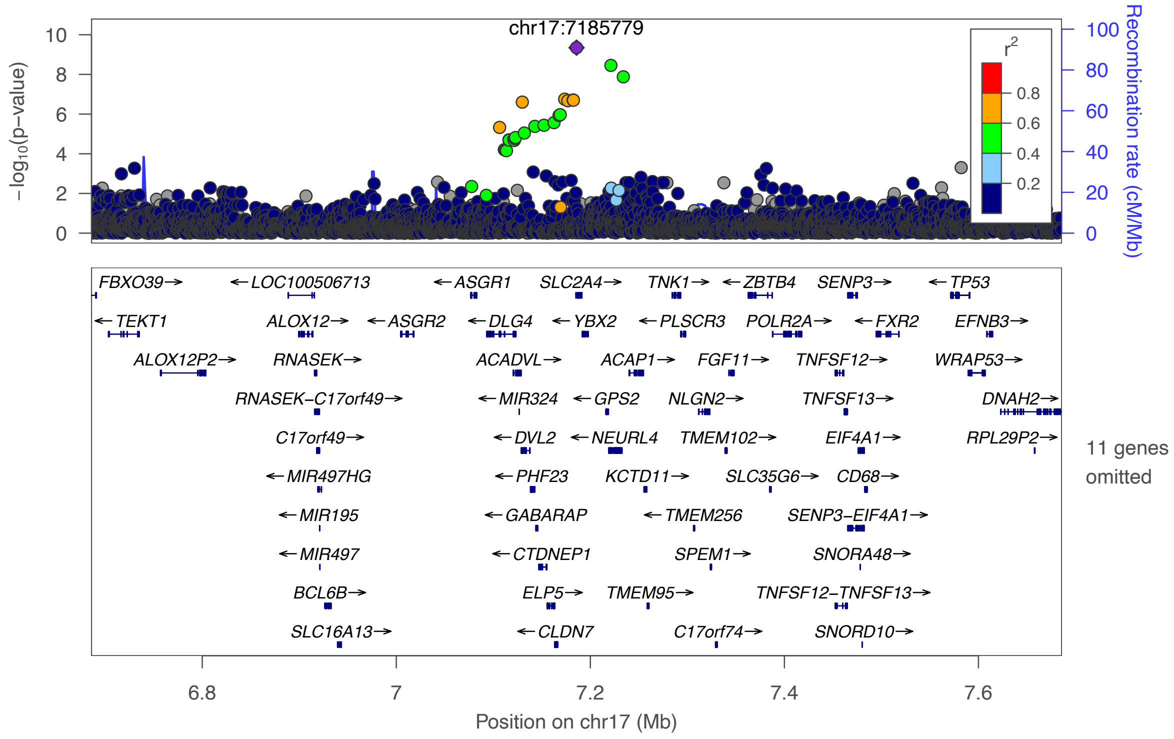

Supplementary Figure 6 d) SLC2A4

5. Genetic Discovery of post-challenge insulin resistance: non-European and multi-ancestry meta-analyses (Supplementary Figures 7-14)

We performed fixed-effect meta-analyses separately within cohorts of Hispanic American ancestry using METAL[14] for IFC and ISI. To examine trans-ancestry effects we conducted random effects meta-analyses of these ancestry-specific results (EUR, EAS, HISAMR) using METAL (**See methods**). Manhattan and QQ plots summarising the results can be found in **Supplementary Figures 7-14** below. Unadjusted -log10 P-values are indicated on the y axis of the Manhattan plots. QQ plots show the observed (Y) vs expected (X) -log10 P value. Unadjusted P values are shown. In Manhattan plots on the left, the green points indicate those that meet genome wide significance. Red line indicates genomewide significance (P < 5e-8)_ and blue line represents suggestive significance (P < 1e-5).

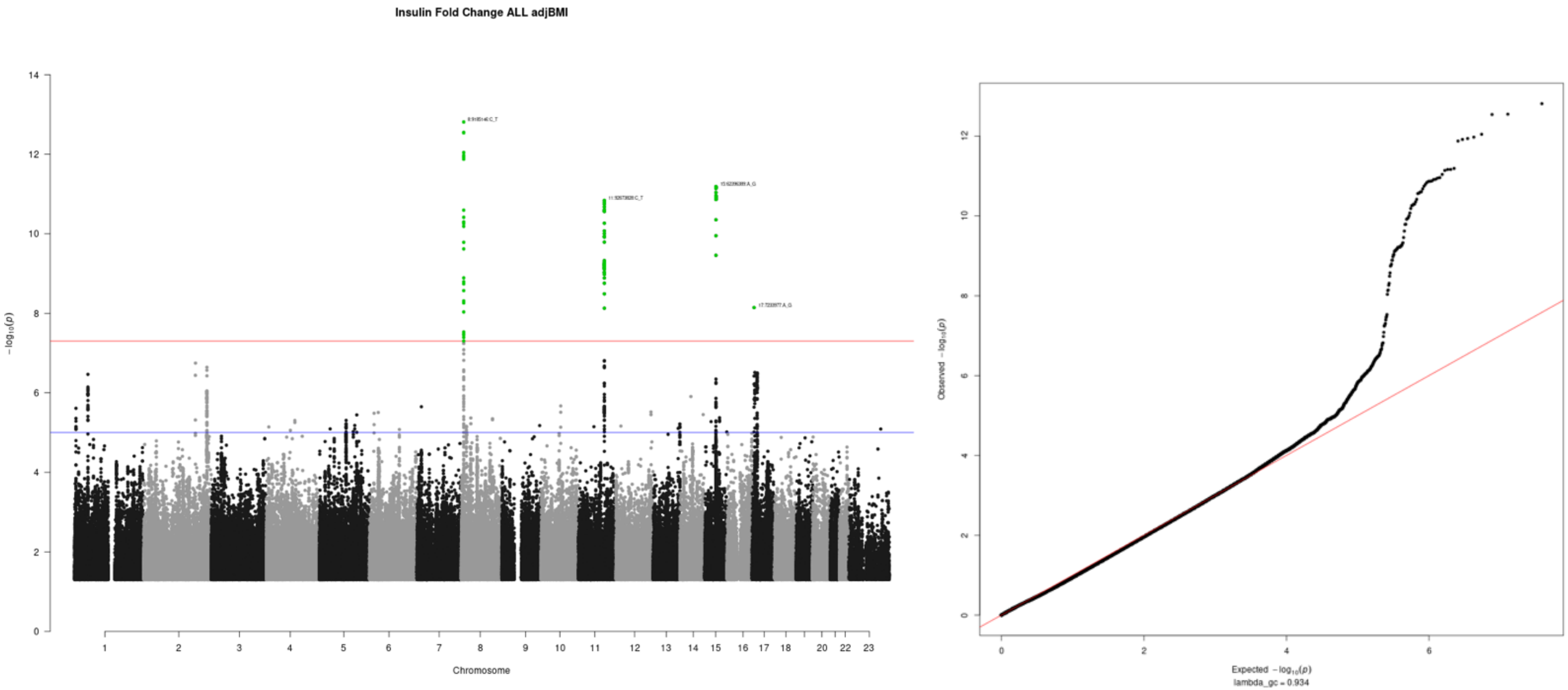

Supplementary Figure 7: Manhattan and QQ plots for multi-ancestry (EUR, HISAMR, EAS) meta-analysis Insulin Fold Change adjusted for BMI

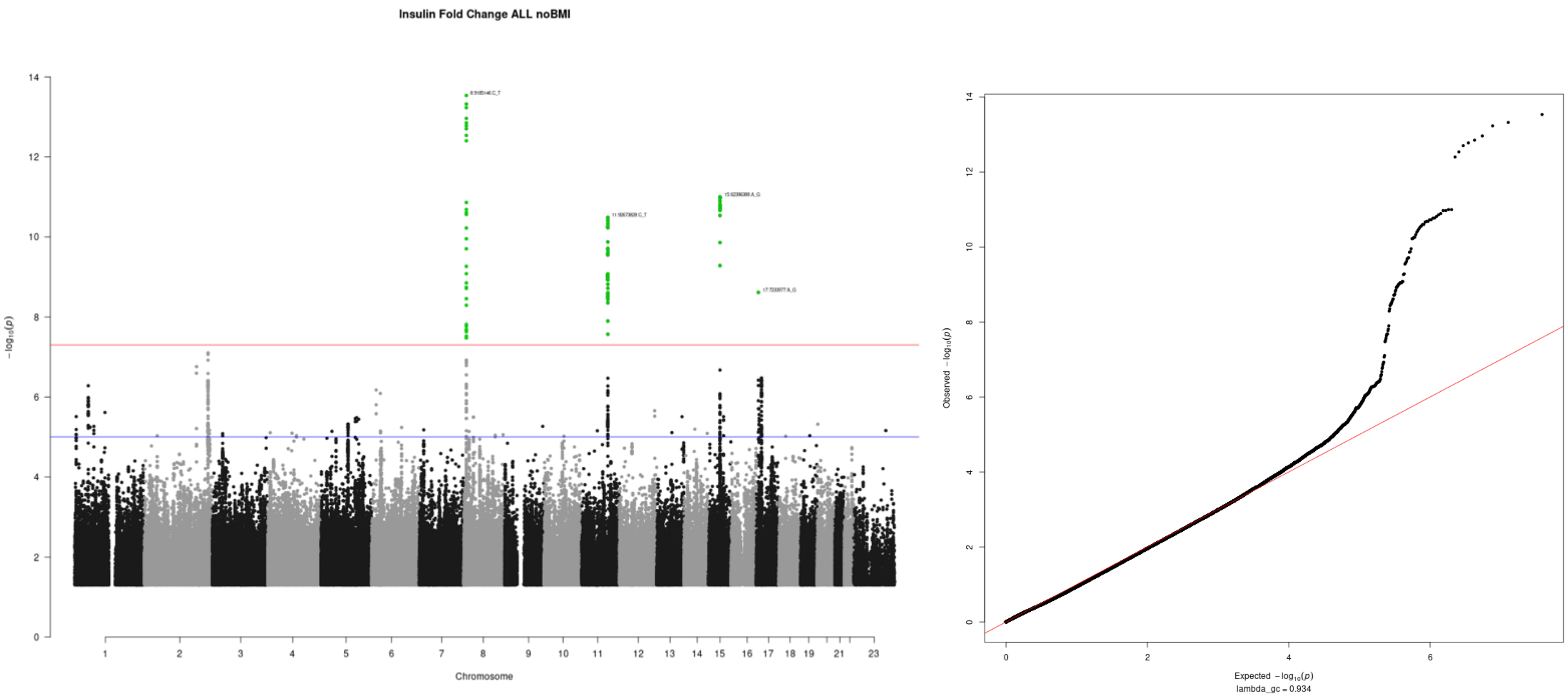

Supplementary Figure 8: Manhattan and QQ plots for multi-ancestry (EUR, HISAMR, EAS) meta-analysis Insulin Fold Change unadjusted for BMI

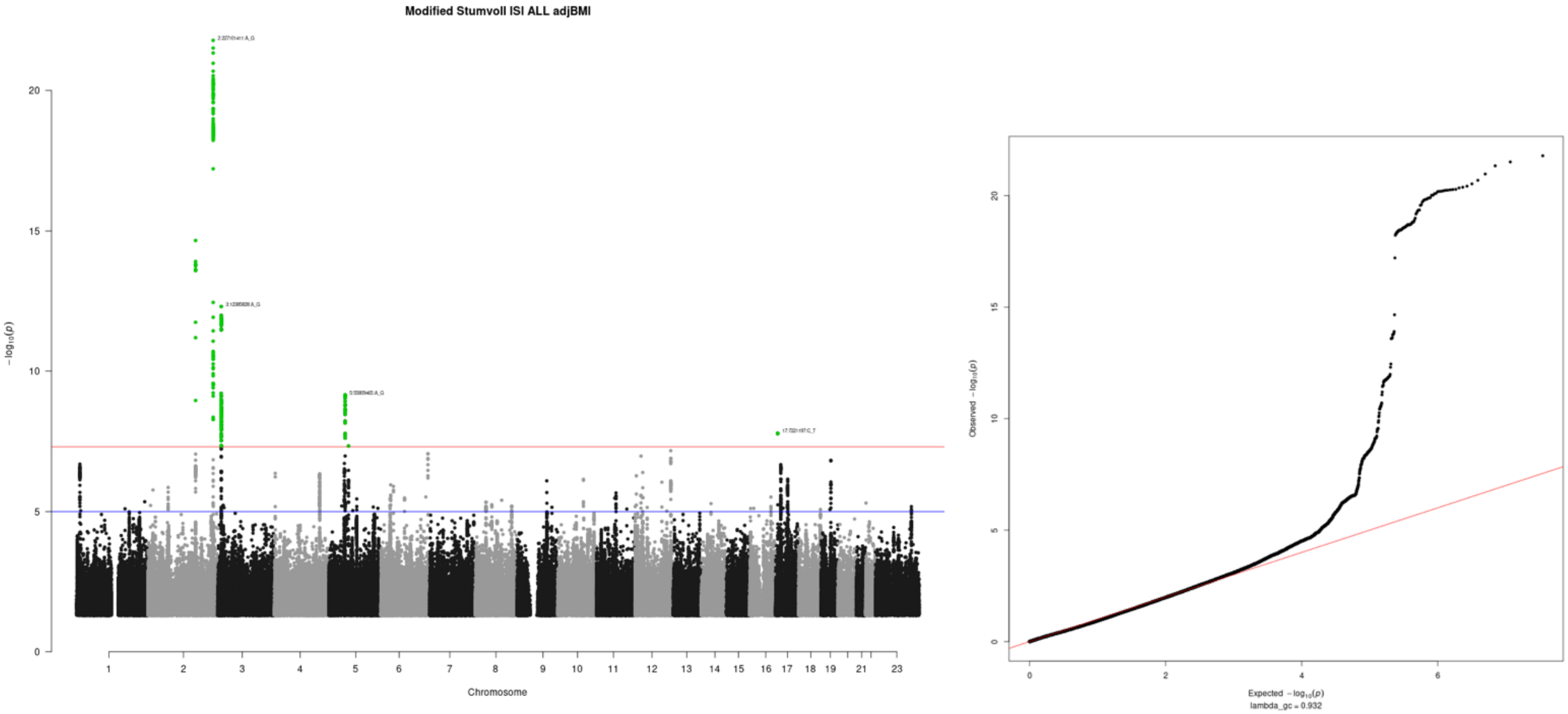

Supplementary Figure 9: Manhattan and QQ plots for multi-ancestry (EUR, HISAMR, EAS) meta-analysis Modified Stumvoll ISI adjusted for BMI

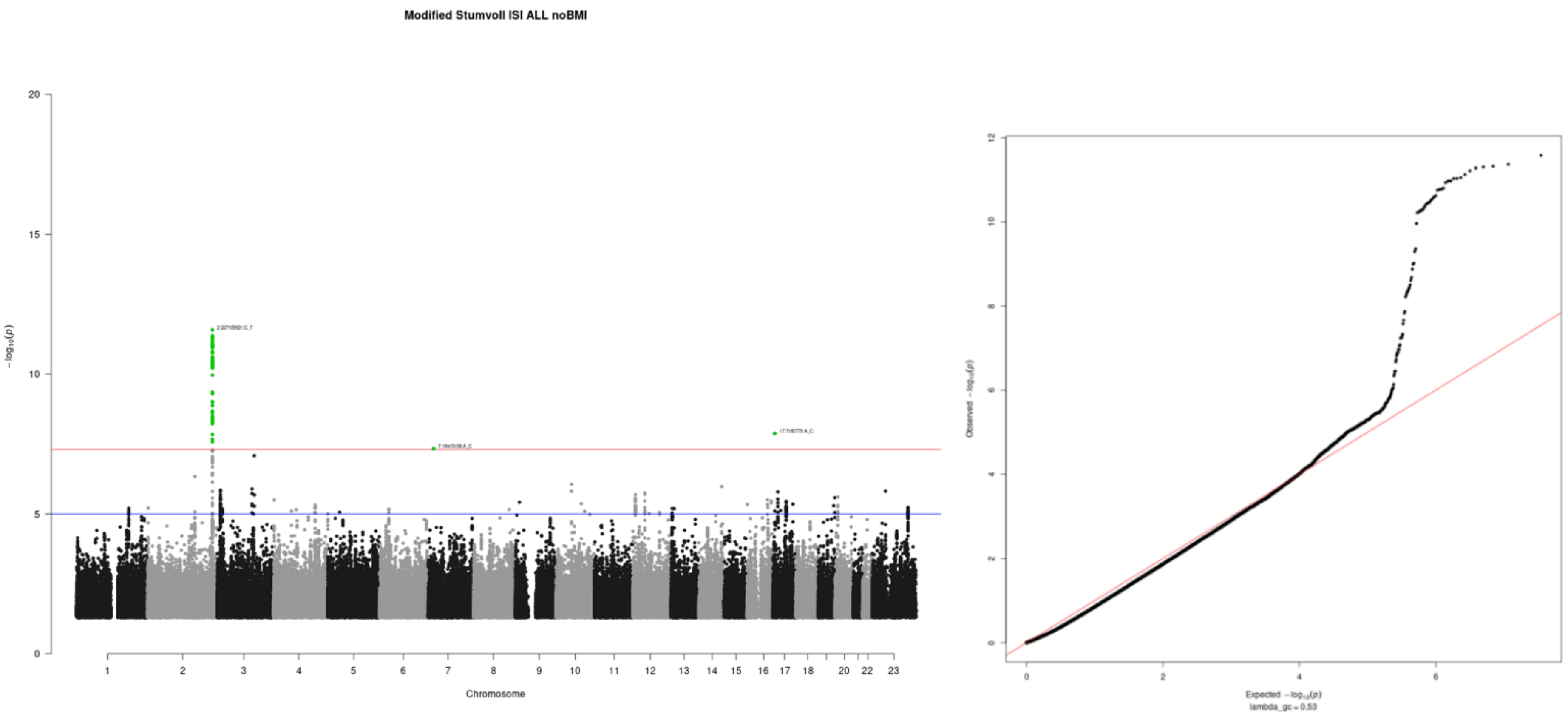

Supplementary Figure 10: Manhattan and QQ plots for multi-ancestry (EUR, HISAMR, EAS) meta-analysis Modified Stumvoll ISI adjusted for BMI

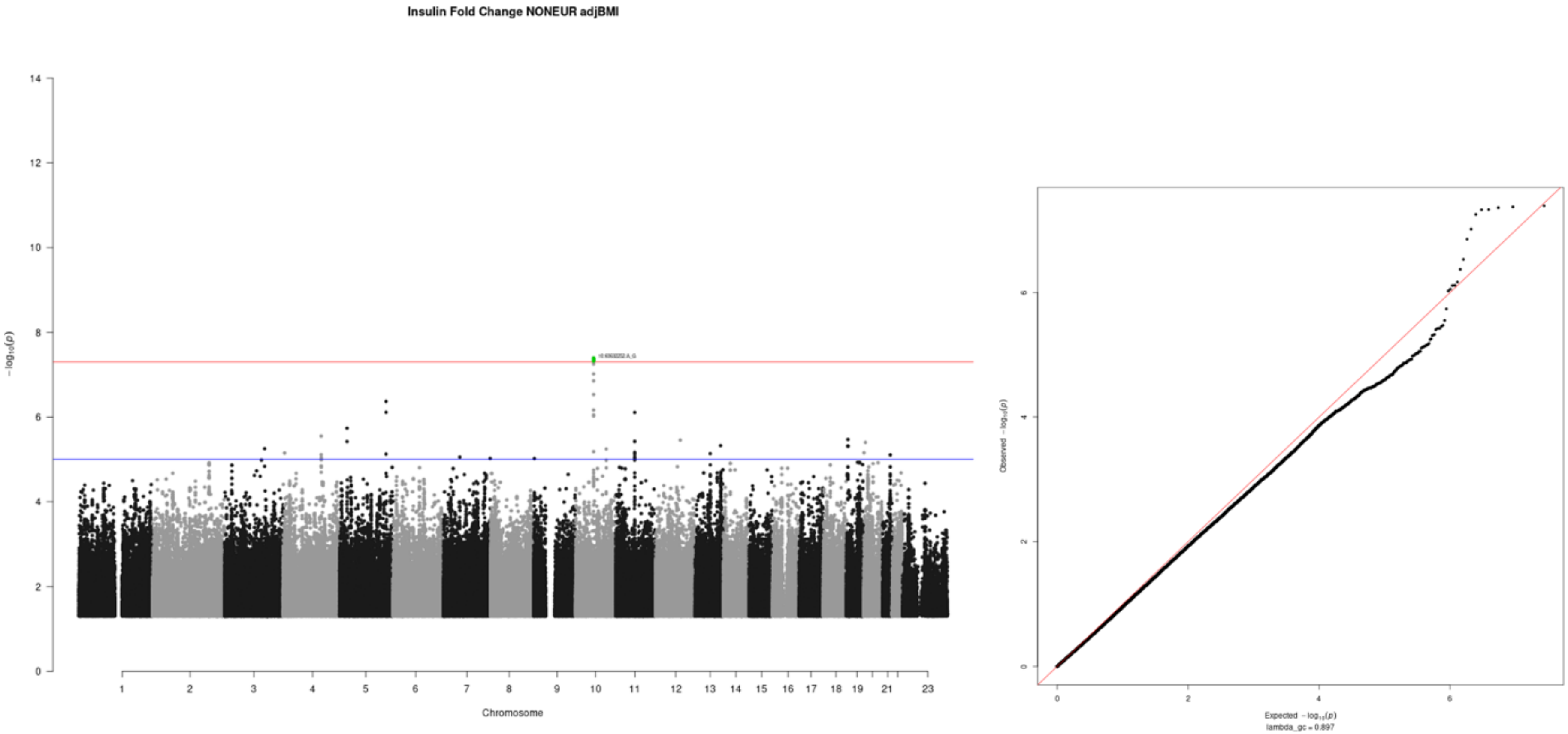

Supplementary Figure 11: Manhattan and QQ plots for non-European Ancestry (HISAMR, EAS) meta-analysis Insulin Fold Change adjusted for BMI

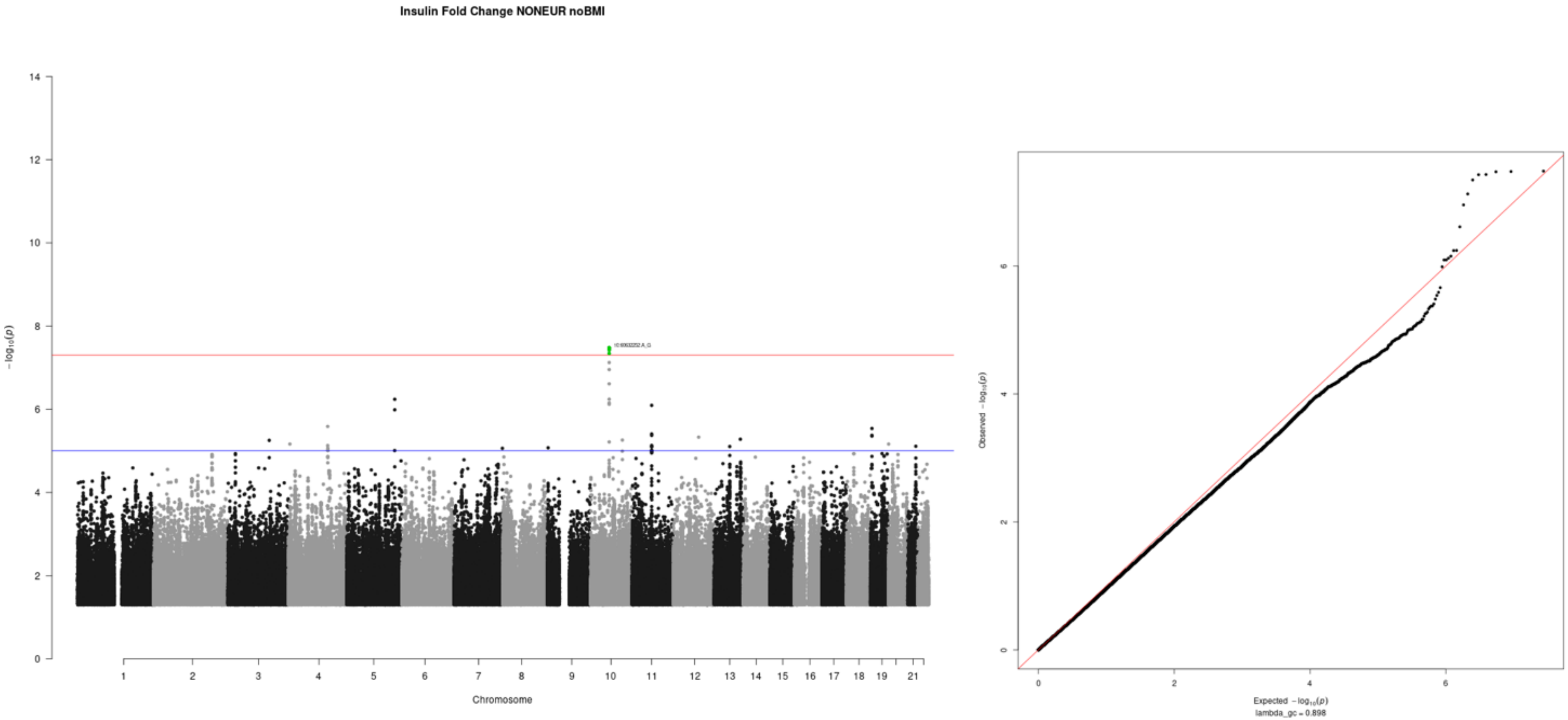

Supplementary Figure 12: Manhattan and QQ plots for non-European Ancestry (HISAMR, EAS) meta-analysis Insulin Fold Change unadjusted for BMI

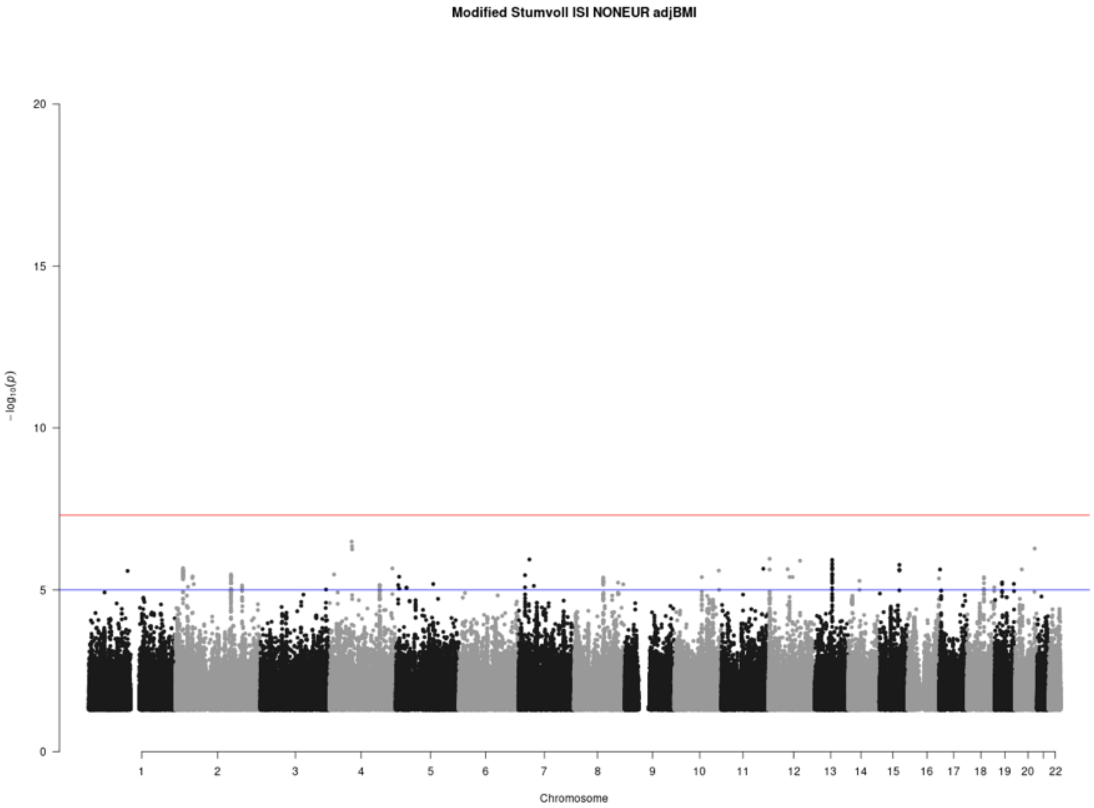

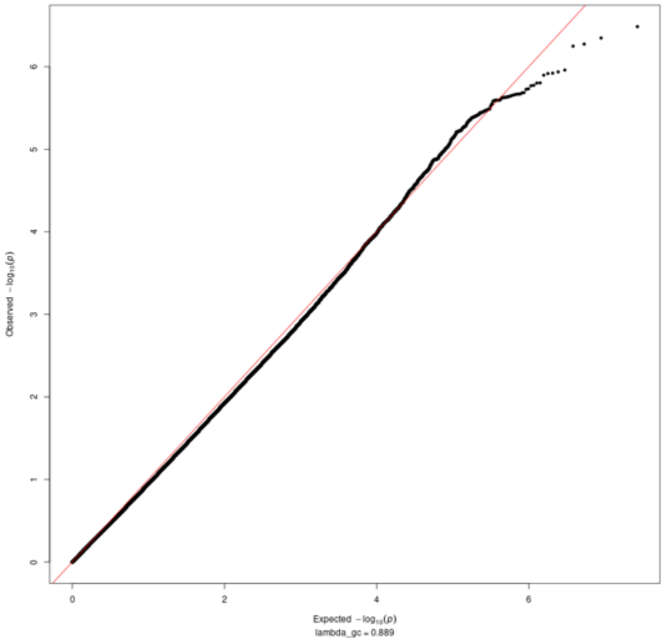

Supplementary Figure 13: Manhattan and QQ plots for non-European Ancestry (HISAMR, EAS) meta-analysis Modified Stumvoll ISI adjusted for BMI

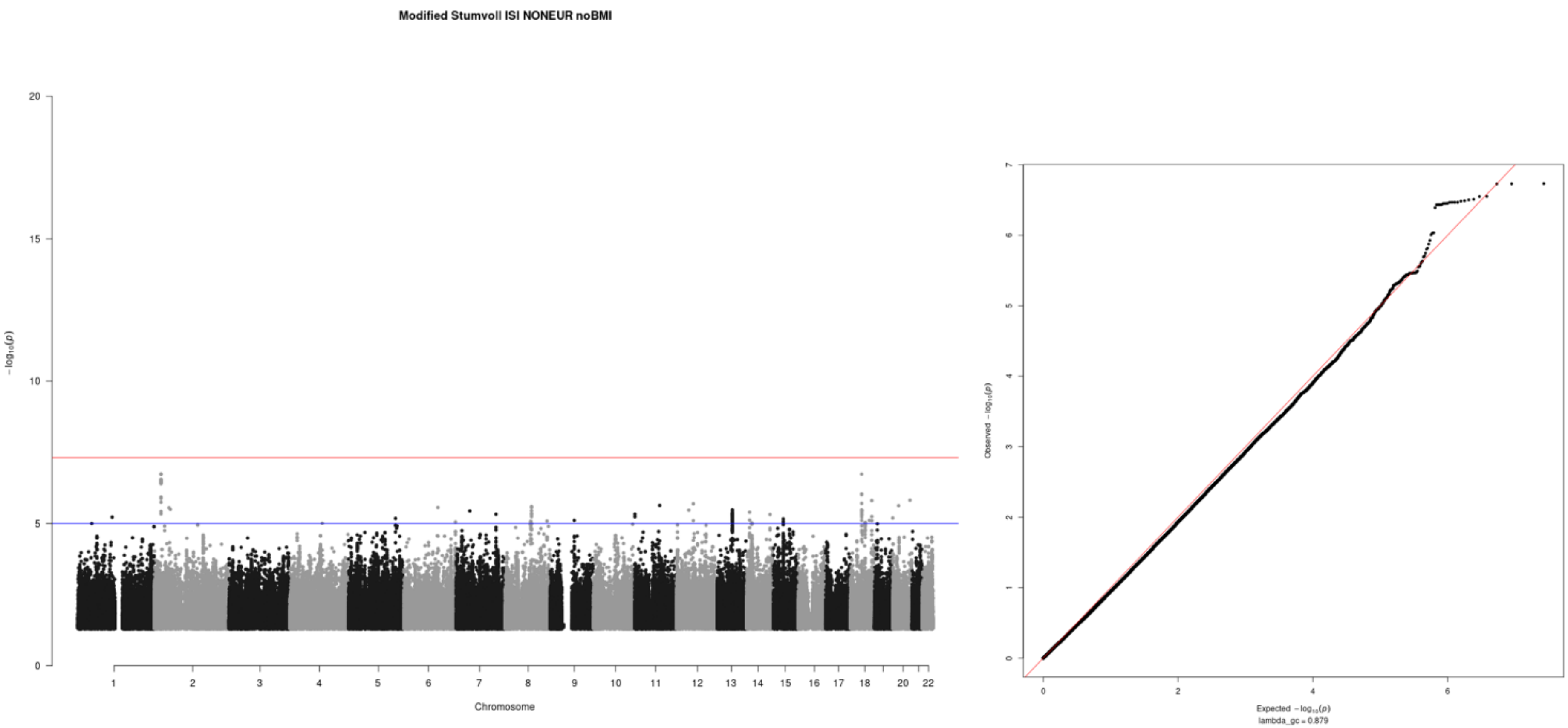

Supplementary Figure 14: Manhattan and QQ plots for non-European Ancestry (HISAMR, EAS) meta-analysis Modified Stumvoll ISI unadjusted for BMI

6. rs60453193 (chr10:60632252_A_G (b37)) at *BICC1* is associated with Insulin Fold Change specifically in Non-European Cohorts

We additionally identified *BICC1* (rs60453193, beta = 0.43, SE = 0.08, P = 4.06×10^{-8}, N = 1,837) as a signal associated with IFC **(see Methods).** This locus has not been implicated in post-challenge insulin resistance previously. The effects were consistent in meta-analyses of only East Asian (beta = 0.406, SE = 0.204, P = 0.046, N = 739) and Hispanic American ancestry studies (beta = 0.435, SE = 0.085, P = 3.15×10^{-7}, N = 1,098) (**Supplementary Tables 6 and 7; Extended Data 5-6**). *BICC1* was not associated with IFC in meta-analysis of studies only of European ancestry (beta = -0.0026, SE = 0.010, P = 0.791, N = 50,671; **Supplementary Table 6**); despite being common in all ancestry groups (MAF European = 12%, Hispanic American =7.8%, East Asian = 4.2%). Further, *BICC1* was not statistically significant in the multi-ancestry analyses of IFC, including all cohorts (beta = 0.26, SE = 0.18, P = 0.16, N = 52,508; **Supplementary Tables 6 and 7**), likely due to cohorts of European ancestry dominating the overall sample size (97%). Variants within this locus (±500kb of rs60453193) have been reported to be associated with T2D at suggestive significance in multi-ancestry analyses[8] (Minimum P = $1.0 \times 10^{-4.7}$), however rs60453193 itself is not associated (MVP multi-ancestry T2D: beta = 0.0079, SE = 0.0062, P = 0.020) [8]. Rare, damaging variants in *BICC1* have been reported to be nominally associated with T2D in individuals of European ancestry.[15]

7. SLC2A4 is in perfect D' with variants reported to be associated with type 2 diabetes in Hispanic American and East Asian ancestries.

SLC2A4 locus and T2D risk

The lack of evidence of a shared signal between post-challenge insulin resistance and T2D at SLC2A4 in cohorts of European ancestry is surprising, since lead variant rs117643180 is located in the first intron of *SLC2A4*, encoding GLUT4, a key player in post-prandial glucose uptake. rs117643180 is also associated with higher 2 h glucose (**Supplementary Table 6**), one of the diagnostic criteria for T2D[7,16]. There are several reasons which may explain this. First the true causal variant for T2D may not be captured. *SLC2A4* lead variant rs117643180 is in perfect linkage disequilibrium (LD; D' = 1) with lead SNPs at T2D loci identified in East Asian and Hispanic American ancestries (**Supplementary Figures 15- 17, Supplementary Table 18**).[17,18] The respective lead variants at this locus in each ancestry are too rare in other ancestries, such as European descent, to be included in previous efforts (**Supplementary Table 18)**[19]. The small sample size of non-European ancestries for post-challenge insulin resistance traits, unfortunately does not allow formal statistical colocalisation testing.
Finally, it is possible that epistatic effects may play a role and the A risk allele of rs117643180 for post-challenge insulin resistance co-segregates with the established nearby protective T2D C allele of rs858519 (T2D association at rs858519: Beta = -0.025, SE = 0.004, P = 1.28e-9 , EA = C EAF = 0.505)[8]. For instance in 1000 genomes[20], the rs117643180-A risk allele is present at a frequency of 3.1% in European ancestry, and when present, this co-segregates on the same haplotype with the T2D protective C allele at rs858519 approximately 42% of the time[21].

Finally our analyses focus on variants with MAF > 0.5% and there is suggestive evidence of rare predicted damaging missense variants (**see methods**) in *SLC2A4* being associated with T2D (MAF < 0.005%, N SNPs = 68) in ~450,000 European ancestry participants from the UK Biobank with whole exome sequencing[15]. These variants have been reported to be nominally associated with increased diabetes risk (UK Biobank field 20002 #1220, OR = 1.79 [1.25-2.68], P = $5.5x10^{-3}$), increased random glucose (Beta = 0.11, SE = 0.05, P =0.03) and increased HbA1c (Beta = 0.13, SE = 0.06, P = 0.018).[15]

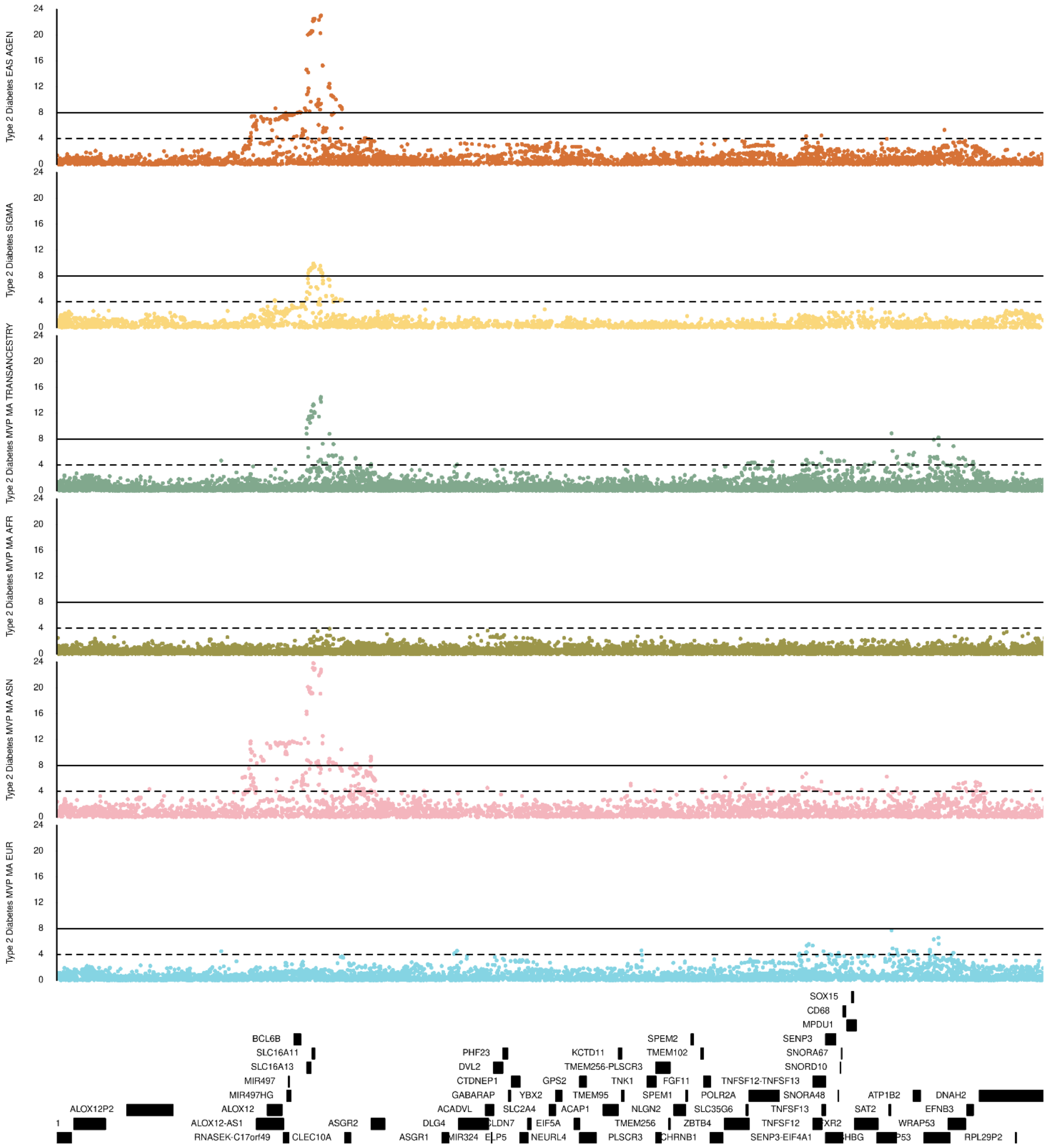

Supplementary Figure 15: SLC2A4 locus regional association plot for Type 2 Diabetes across studies in different ancestry groups.

Locus is defined as ±500kb from lead ISI and IFC SNP rs117643180. From top to bottom the tracks denote the following publicly available Type 2 Diabetes meta-analysis results: Track 1: East Asian Ancestry (AGEN; Spracklen et al; 2021)[17]. Track 2: Hispanic American ancestry, SIGMA consortium (Wiliams et al 2013)[18]. Track 3: MVP meta-analysis (Vujkovic et al; 2020)[8] trans-ancestry. Track 4: MVP meta-analysis (Vujkovic et al; 2020) African ancestry. Track 5: MVP meta-analysis (Vujkovic et al; 2020) Asian ancestry. Track 6: MVP meta-analysis (Vujkovic et al; 2020) European ancestry. Track 7: genes located at this locus. Y axis represents the –log10(p-value) of association. (unadjusted p-value). The solid line refers to genome-wide significance threshold of P = 5e-8, and the dashed line denotes to suggestive significance threshold of p= 1e-5.

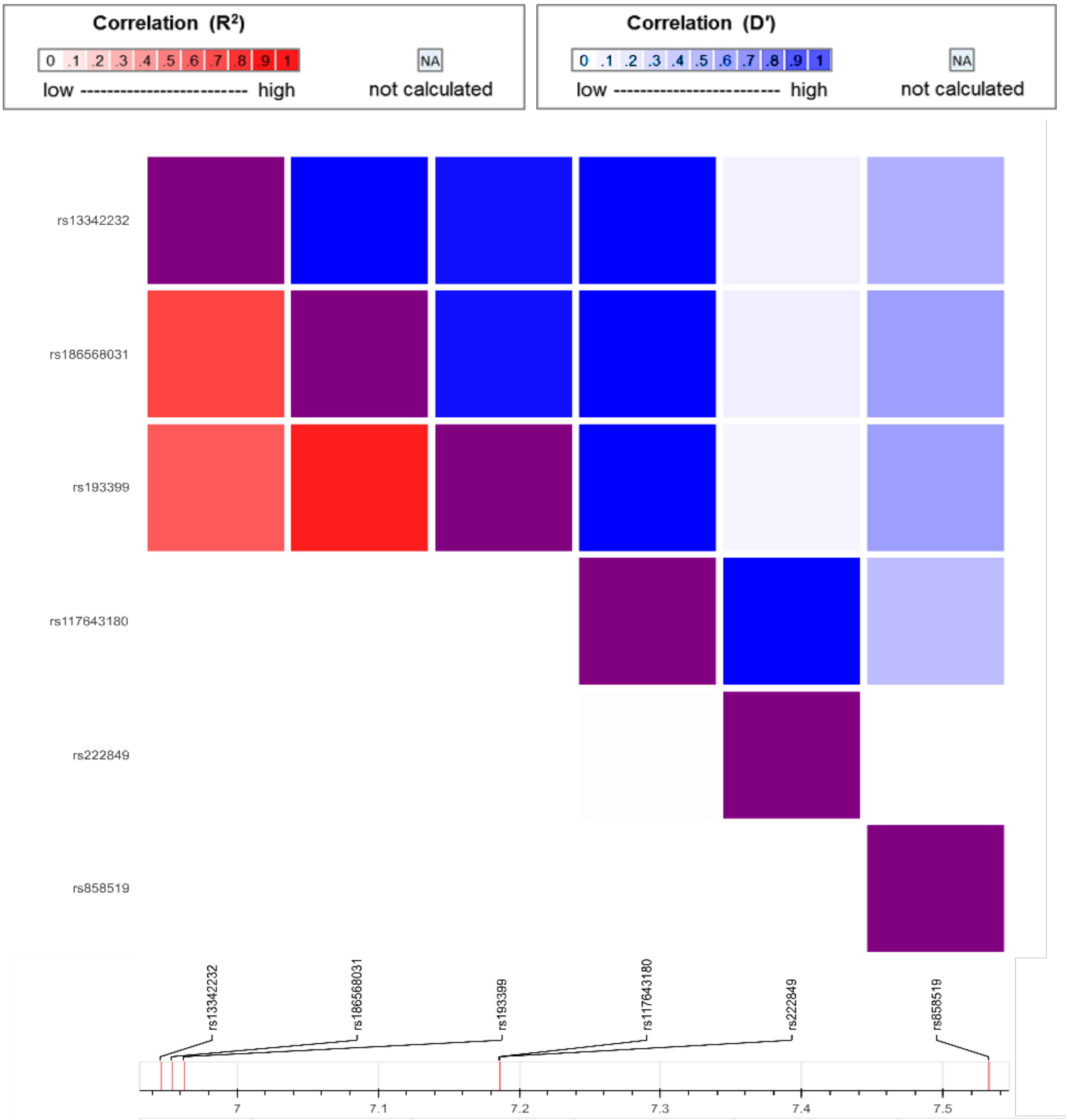

Supplementary Figure 16: LD matrix for lead SNPs in IFC, ISI and T2D GWAS at SLC2A4 locus in European ancestry

LD matrix generated for individuals of European ancestry using LDLink[21]. LD as denoted by R^2 is shown in red, and D' in blue. The colour scales are indicated at the top of the plot.

8. Genetic associations of IFC and ISI with other cardiometabolic traits

We further assessed the associations of increased genetic risk for post-challenge insulin resistance, represented by genetic risk scores (GRS; **see methods**) for ISI and IFC, with T2D related traits in unrelated European ancestry participants in UK Biobank (UKBB). We identified highly significant associations between both the ISI and IFC GRS with type 2 diabetes, with directions of associations consistent with post-challenge insulin resistance and increased risk of T2D (ISIadjBMI: beta = -0.28, SE = 0.020, P = 1.97×10^{-43}; IFCadjBMI: beta = 0.17, SE = 0.027, P = 1.40×10^{-10}). Directionally consistent effects were also seen for GRS for ISI and IFC with HbA1C, random glucose, WHR adjusted for BMI, which were all increased with increased genetic risk of post-challenge insulin resistance (**Supplementary Table 13**). We further assessed the associations between GRS for ISI and IFC and glycaemic traits not available in UKBB in a subset of the Fenland Study. In this much smaller study (N max = 8,925), the IFC and ISI GRS were still nominally (P < 0.5) associated with dynamic glycaemic traits and with associations being directionally consistent with post-challenge insulin resistance (**Supplementary Table 14**).

However, it should be noted that the Fenland Study is 1 of 28 studies that contributed to the GWAS meta-analyses from which these scores were constructed, reflecting 19% of the total sample size included in the discovery.

We used LD score regression analyses to assess genome-wide genetic correlations of ISI and IFC with a wider range of related biochemical and cardiometabolic traits. We identified strong genetic correlations of both ISI and IFC with 2h glucose, consistent with insulin resistance and the observational correlations in the Fenland Study (IFC and 2 h glucose: genetic correlation (rg) = 0.713, SE = 0.089, P = 2.63×10^{-22}; ISI and 2 h glucose: rg = -0.820, SE = 0.048, P = 2.12×10^{-22}; **Supplementary Tables 4, 15 and 16**). In contrast to ISI, which was strongly correlated with fasting insulin levels, IFC showed only a weak correlation, highlighting greater specificity as an indicator of post-challenge insulin resistance. Both ISI and IFC showed only moderate genetic correlations with T2D, consistent with the fact that multiple pathways contribute to its genetic architecture, with postprandial insulin resistance being only one of several aetiologies (**Supplementary Table 15 and 16**)

9. Colocalisation of post-challenge insulin resistance and eQTL for SLC2A4 in skeletal muscle at the SLC2A4 locus

The *SLC2A4* locus was the only locus where we did not see evidence of colocalisation of IFC/ISI with marginal statistics for the identified eQTL signal. This is due to two independent eQTL signals in skeletal muscle having been identified for *SLC2A4* at the *SLC2A4* locus (rs117643180)[22]. We ran conditional analyses using GCTA-cojo to generate conditional summary statistics conditioning on the primary (rs117643180) and secondary (rs222849) signals at this locus. Genetic of IFC/ISI and this eQTL signal were identified only when conditioning on the secondary signal (rs222849) showing that rs117643180 is the primary signal implicating both post-challenge insulin resistance and expression of SLC2A4 at this locus (**Supplementary Table 17**).

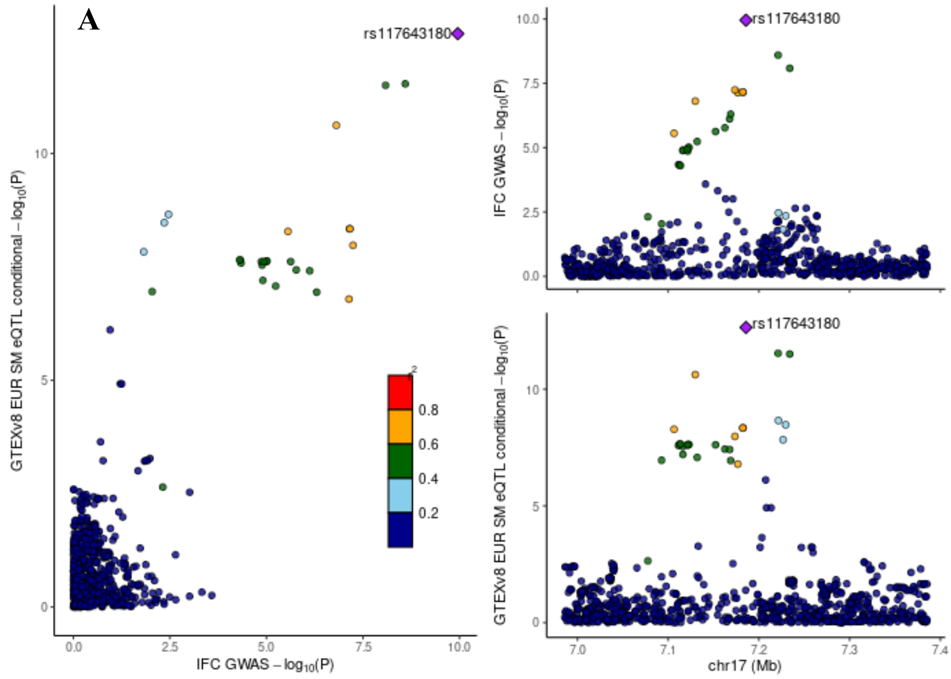

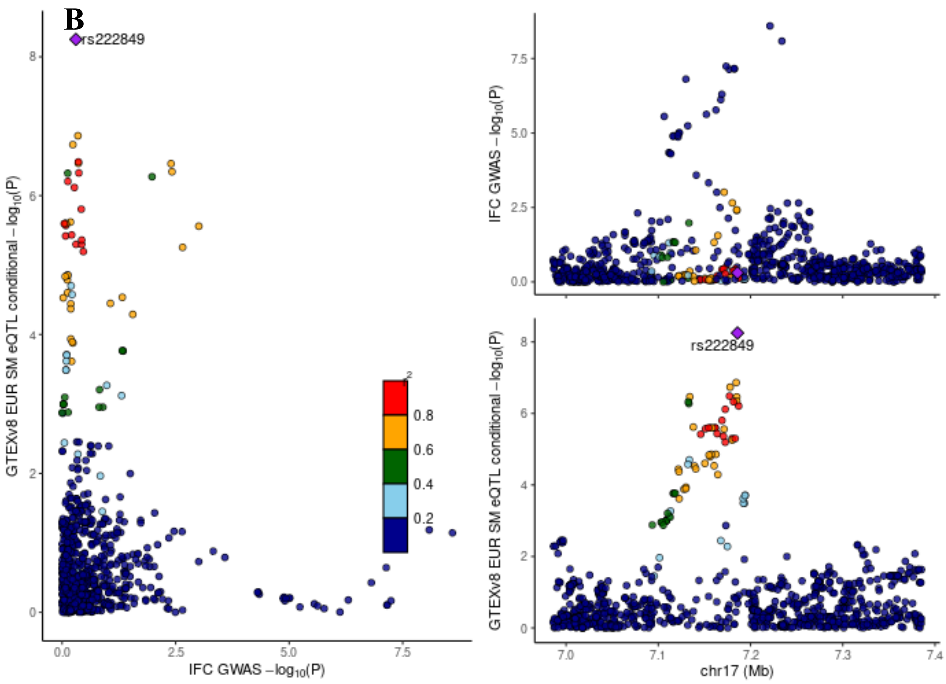
B
rs222849
GTEXv8 EUR SM eQTL conditional −log10(P)
IFC GWAS −log10(P)
IFC GWAS −log10(P)
chr17 (Mb)
0.8
0.6
0.4
0.2

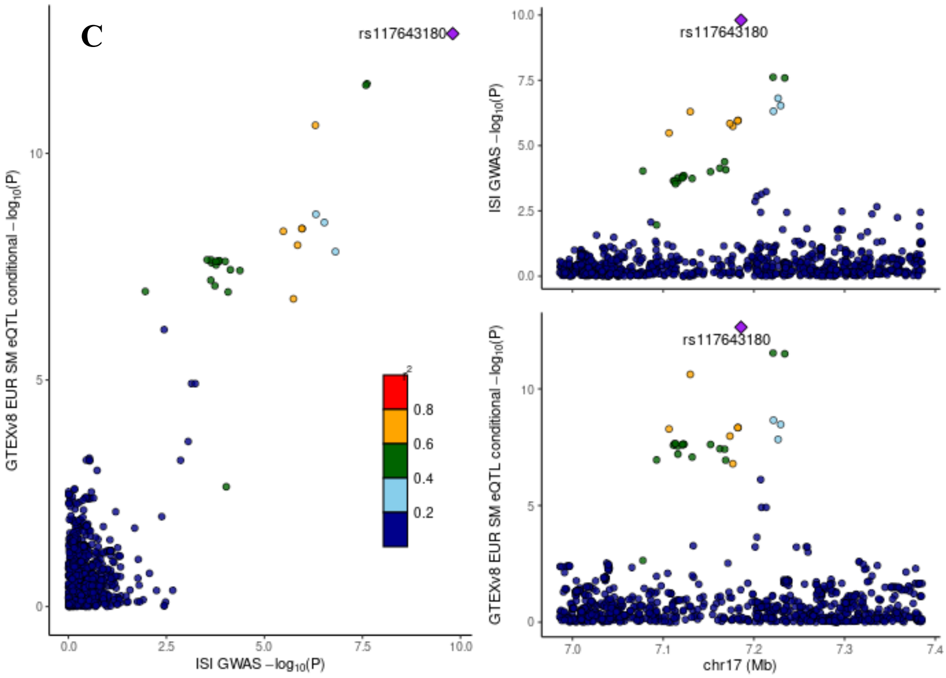
C
rs117643180
GTEXv8 EUR SM eQTL conditional −log10(P)
ISI GWAS −log10(P)
chr17 (Mb)
0.8
0.6
0.4
0.2

D

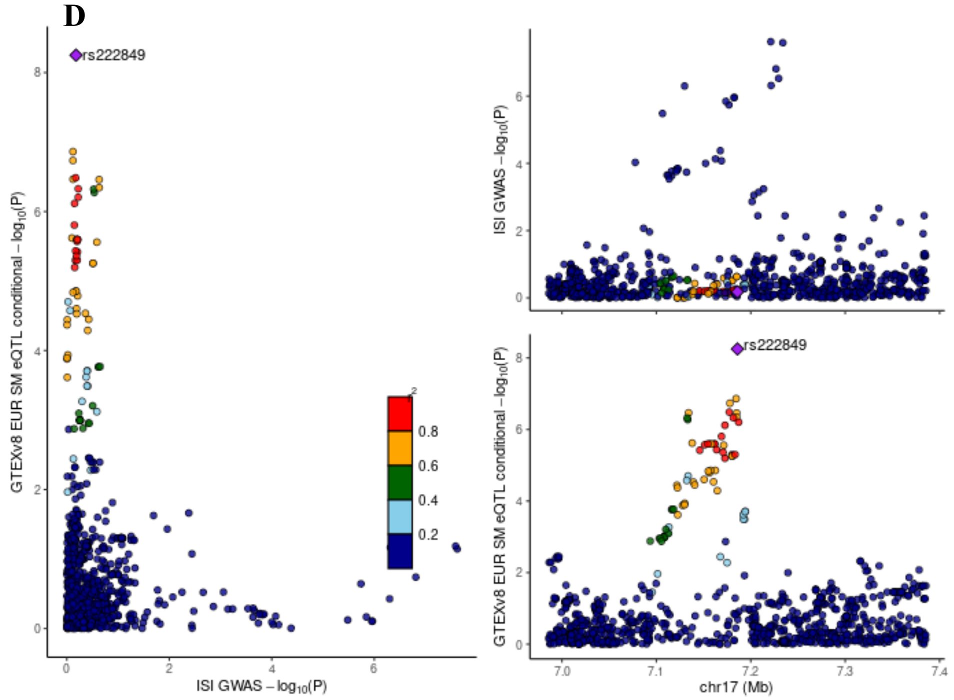

Supplementary Figure 17: statistical colocalisation of ISI/IFC with conditional summary statistics for eQTL in skeletal muscle at the SLC2A4 locus.

A) IFC and SLC2A4 eQTL statistics conditioned on the secondary signal rs222849. B) IFC and SLC2A4 eQTL statistics conditioned on the primary signal rs117643180. C) ISIand SLC2A4 eQTL statistics conditioned on the secondary signal rs222849. D) ISI and SLC2A4 eQTL statistics conditioned on the primary signal rs117643180. Locus was defined as ±200kb of rs117643180 for colocalisation analyses. The colour of points, as indicated in the legend, represent R^2 values compared to the lead SNP (1000 genomes European LD reference) for each signal annotated with a purple diamond. Unadjusted -log10 p-values are displayed.

10. rs117643180 (*SLC2A4*) affects expression of GLUT4 in skeletal muscle through changes in transcriptional regulation

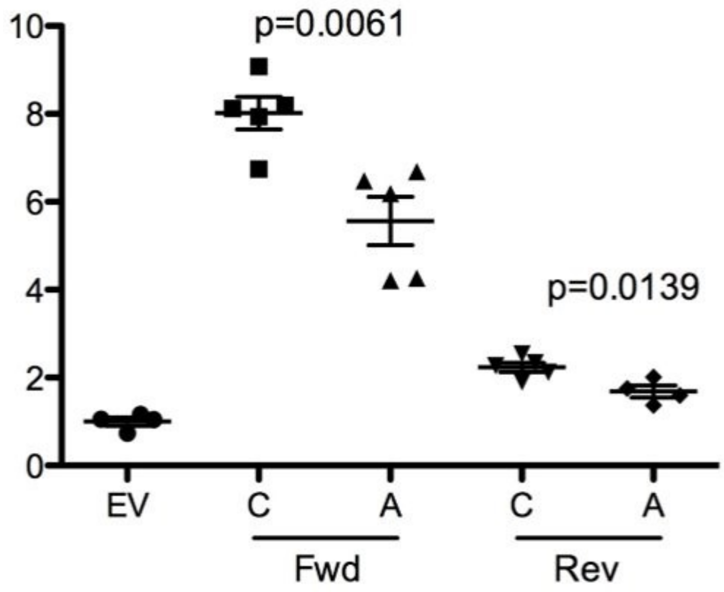

Supplementary Figure 18: rs117643180 exhibits allelic differences in transcriptional activity.
229-bp fragments flanking rs117643180-C or rs117643180-A were cloned upstream of a minimal promoter driving luciferase expression in the forward and reverse orientations with respect to the promoter. Values represent fold-change of firefly luciferase/*Renilla* activity normalized to empty pGL4.23 vector in undifferentiated LHCN-M2 myoblasts. Error bars represent the SEM of four or five independent clones tested in duplicate wells, individual points represent independent clones. P-values are calculated from two-sided t-tests.

11. Tissue of Action at post-challenge insulin reflects loci reflect tissues implicated in post-prandial insulin action.

To delineate potential tissue of action for post-challenge loci we employed LDSC-SEG to identify if the heritability of IFC or Modified Stumvoll ISI is enriched in regions surrounding genes with the highest specific tissue or cell type expression.

Modified Stumvoll ISI showed nominal genome-wide enrichment of active chromatin state in adipose, liver, skeletal muscle and pancreatic tissues, as well as nominal enrichment in tissue-specific gene expression in liver, muscle tissues and other T2D unspecific tissues (**Supplementary Tables 24 and 25**). Insulin fold change showed nominal enrichment in active chromatin marks in the liver, pancreas, and other tissues, as well as nominal enrichment in liver-specific gene expression. However, none of these annotations survives multiple testing corrections (**Supplementary Tables 22 and 23**).

12. Integration of additional phenotypic layers identifies additional loci implicated in post-challenge insulin action

Previous research has shown the utility of integrating information on associations with related traits when prioritising genetic loci associated with insulin resistance.[23] Here, we employed this approach to ISI and IFC with the aim to identify biologically relevant loci, with a post-challenge specific insulin resistance association pattern, which do not yet reach genome wide significance. This approach allowed us to identify additional loci of interest without sufficient sample size to detect these at a stringent genome wide significance threshold. To do this we prioritised variants that were suggestively associated with IFC and/or ISI ($P < 5x10^{-5}$). We next further filtered these variants based on their association with 2 h plasma glucose levels[7] ($P < 5x10^{-4}$) to prioritise those variants with additional evidence of being associated with post-challenge insulin resistance state. We additionally employed a second strategy where we filtered the variants suggestively associated with ISI and/or IFC based on their lack ($P < 0.05$) of association with fasting insulin, to remove those likely associated with insulin resistance in the fasting state, implicating the liver. These variants were further filtered based on evidence of being eQTLs in skeletal muscle ($P < 5x10^{-4}$).[24] This allowed us to prioritise those variants with evidence of regulating expression in skeletal muscle, a tissue of high biological relevance to postprandial insulin action. Loci which met both prioritisation criteria were considered to have a post-challenge insulin resistance specific association signature, with further evidence of regulatory effects in skeletal muscle, the key tissue in post-prandial glucose uptake.

Through this strategy we prioritised 10 loci, including 6 loci that were not identified at genome wide significance for either IFC and ISI; **Supplementary Table 19**).

The *ERAP2* locus (rs1216570) was associated with higher IFC and 2 h glucose[7], and lower ISI, suggestive of post-challenge insulin resistance (**Supplementary Figure 19a-b**. rs1216570 is an eQTL associated with higher *ERAP2* expression in skeletal muscle (GTEX v8 EUR: NES = 1.01, SE= 0.029, $P = 4.15x10^{-138}$). This eQTL in both muscle and adipose tissues, as well as 2hr glucose association has also been identified in other populations including in a cohort of African American ancestry[25], as well as a cohort from Sweden.[26] A muscle *ERAP2* eQTL at this

locus has additionally identified in the Pima Indian population.[27] However, we identified limited evidence of a shared genetic signal for this eQTL signal (GTEX v8 EUR) and IFC (posterior probability of colocalisation = 0.32).

The lead variant at this locus, rs1216570, is additionally an eQTL in skeletal muscle for *LNPEP (*GTEx v8 EUR: NES = -0.13, SE = 0.026, P = 3.92×10^{-7})[22] and there is strong evidence of colocalisation of this eQTL and IFC signal (posterior probability colocalisation = 0.85). *LNPEP* encodes IRAP, an insulin responsive amino peptidase that collocates with GLUT4 on GLUT4 secretory vesicles, and is well established to be involved in GLUT4 trafficking.[28] This demonstrates that this integrated approach identifies loci that are biologically relevant to post-challenge insulin resistance.

SHMT1 (rs7212808) was associated with a favourable glycaemic signature (**Supplementary Figure 19c-d**) and is additionally an eQTL for increased *LLGL1* expression in skeletal muscle (GTEx v8 EUR: NES = 0.15, SE = 0.028, P = 1.00×10^{-7} ; posterior probability of colocalisation IFC and LLGL1 eQTL = 0.91[22]). LLGL1 is a Rab GTPase activating protein that interacts with Rab10, a key player in insulin-stimulated GLUT4 translocation.[29] Although LLGL1 has not previously been implicated in post-challenge insulin response, it is plausible that this may play a role in regulating glucose uptake in the postprandial state.

All phenotypic signatures were additionally assessed using formal statistical colocalisation method hyprcoloc[30] (**Supplementary Figure 20**). We additionally tested for pairwise colocalisation of IFC and ISI signals with eQTLs in diabetes relevant tissues using coloc[31] (skeletal muscle, adipose, liver, and pancreatic islet; **see methods; Supplementary Table 20**). Further insight into tissue specificity of the genetic architecture of ISI and IFC was gained using LDSC-SEG, which leverages cell and tissue specific gene expression profiles to assess tissue specific enrichment of trait heritability (**see above; Supplementary Tables 21 to 25**).

By going below, the stringent genome wide significance level we identified evidence of loci directly implicating the biology of GLUT4 translocation in post-challenge insulin resistance comes from our findings at the *ERAP2* locus (rs1216570), suggestively associated (P = 1.01x10⁻

^{5}) with increased IFC and reduced ISI (P = $2.4x10^{-4}$), and suggestively associated with 2 h glucose (P = $2.64x10^{-5}$) but not fasting glycaemic measures. We identified evidence of a shared genetic signal for IFC and eQTL in skeletal muscle for *LNPEP* which encodes IRAP, the insulin responsive amino peptidase. IRAP is found on GLUT4 secretory vesicles and binds TBC1D4, to prevent translocation of GLUT4 to the plasma membrane in the fasting state (low insulin).[32] In line with previous reports that IRAP is a negative regulator of plasma membrane GLUT4[28], depletion of *LNPEP* in 3T3-L1 adipocytes increased GLUT4 translocation to the cell surface. The lead variant at this locus is further in perfect LD (R^2=1) in Europeans with a previously reported missense variant significantly associated with 2hr glucose (rs2549782)[33]. However, colocalisation of our lead variant rs1216570 with an *LNPEP* skeletal muscle eQTL, no evidence of an impact of *ERAP2* knockdown on GLUT4 trafficking in vitro, and in silico algorithms including SIFT[34], PolyPhen2[35] and CADD[36] predicting the missense variant to be benign and to be unlikely to have an impact on the function of the encoding protein, suggest that *LNPEP* rather than *ERAP2* may be the causal gene at this locus.

We further assessed potential tissue of action at individual associated loci by assessing colocalization of loci identified to be associated with IFC and Modified Stumvoll ISI in either the genetic discovery or integrated analyses with eQTLs in T2D relevant tissues – adipose, liver, pancreatic islets and skeletal muscle. We identified colocalization (posterior probability of colocalization > 0.7) at 3 loci associated with IFC at ERAP1/ERAP2, SHMT1 and IRS1 with eQTLs identified in skeletal muscle and subcutaneous and visceral adipose tissues, key tissues in the post-prandial insulin response (**Supplementary Table 20**).

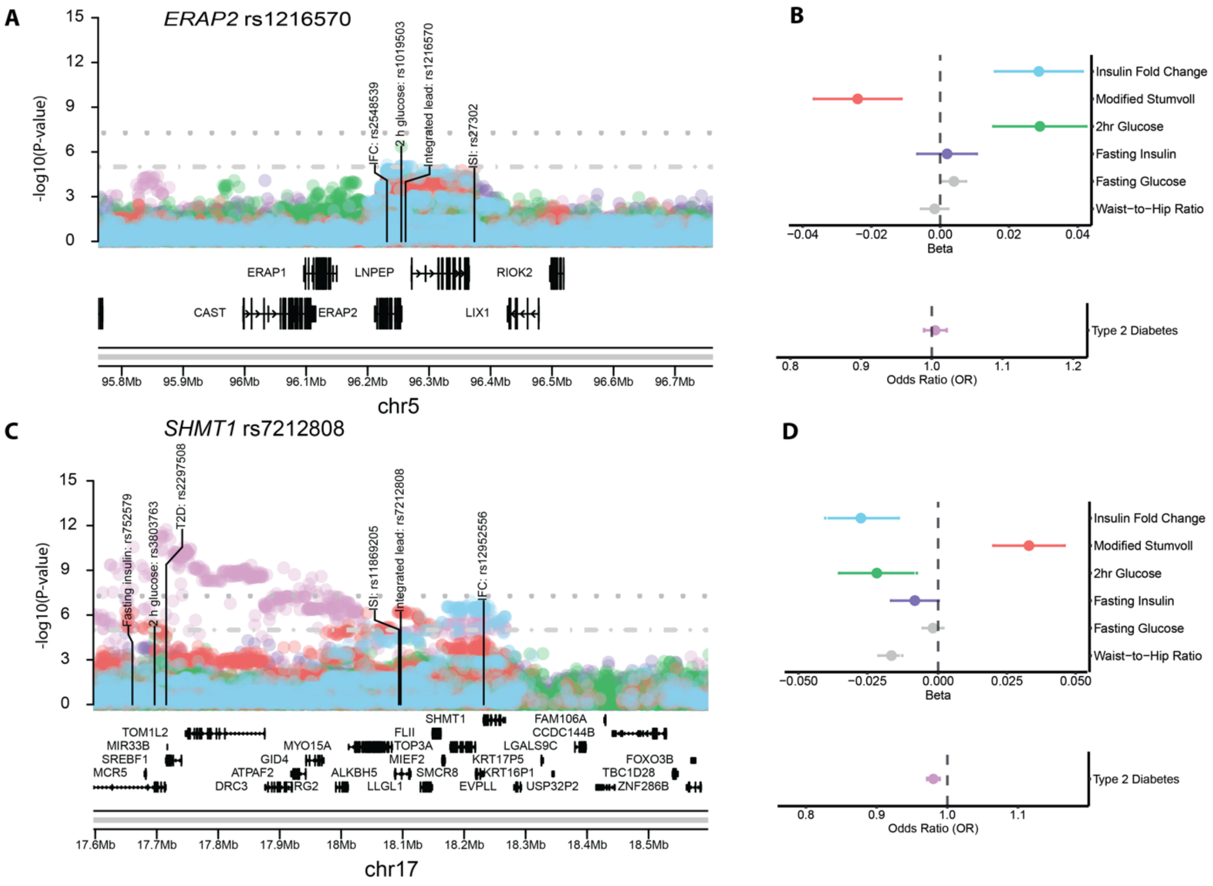

Supplementary Figure 19: Integrated GWAS approach highlights additional loci including those implicating glucose transport.

A) *ERAP2* locus: regional association plot for relevant traits. B) *ERAP2* locus: association of rs1216570 with relevant traits. **C)** *SHMT1* locus: regional association plot for relevant traits, with colours of points indicating traits of interest. **D**) *SHMT1* locus: association of rs7212808 with relevant traits. **A and C)** Regional association plot showing ± 500kb around the lead IFC-associated variant, with points indicating the association and location of individual variants. Colours of points indicating traits of interest: insulin fold change (IFC) - blue, Modified Stumvoll (ISI) – red, and 2 h glucose[7] – green, fasting insulin[7] – purple, type 2 diabetes[8] – pink . Y-axis denotes the unadjusted –$\log_{10}$(p-value) of association. The dashed lines and dotted line indicate genome-wide ($P = 5x10^{-8}$) and suggestive ($P = 1x10^{-5}$) significance threshold, respectively. X-axis denotes the genomic position. All association statistics are from BMI adjusted analyses in studies of European ancestry, except T2D which is unadjusted for BMI**.** The dashed lines indicate suggestive ($P = 1x10^{-5}$) significance threshold and dotted line indicates genome-wide significance ($P = 5x10^{-8}$), respectively. Labels indicate the lead variant for each trait, with location indicating position. Integrated lead is the lead variant defined for this locus using the integrated GWAS approach. **B and D)** Plot shows beta estimate or odds ratio of association for lead variant with insulin fold change (IFC) - blue, Modified Stumvoll (ISI) – red, and 2 h glucose[7] – green, fasting insulin[7] – purple, type 2 diabetes[8] – pink, all included in a) as well as fasting glucose[7] and waist-to-hip ratio[9] plotted in grey. All association statistics

are from BMI adjusted analyses in studies of European ancestry. Error bars indicate 95% CI of the effect size. Sample size is outlined in **Methods**.

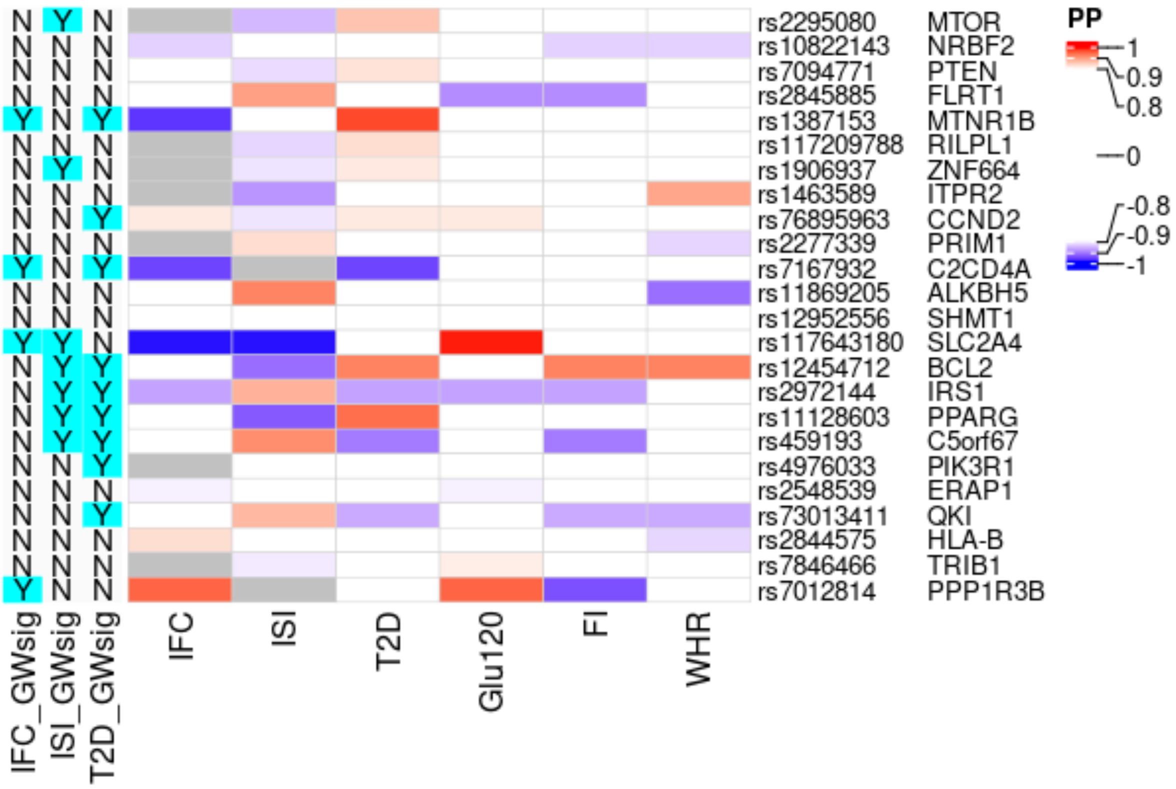

Supplementary Figure 20: Assessment of colocalisation at loci associated with IFC or ISI with related traits
Fasting insulin (FI), 2hr glucose (Glu120) type 2 diabetes (T2D) and waist-to-hip ratio (WHR). All summary statistics used as input are from European ancestry individuals and adjusted for BMI, except for T2D which is unadjusted for BMI (Methods). GWsig denotes whether the IFC/ISI lead SNP at a locus (indicated by the rsid) meets genome-wide significance for the indicated trait (unadjusted P < 5e-8). White denotes posterior probability < 0.8, with no evidence of colocalisation. Red positive beta for the indicated trait, the blue negative beta for the trait. Shade denotes the posterior probability of colocalisation between 0.8 and 1. Grey denotes that the trait was not included in hyprcoloc due to the locus (lead SNP +/- 500kb) not meeting the inclusion threshold of P < 5e-4 for that trait. All effects are aligned to the IFC effect allele.

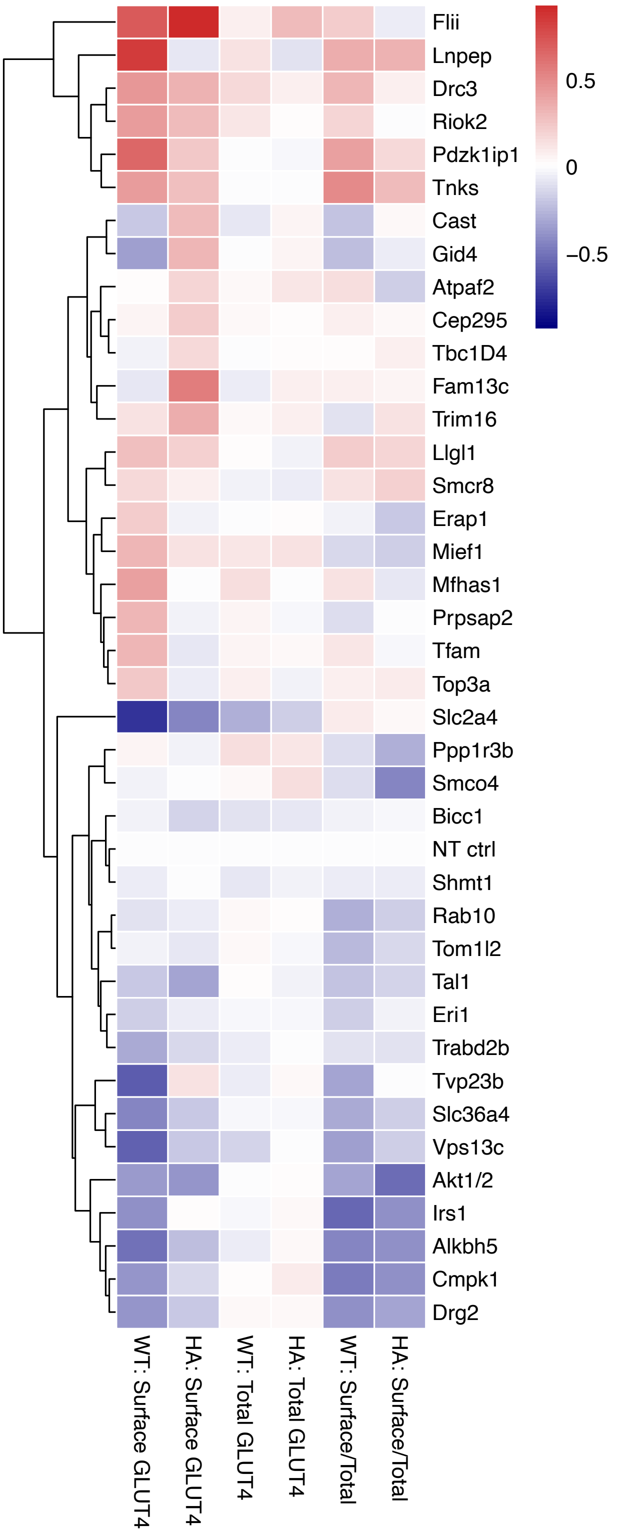

Supplementary Figure 21: GLUT4 screen results for 3T3L1 WT and 3T3L1 HA-GLUT4-mRuby 3T3-L1 adipocytes for all targets

All values are normalised to non-targeting control (NT) for the relevant measure/cell line combination. Targets with values lower than NT control are shown in blue and those with higher values in red. Clustering of targets was done using hierarchical clustering. Values are those measured at 0.5nM insulin. WT N = 3 biological replicates, with 2 technical replicates per N. HA – N = 5 biological replicates, with 2 technical replicates per N.

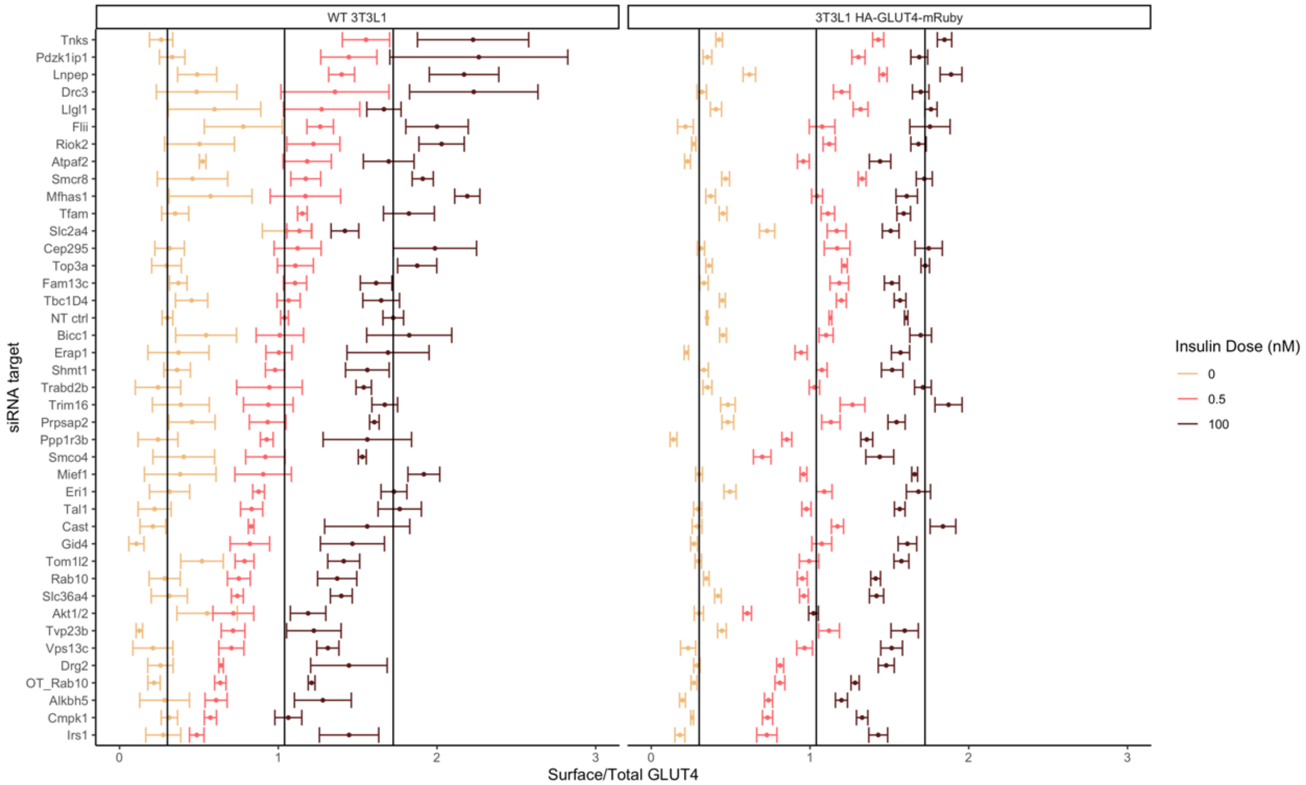

Supplementary Figure 22: Overall screen surface/total GLUT4 comparison for HA-GLUT4-mRuby and WT 3T3-L1 adipocytes.

Lines denote NT ctrl response at each dose line for WT 3T3L1 adipocytes. WT N = 3 biological replicates, with 2 technical replicates per N. HA-GLUT4-mRuby – N = 5 biological replicates, with 2 technical replicates per N. Insulin dose is indicated by colours in legend in nM. Error bar represents mean +/- SE.

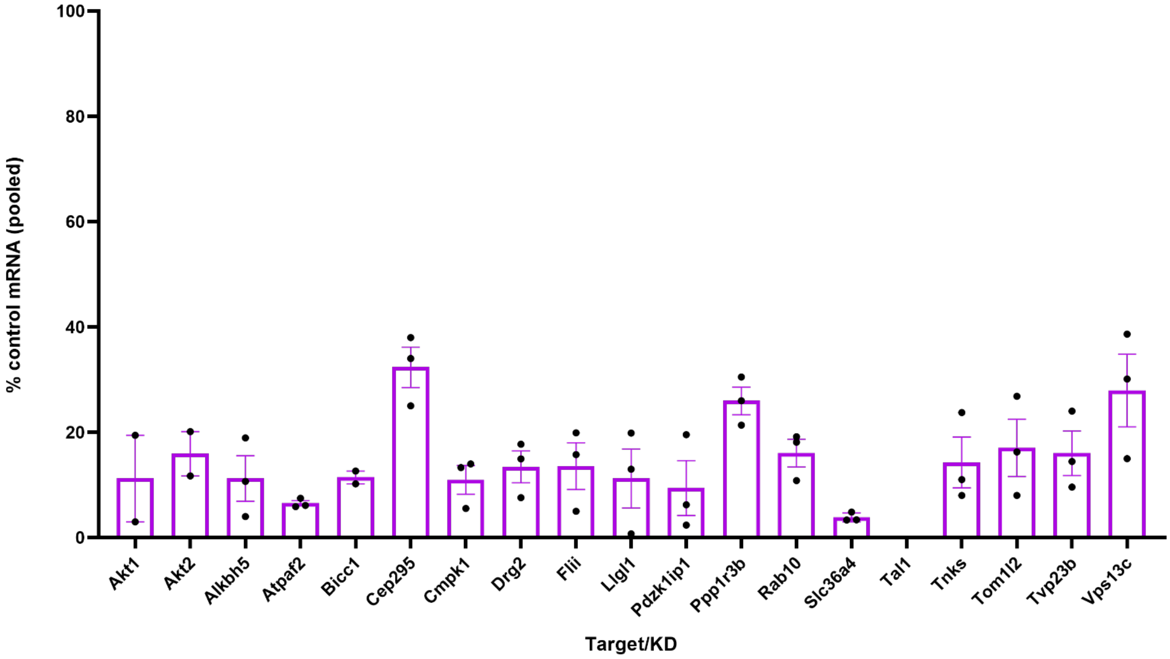

Supplementary Figure 23: Average percentage of mRNA detected in knockdown samples compared to NT control.

N = 2-3 independent biological replicates per gene, points represent independent samples. Error bar represents +/- standard error of mean.

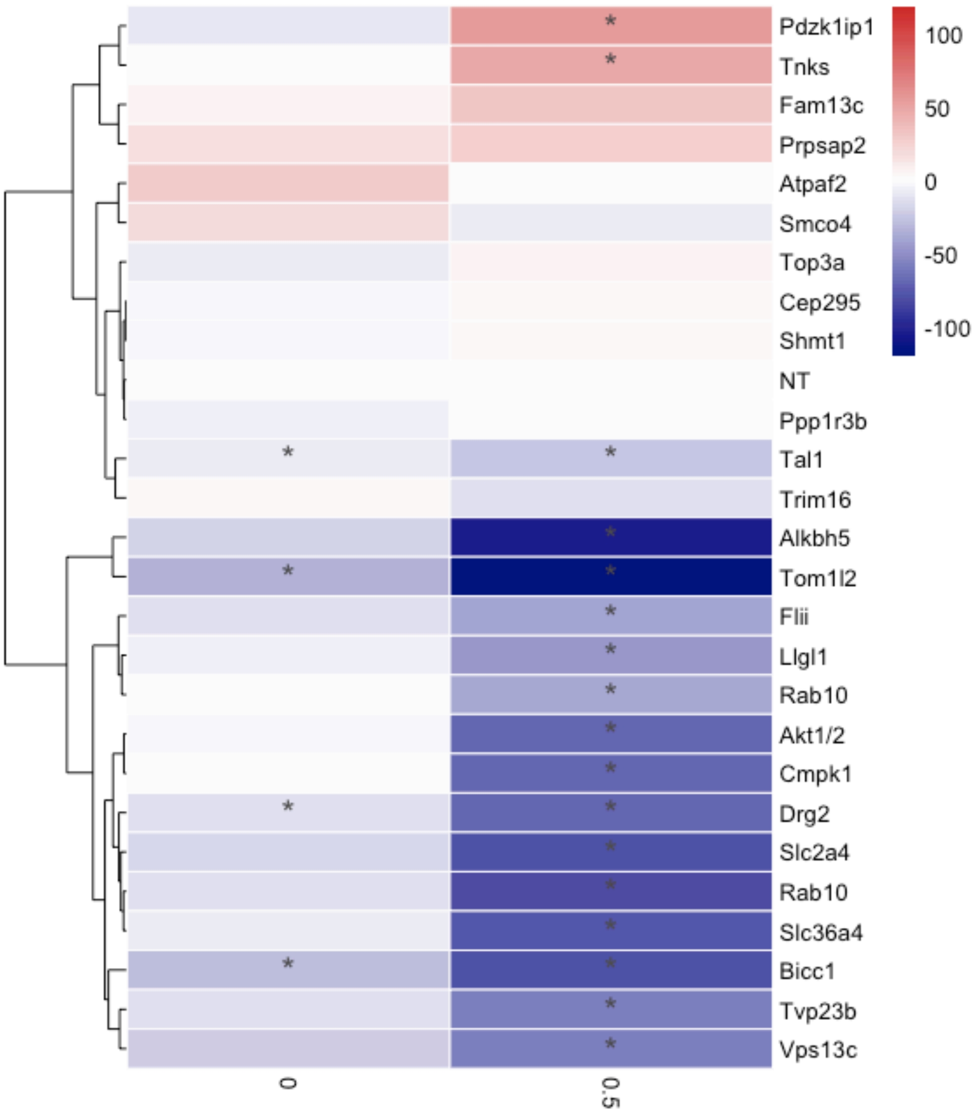

Supplementary Figure 24: Glucose Transport relative to NT control

Values are normalised glucose response to non-targeting control (NT; 0) per dose of insulin (nM), and scaled to the absolute maximal response expressed as percentage . * Indicates targets that are significantly different from the NT control at each dose (unadjusted $P < 0.05$; two-tailed t-test). N = 3 biological replicates per condition, except NT N = 6; Akt1/2 N = 6, Rab10 N = 6, Tvp23b N = 4. All replicates were successful.

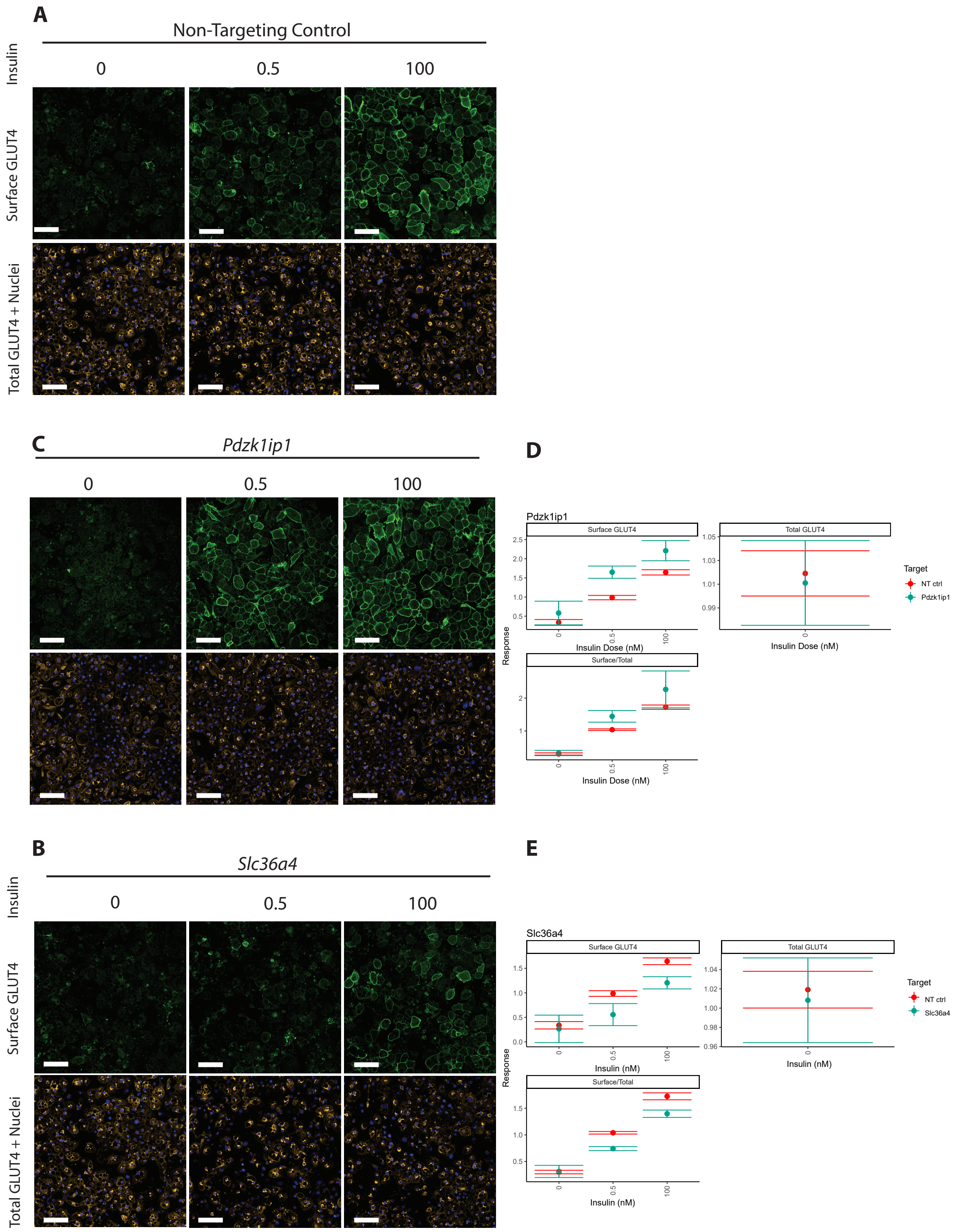

Supplementary Figure 25: Visualisation and quantification of impact on parameters of interest of knockdown Slc36a4 and Pdzk1ip1 in 3T3-L1 adipocytes.

A) Representative microscopy images of non-targeting control (NT ctrl) at 0,0.5 and 100nM insulin stimulation. Surface GLUT4 in green, total GLUT4 in orange, and nuclei in blue. The scale bar on each image represents 100µm. B) Representative microscopy images of Pdzk1ip1 knockdown. As outlined in a). C) Representative microscopy images of Slc36a4 knockdown. As outlined in a). D and E) quantification of the effect of knockdown on parameters of interest, normalised to background control. Response is relative to NT at a given insulin concentration, as indicated on X axis. Green – target, red- NT control. Error bars represent mean +/- SE.

References for Supplementary Note:

1. Bycroft, C. *et al.* The UK Biobank resource with deep phenotyping and genomic data. *Nature* **562**, 203–209 (2018).
2. Lindsay, T. *et al.* Descriptive epidemiology of physical activity energy expenditure in UK adults (The Fenland study). *Int. J. Behav. Nutr. Phys. Act.* **16**, (2019).
3. Pietzner, M. *et al.* Genetic architecture of host proteins involved in SARS-CoV-2 infection. *Nat. Commun. 2020 111* **11**, 1–14 (2020).
4. Powell, R. *et al.* Development and validation of total and regional body composition prediction equations from anthropometry and single frequency segmental bioelectrical impedance with DEXA. *medRxiv* 2020.12.16.20248330 (2020) doi:10.1101/2020.12.16.20248330.
5. Lam, B. Y. H. *et al.* MC3R links nutritional state to childhood growth and the timing of puberty. *Nat. 2021 5997885* **599**, 436–441 (2021).
6. Bulik-Sullivan, B. *et al.* LD Score regression distinguishes confounding from polygenicity in genome-wide association studies. *Nat. Genet. 2015 473* **47**, 291–295 (2015).
7. Chen, J. *et al.* The trans-ancestral genomic architecture of glycemic traits. *Nat. Genet. 2021 536* **53**, 840–860 (2021).
8. Vujkovic, M. *et al.* Discovery of 318 new risk loci for type 2 diabetes and related vascular outcomes among 1.4 million participants in a multi-ancestry meta-analysis. *Nat. Genet. 2020 527* **52**, 680–691 (2020).
9. Pulit, S. L. *et al.* Meta-analysis of genome-wide association studies for body fat distribution in 694 649 individuals of European ancestry. *Hum. Mol. Genet.* **28**, 166–174 (2019).
10. Finucane, H. K. *et al.* Heritability enrichment of specifically expressed genes identifies disease-relevant tissues and cell types. *Nat. Genet.* **50**, 621 (2018).
11. Roadmap Epigenomics Consortium *et al.* Integrative analysis of 111 reference human epigenomes. *Nature* **518**, 317–329 (2015).
12. Dunham, I. *et al.* An integrated encyclopedia of DNA elements in the human genome. *Nature* **489**, 57–74 (2012).
13. Diaz-Vegas, A. *et al.* A high-content endogenous GLUT4 trafficking assay reveals new aspects of adipocyte biology. *Life Sci. alliance* **6**, (2023).
14. Willer, C. J., Li, Y. & Abecasis, G. R. METAL: Fast and efficient meta-analysis of genomewide association scans. *Bioinformatics* **26**, 2190–2191 (2010).
15. Wang, Q. *et al.* Rare variant contribution to human disease in 281,104 UK Biobank exomes. *Nat. 2021 5977877* **597**, 527–532 (2021).
16. American Diabetes Association. 2. Classification and diagnosis of diabetes: Standards of medical care in diabetes-2021. *Diabetes Care* **44**, S15–S33 (2021).
17. Spracklen, C. N. *et al.* Identification of type 2 diabetes loci in 433,540 East Asianindividuals. *Nature* **582**, 240 (2020).
18. Williams, A. L. *et al.* Sequence variants in SLC16A11 are a common risk factor for type 2 diabetes in Mexico. *Nature* **506**, 97–101 (2014).
19. Karczewski, K. J. *et al.* The mutational constraint spectrum quantified from variation in 141,456 humans. *Nature* **581**, 434–443 (2020).
20. Auton, A. *et al.* A global reference for human genetic variation. *Nature* **526**, 68–74 (2015).
21. Machiela, M. J. & Chanock, S. J. LDlink: a web-based application for exploring

population-specific haplotype structure and linking correlated alleles of possible functional variants. *Bioinformatics* **31**, 3555–3557 (2015).
22. Aguet, F. *et al.* The GTEx Consortium atlas of genetic regulatory effects across human tissues. *Science (80-.).* **369**, 1318–1330 (2020).
23. Lotta, L. A. *et al.* Integrative genomic analysis implicates limited peripheral adipose storage capacity in the pathogenesis of human insulin resistance. *Nat Genet* **49**, 17–26 (2017).
24. Lonsdale, J. *et al.* The Genotype-Tissue Expression (GTEx) project. *Nature Genetics* vol. 45 580–585 (2013).
25. Sajuthi, S. P. *et al.* Mapping adipose and muscle tissue expression quantitative trait loci in African Americans to identify genes for type 2 diabetes and obesity. *Hum. Genet.* **135**, 869–880 (2016).
26. Keildson, S. *et al.* Expression of Phosphofructokinase in Skeletal Muscle Is Influenced by Genetic Variation and Associated With Insulin Sensitivity. *Diabetes* **63**, 1154–1165 (2014).
27. Mason, C. C. *et al.* Bimodal distribution of RNA expression levels in human skeletal muscle tissue. *BMC Genomics* **12**, (2011).
28. Jordens, I., Molle, D., Xiong, W., Keller, S. R. & McGraw, T. E. Insulin-regulated aminopeptidase is a key regulator of GLUT4 trafficking by controlling the sorting of GLUT4 from endosomes to specialized insulin-regulated vesicles. *Mol. Biol. Cell* **21**, 2034–2044 (2010).
29. Sano, H. *et al.* Rab10, a target of the AS160 Rab GAP, is required for insulin-stimulated translocation of GLUT4 to the adipocyte plasma membrane. *Cell Metab.* **5**, 293–303 (2007).
30. Foley, C. N. *et al.* A fast and efficient colocalization algorithm for identifying shared genetic risk factors across multiple traits. *Nat. Commun. 2021 121* **12**, 1–18 (2021).
31. Giambartolomei, C. *et al.* Bayesian Test for Colocalisation between Pairs of Genetic Association Studies Using Summary Statistics. *PLOS Genet.* **10**, e1004383 (2014).
32. Rubio-Sastre, P., Scheer, F. A. J. L., Gómez-Abellán, P., Madrid, J. A. & Garaulet, M. Acute melatonin administration in humans impairs glucose tolerance in both the morning and evening. *Sleep* **37**, 1715-1719B (2014).
33. Ng, N. H. J. *et al.* Tissue-Specific Alteration of Metabolic Pathways Influences Glycemic Regulation. *bioRxiv* **29**, 790618 (2019).
34. Sim, N.-L. *et al.* SIFT web server: predicting effects of amino acid substitutions on proteins. *Nucleic Acids Res.* **40**, W452–W457 (2012).
35. Adzhubei, I. A. *et al.* A method and server for predicting damaging missense mutations. *Nat. Methods* **7**, 248–249 (2010).
36. Rentzsch, P., Witten, D., Cooper, G. M., Shendure, J. & Kircher, M. CADD: predicting the deleteriousness of variants throughout the human genome. *Nucleic Acids Res.* **47**, D886–D894 (2019).
